# Introducing TAPY as a Versatile Alternative to TPP for Selective Mitochondrial Targeting in Cancer Cells

**DOI:** 10.1021/acs.bioconjchem.4c00554

**Published:** 2025-03-31

**Authors:** Jean C. Neto, Federico Lucantoni, Leydy V. González, Eva Falomir, Juan F. Miravet, Francisco Galindo

**Affiliations:** † 16748 Universitat Jaume I de Castellón, Departamento de Química Inorgánica y Orgánica, Avda. Vicente Sos Baynat s/n, 12071 Castellón de la Plana, Spain; ‡ Laboratory of Cellular Stress and Cell Death Pathways, Centro de Investigación Príncipe Felipe (CIPF), 46012 Valencia, Spain

## Abstract

The understanding of diseases such as cancer and Alzheimer’s, along with natural aging processes, heavily relies on the study of mitochondrial function. Optical techniques like fluorescence imaging microscopy are pivotal for this purpose, enabling precise mapping of subcellular structures, including mitochondria. In this study, we explored TAPY (triarylpyridinium) cations, a novel family of mitochondrial carriers resembling the well-known triphenylphosphonium cation (TPP). Six TAPY-bodipy (BDP) dyads were prepared and chemically characterized. Confocal Laser Scanning Microscopy (CLSM) studies demonstrated that the systems were delivered selectively to the mitochondria of cancer cells (MCF-7, A549, HT-29). Remarkably, these dyads did not target the mitochondria of normal cells (HEK-293, HMEC-1), suggesting their potential use in distinguishing cancerous cells from healthy ones. A model compound comprised of the same bodipy cargo but attached to TPP was also synthesized and tested. Notably, in preliminary comparative assays with MCF-7 cells, the dyad TAPY­(OMe)-BDP outperformed the TPP derivative in mitochondrial imaging, achieving twice the final fluorescence intensity. The potential chemical diversity achievable with TAPY cations is considerable, with many derivatives being accessible starting from readily available commercial products. This implies that, based on the strategy outlined in this study, carefully optimized TAPY derivatives for targeted mitochondrial delivery could potentially be developed in the future as alternatives or complements to TPP, with the present work acting as a proof of concept.

## Graphical abstract

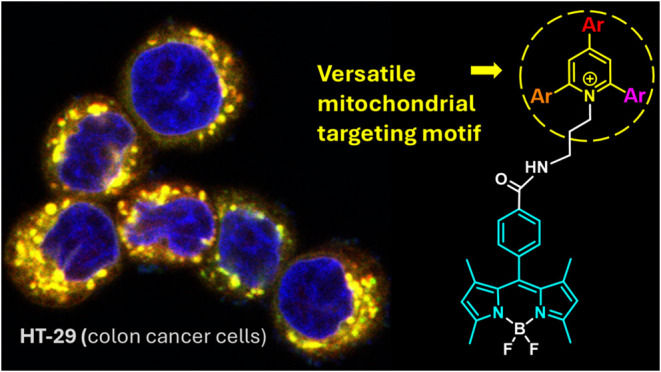

## 1 Introduction

The mitochondrion is a highly specialized organelle found in eukaryotic cells. While its primary role involves energy production through the synthesis of ATP via oxidative phosphorylation (OXPHOS), it also participates in various other essential biological processes, including calcium homeostasis, fatty acid β-oxidation, and amino acid metabolism. Notably, mitochondria are central to understanding the mechanisms underlying numerous diseases, as critical cellular events such as apoptosis and the excessive production of reactive oxygen species (ROS), which leads to cellular stress, are initiated within this organelle.^1^ Consequently, mitochondria have emerged as a major focus of research in the context of various pathologies, including cancer, Alzheimer’s disease, amyotrophic lateral sclerosis, heart failure, immunological disorders, and diabetes, among others.^2,3^ Aging has also become a topic of intense research in recent years, with mitochondria playing a prominent role in this field as well.^4^ All of the above has contributed to coining the term “Mitochondrial Medicine”, which refers to targeted approaches aimed at treating diseases by specifically targeting mitochondria.^5^

A diverse array of chemical tools has been employed for the detailed study of mitochondria, with fluorescent probes standing out due to their versatility and ease of use.^6,7^ The pioneering work of L. B. Chen, utilizing rhodamine 123 (Rh123) and other molecules, has been instrumental in advancing mitochondrial research.^8^ This work has led to the development of extensive collections of fluorescent molecular probes, which have been documented in numerous review articles.^9–12^ It is widely recognized that certain organic cations with lipophilic properties tend to accumulate in mitochondria, driven by the organelle’s negative membrane potential. The term “delocalized lipophilic cation” (DLC) is frequently used to describe these molecules with mitochondrial affinity.^13^ It is important to note, however, that not all systems reported to access mitochondria exhibit the characteristics of a DLC .^14,15^ In fact, a significant number of molecules that target this organelle are not cationic at all.^9^ Considering DLC as a synonym for mitochondrial locator could be misleading, as the accumulation of a molecule in this organelle results from a combination of factors that are not yet fully understood. Many questions remain in this context. Selected examples of positively charged molecules successfully used as fluorescent markers for mitochondria include the aforementioned Rh123, nonyl acridine orange (NAO),^16^ tetramethylrhodamine methyl ester (TMRM),^10^ coumarin derivatives,^17^ cationic napththalene derivatives such as MitoBlue,^18,19^ a plethora bodipys,^12^ and numerous cyanine dyes such as DiOC6(3), JC-1, and IR-780.^20^

Another strategy for targeting a specific chemical compound to the mitochondria is to covalently link it to a mitochondrial vector or tag, based on the hypothesis that the entire molecule would retain the mitochondriotropic properties of the attached tag.^10^ Although the properties of the entire molecule may not correspond to those of the carrier,^9^ this type of mitochondrial vector has become highly popular, not only in fluorescence bioimaging but also in mitochondria-directed therapy. Undoubtedly, this strategy has proven successful for some vectors, as it has enhanced the efficacy of mitochondrial drugs by concentrating them at their site of greatest effect and preventing their dispersion to other cellular locations. Examples of mitochondrial vectors include, unsurprisingly, systems with DLC features, such as trialkylammonium,^21^ guanidinium,^22^ indolinium,^23^ berberinium,^24^ and, among them, the triphenylphosphonium cation (TPP), which stands out for its simplicity and efficiency.^25^ TPP has been linked to a variety of molecules, some of which have led to the development of mitochondrial trackers^26^ or sensitive probes for mitochondrial pH,^27^ potassium,^28^ copper­(I),^29^ superoxide,^30^ and more. In the therapeutic realm, TPP has also been linked to bioactive agents such as antioxidants,^31^ anticancer drugs,^32^ and photosensitizers for photodynamic therapy.^33^

Although TPP has been used for years to target the mitochondria, very few structural modifications of this tag have been proposed. Notable exceptions, which aim to optimize the hydrophilic/hydrophobic balance for improved mitochondrial access, have been published.^34–37^ Additionally, modifications to the TPP scaffold to minimize interference with OXPHOS have been reported.^38^ However, these approaches generally focus on altering the original phosphorus derivative in one way or another.

We believe that a new approach could be explored, starting from a different scaffold resembling TPP, but with broader structural possibilities. Over the past decade, we have synthesized triarylpyrylium and diarylstyrylpyrilium cations, studying them in diverse realms such as fluorescence chemosensing,^39,40^ nanoparticle characterization^41^ supramolecular chemistry,^42,43^ optical devices,^44^ and cellular imaging.^45,46^

In this context, the affinity of this class of dyes for mitochondria in living cells has been demonstrated. Based on this observation, we hypothesized that conjugating a triarylpyrylium cation to a cargo of interest would result in the vectorization of the entire conjugate to the mitochondria, similar to how TPP conjugates direct drugs and probes to this organelle. This strategy would enable a broad structural diversity of tags, given the ease of synthesizing these cations from readily available commercial products (such as substituted benzaldehydes and acetophenones). The association of the pyrylium with a cargo (either a drug or a fluorescent group) could then be easily achieved using straightforward synthetic procedures, via the corresponding pyridinium cations, as illustrated in Figure 1. This approach would yield a TAPY-cargo conjugate, where TAPY stands for triarylpyridinium.

**Figure 1:**
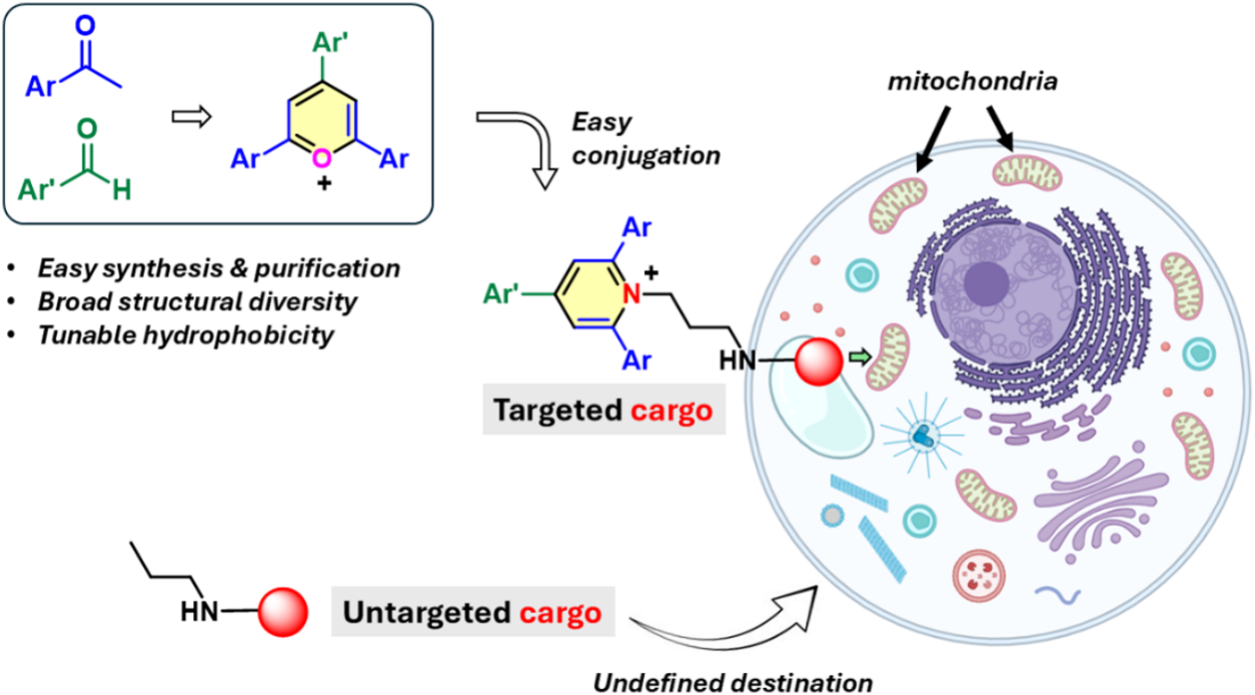
General strategy followed in this work to produce TAPY-cargo conjugates directed to the mitochondria (TAPY stands for TriArylPyridinium).

With this hypothesis in mind, we synthesized the six dyads shown in Figure 2. The TAPYs, with different substituents (R being electron-donor or electron-withdrawing), were conjugated to a generic cargo, such as a bodipy (BDP) fluorophore. A control system, not directed to the mitochondria, was also prepared in the form of a bodipy derivative with a propyl chain. The primary objective of this investigation was to assess the feasibility of using TAPY scaffolds as vectors for mitochondrial targeting. The second objective was to evaluate the influence of the substituent R, located on one of the rings, on the efficiency of vectorization— in other words, the role of the hydrophobicity of the TAPY moiety in this process. The third objective was to study the ability of these TAPY-bodipy dyads to selectively internalize into cancer cells over normal cells.

**Figure 2:**
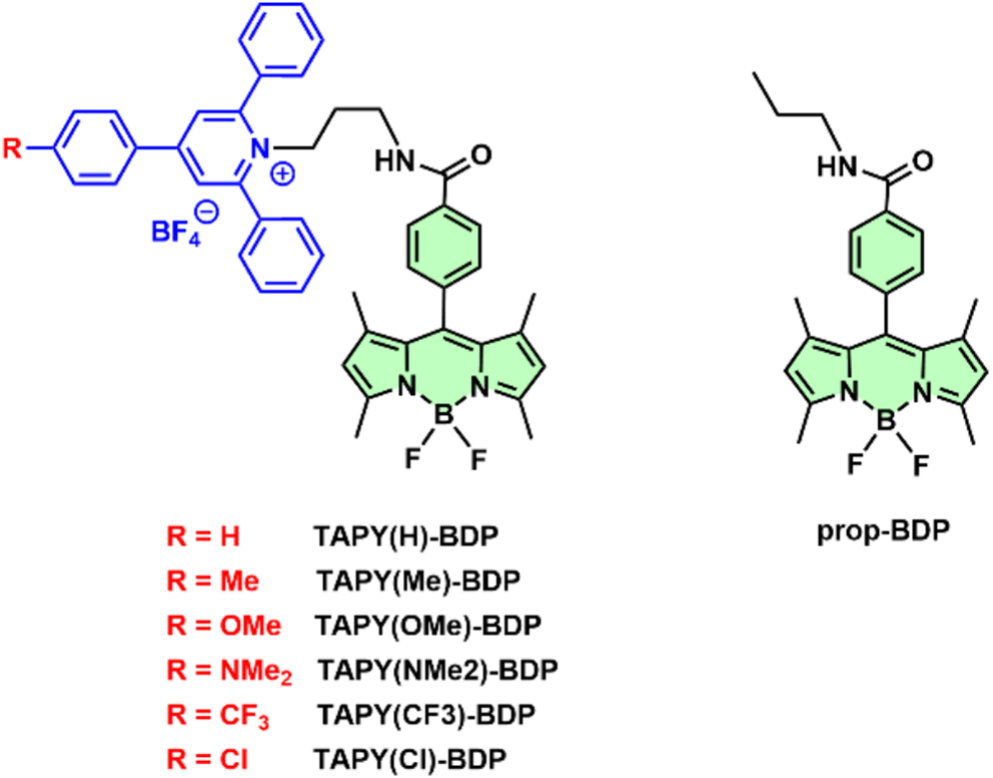
Chemical structures of the synthesized and studied compounds. **TAPY-BDP** dyads **TAPY­(H)-BDP**, **TAPY­(Me)-BDP**, **TAPY­(OMe)-BDP**, **TAPY­(NMe**_**2**_ **)-BDP**, **TAPY­(CF**_**3**_ **)-BDP**, **TAPY­(Cl)-BDP** and **prop-BDP**.

## 2 Results and Discussion

### 2.1 Synthesis

The synthesis of the dyads began with the preparation of the corresponding pyrylium structures (**1a**–**f**), as shown in Scheme S1 (Electronic Supporting Information). The reaction with 1,3-diaminopropane then yielded the corresponding pyridinium structure with a pendant amino group (**2a**–**f**). Finally, coupling with the bodipy fluorophore (in the form of **BDP-COOH**) provided the desired **TAPY-BDP** dyads. One of the key advantages of this synthesis is its ability to produce a wide variety of architectures starting from commercially available aromatic aldehydes and ketones, as demonstrated in previous synthetic examples.^41^ Characterization by ^1^H NMR, ^13^C NMR, and HR-MS confirmed the identity of the synthesized molecules (details on the synthesis of the **TAPY-BDP** dyads and the model compound **prop-BDP** can be found in the Materials and Methods section and within the Electronic Supporting Information; Figures S1–S21). It is worth noting that the range of TAPY derivatives that can be obtained using this strategy is extensive, as the careful selection of aromatic aldehydes and ketones in the first stage enables the preparation of tailor-made molecules with finely tuned hydrophobic/hydrophilic properties (and even optical properties, if TAPY’s own emission is intended for use). The six examples presented here are merely a representative sample of the molecules that can be easily synthesized.

### 2.2 Optical Characterization

The absorption and emission spectra of the seven compounds were recorded, as shown in Figure S22. In this figure, it can be seen that the absorption and emission of the bodipy chromophore in acetonitrile are observed at 498 and 514 nm, respectively, as expected for this type of structure.^47,48^ Interestingly, the presence of TAPY can also be observed, with absorption bands at 305 nm (**TAPY­(H)-BDP**), 315 nm (**TAPY­(Me)-BDP**), 315 nm (**TAPY­(OMe)-BDP**), 350 nm (**TAPY­(NMe**_**2**_ **)-BDP**), 290 nm (**TAPY­(CF**_**3**_ **)-BDP**), and 310 nm (**TAPY­(Cl)-BDP**). Since TAPY groups are also photoactive and have been used in photocatalysis,^49^ phototherapy,^50^ and sensing,^51^ it is foreseeable that further development of the dyads could lead to applications where dual excitation and emission are valuable (e.g., in ratiometric sensing). In contrast, the TPP cation is not suitable for optical applications, as its UV–vis absorption is reported below 280 nm.^52^

### 2.3 Toxicity Studies

The toxicity of the synthesized TAPY derivatives was assessed in the five cell cultures tested. As shown in Figure S23, cell viability remained at 90% or higher for concentrations up to 100 μM with incubation periods of 5 h. Given that typical bioimaging assays use significantly lower concentrations (0.5 μM) and much shorter incubation times (30 min), no toxicity is expected under standard conditions.

### 2.4 Confocal Microscopy Studies

The six **TAPY-BDP** dyads and the model compound **prop-BDP** were incubated with MCF7 breast cancer cells at a concentration of 0.5 μM for 30 min. Confocal laser scanning microscopy (CLSM) imaging of the cells with excitation at 488 nm revealed labeling of perinuclear structures consistent with mitochondria (additionally, the nucleus was labeled in blue with Hoechst 33342, as shown in Figure 3). In the same culture, the well-known mitochondrial marker Mitotracker Deep Red FM (MTDR) was also incubated, showing a similar staining pattern (Figure 3). The overlay of the green (BODIPY) and red (MTDR) channels resulted in a yellow/orange coloration in areas of effective colocalization, while red and green structures coexisted in other regions. As shown in Figure 3a, there was remarkable colocalization of all **TAPY-BDP** dyads with MTDR. Additionally, high Pearson correlation coefficients were calculated to confirm this observation. For example, **TAPY­(OMe)-BDP** colocalized with MTDR with a coefficient of 1.0. This result suggests that the cationic TAPY structures target the bodipy cargo to the mitochondria. The **prop-BDP** model compound, lacking a vectoring agent, showed a merged image with red and green dots inside the cell, indicating no mitochondrial localization. Two zoomed images in Figure 3b illustrate the dramatic difference between **TAPY­(H)-BDP** and **prop-BDP**.

**Figure 3:**
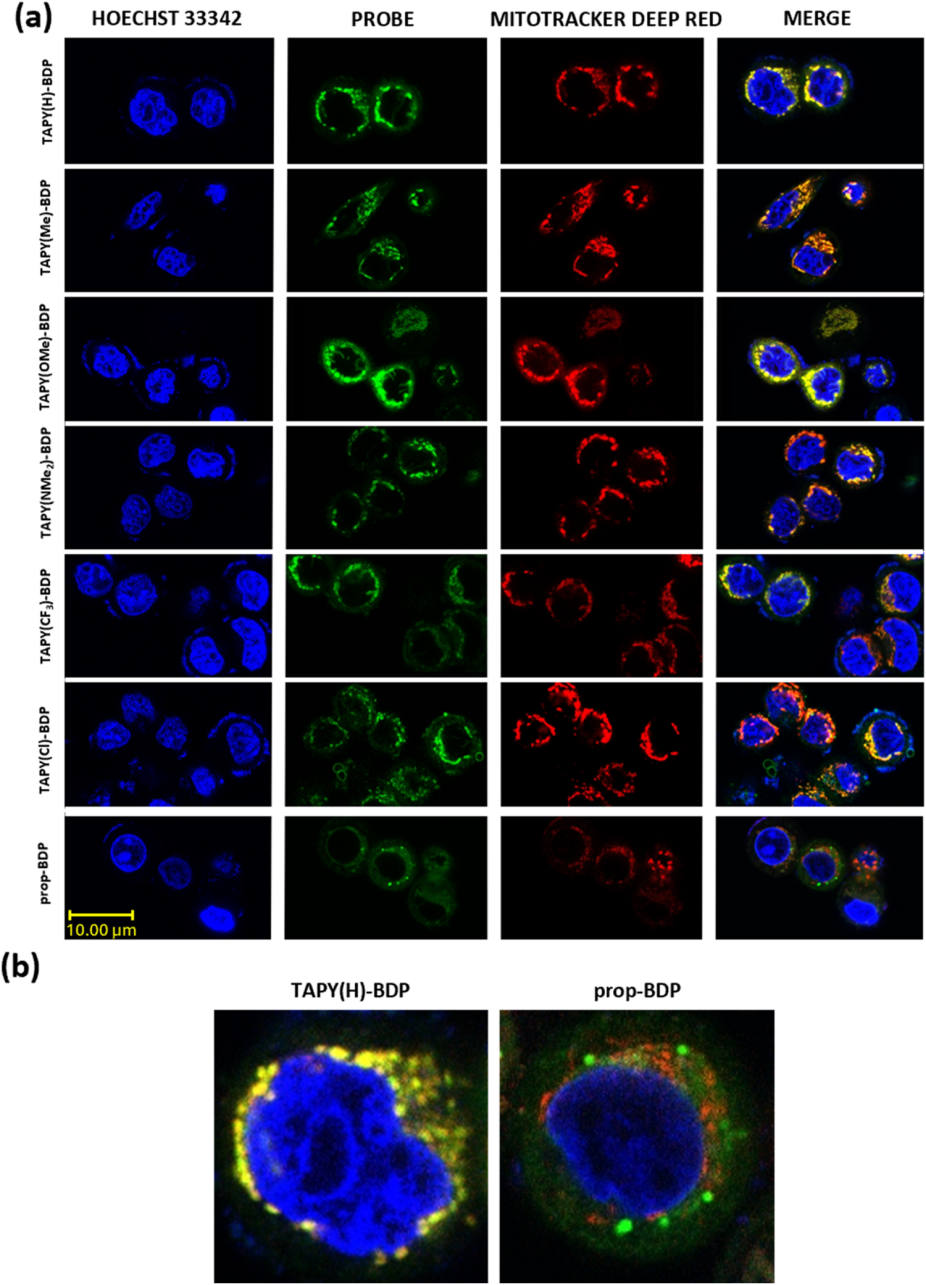
(a) CLSM images of MCF7 cells incubated with 0.5 μM of **TAPY-BDP**, and **prop-BDP** probes, 100 nM of Mitotracker Deep Red, and Hoechst 33342 for 30 min at 37 °C. Blue channel: excitation with 405 nm laser (Hoechst 33342); green channel: excitation with 488 nm laser (**TAPY-BDP** dyads and model compound); red channel: excitation with 633 nm laser (Mitotracker Deep Red FM). Also shown the overlay of green and red channels (merge). (b) Zoom of selected images (merge of green and red channels).

These results are consistent with recent studies from several research groups, including our own.^45,46^ We have demonstrated that emissive 2,4,6-triarylpyrylium and 2,4-diaryl-6-styrylpyrylium cations are effective mitochondrial fluorescent stains for various cell types. Not only do they efficiently label the mitochondria, but they also serve as sensors for analytes of interest within this organelle, such as nitric oxide (NO).^46,39^ Other examples of arylpyrylium and arylpyridinium dyes have also been used for cell imaging, particularly for mitochondrial staining.^53,54^

To further validate the effectiveness of TAPYs as mitochondrial vectors, the six dyads and the model compound were incubated with other cancer cell types, including A549 (lung cancer) and HT-29 (colon adenocarcinoma), and examined by CLSM. The resulting images were very similar to those obtained for MCF7, as shown in Figures S24 and S25, with **TAPY-BDP** dyads exhibiting a high degree of colocalization with MTDR. This suggests that the developed dyads are versatile and effective at targeting the mitochondria of various cancer cell types.

Morphology and size are factors that can provide insights into the physiological state of the cells, making it crucial to have probes capable of capturing images with the highest possible quality. This is nowadays especially important since artificial intelligence (AI) tools are used not only for data interpretation but also for the design of new mitochondria-targeted molecules.^55^ Apart from demonstrating the ability of TAPYs as mitochondrial vectors, it should be noted that the synthesized dyads can efficiently image the mitochondria of all three cell lines, as shown by the detailed images of the cellular interior provided by these dyes, sometimes even outperforming the well-known mitochondrial dye MTDR. Figure S26 presents selected examples of cells and dyads, with areas of interest highlighted by arrows of different colors. Notably, in some instances, the mitochondrial structure is not clearly visible with the commercial dye, as a blurred stain is observed; however, the same area marked with a **TAPY-BDP** dyad reveals a better resolution. No attempt was made to optimize the concentrations of both types of dyes, as this will be addressed in future research.

Regarding the cellular location of the model compound without the targeting moiety, **prop-BDP**, it showed no preference for mitochondria, as previously mentioned (Figure 3a, last row). Intrigued by the cellular fate of this untargeted compound, a series of complementary measurements were performed. As shown in Figure S27, clear colocalization of **prop-BDP** with Nile Red (NR), a well-known dye for lipid droplets, can be observed.^56^ In contrast, when the same assay was performed with **TAPY­(H)-BDP** and the image merge was done, only a series of green and red dots were observed inside the cells (Figure S27a, bottom), ruling out the localization of the dyad in the lipid bodies.

After confirming that the mitochondria of three cancer cell lines can be efficiently targeted with **TAPY-BDP** dyads, cultures of normal cells were tested for comparison. Endothelial cell line HMEC-1 (human microvascular endothelial cells) commonly used as a model of nontumor cell lines, were selected for this purpose.^57^ As shown in Figure 4, the colocalization pattern of TAPY probes with MTDR in this cell line is completely different compared to the tumor cells. In this case, the two probes do not coincide spatially, regardless of the substituent R in the TAPY dyad. The same assay was performed with human embryonic kidney cells (HEK-293), with similar results (Figure S28).

**Figure 4:**
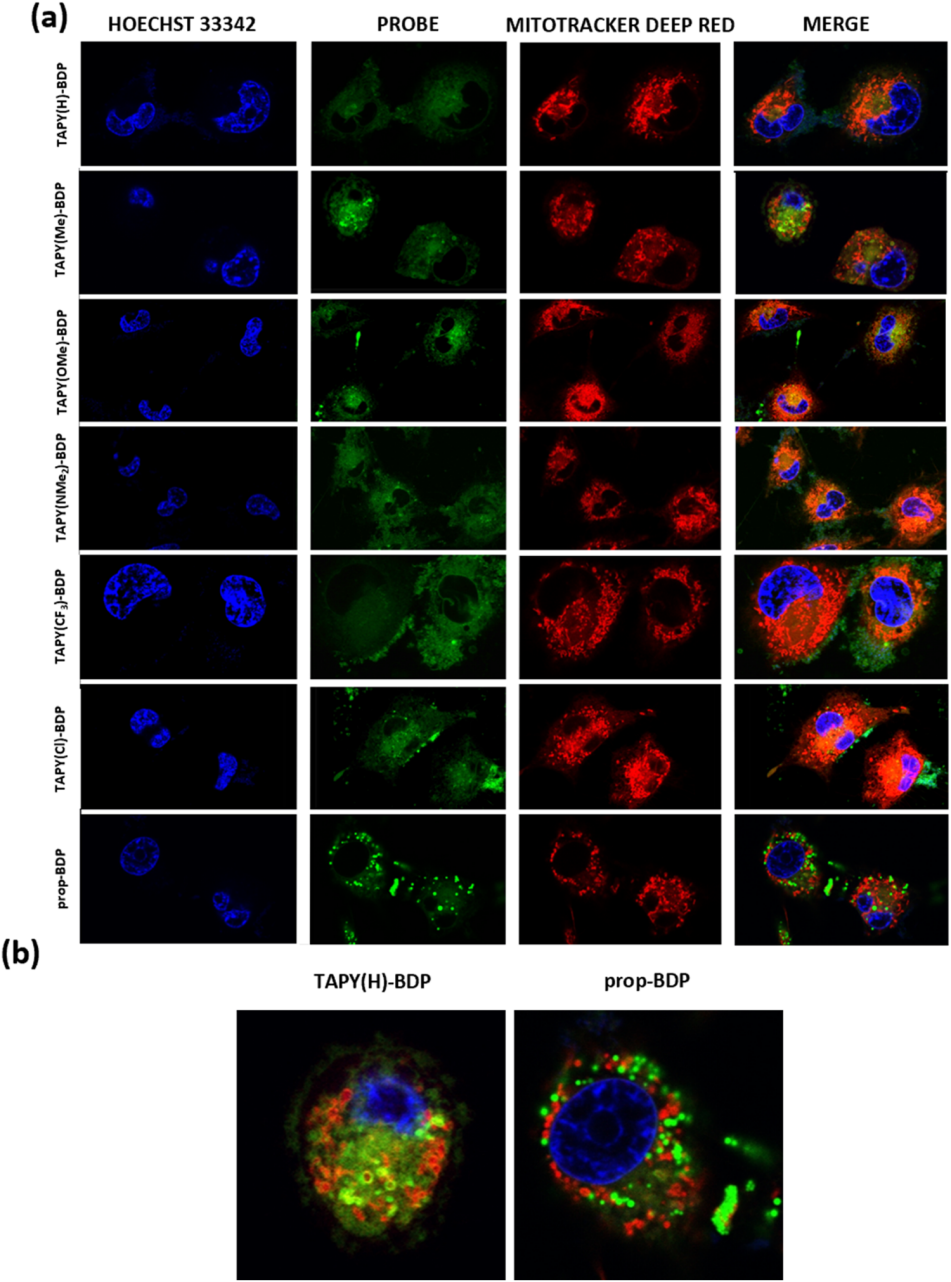
(a) CLSM images of HMEC-1 cells incubated with 0.5 μM of **TAPY-BDP**, and **prop-BDP** probes, 100 nM of Mitotracker Deep Red, and Hoechst 33342 for 30 min at 37 °C. Blue channel: excitation with 405 nm laser (Hoechst 33342); green channel: excitation with 488 nm laser (**TAPY-BDP** dyads and model compound); red channel: excitation with 633 nm laser (Mitotracker Deep Red FM). Also shown the overlay of green and red channels (merge). (b) Zoom of selected images (merge of green and red channels).

Figure 5, which compares the Pearson coefficients calculated for all the probes and cell types tested, illustrates the notable difference of accumulation as a function of the cell type.

**Figure 5:**
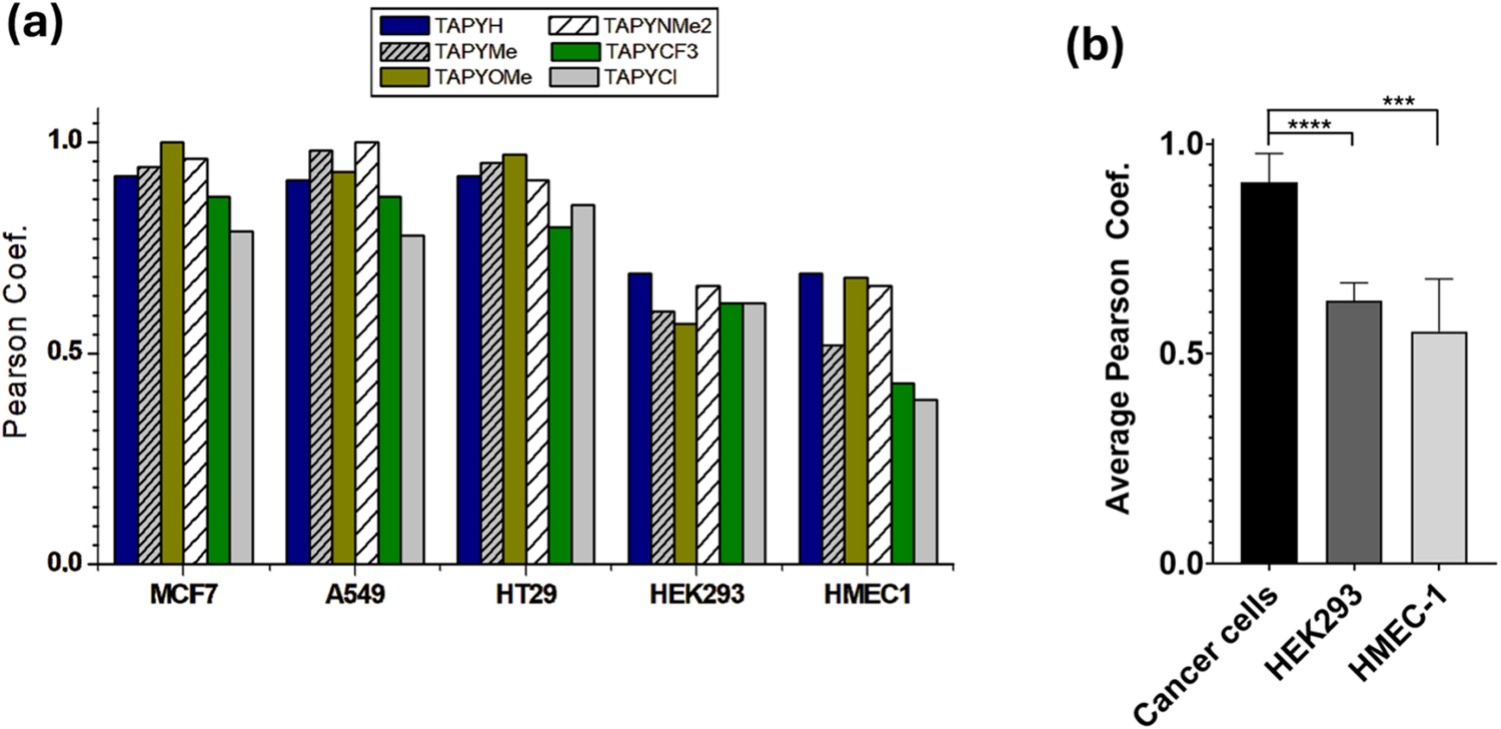
(a) Pearson coefficients for each cell type; (b) Comparative analysis of average Pearson coefficients. The bar plot illustrates the mean cell viability of cancer cells vs HMEC-1 and HEK-293, with error bars representing the standard error of the mean (SEM). Statistical analysis using an unpaired *t* test with Welch’s correction indicates a significant difference between the two groups (*P* < 0.0001 and *P* < 0.0005 denoted by **** and *** respectively).

This differential accumulation of a cationic dye based on the cancerous nature of the cells could be compatible with observations made in the past for Rh123. Davis et al. previously observed preferential accumulation of this dye in cancerous MCF-7 cells compared to normal CV-1 cells (monkey kidney epithelium).^58^ Modica-Napolitano et al. also quantitatively interpreted a similar difference in terms of Nernstian accumulation (driven by the electric potential) in the mitochondria of cancerous versus normal cells, specifically CX-1 (human colon carcinoma cells) compared to normal CV-1 cells.^59^ The dependence of dye accumulation on mitochondrial potential warrants a comprehensive study, involving precise measurement of the potential and its modulation with appropriate uncouplers. However, since the aim of this paper is to introduce TAPY tools as mitochondrial carriers, the role of mitochondrial potential will be investigated and reported separately.

The hydrophilic/hydrophobic balance of the **TAPY-BDP** systems is another factor to consider in order to ascertain the relationships between probe structure and mitochondrial access. To this end, the c log *P* of each molecule was calculated according to the SwissADME protocol, yielding values between 6.17 and 7.20 (see Table 1).^60^ Then, the Pearson coefficient for the colocalization of MTDR with the **TAPY-BDP** dyads was plotted against the c Log *P*. As shown in Figure 6, two distinct sets of data can be identified. The correlation coefficients for HMEC-1 and HEK-293 cells align along a trend separate from that observed for the cancerous MCF-7, A549, and HT-29 cells. It should be noted that the c Log *P* range of our probes is relatively high by common standards, and for c Log *P* values lower than 6, the trend may be reversed (resulting in less efficient mitochondrial targeting). This question remains open for future studies. According to the literature, the relationship between hydrophobicity and cellular localization has been studied for compounds bearing triarylphosphonium cations (such as simple TPP and its analogs with one or more substituents), and it has been found that this parameter significantly influences the efficiency of probe localization.^34–38^ In another study, which focused on Si-rhodamines, Sung et al. systematically examined the mitochondrial localization of probes as a function of dye hydrophobicity,^61^ with log *P* values ranging from 2.29 to 6.33. They found that systems within the 5.50–6.33 range were the most efficient, particularly one compound with a log *P* of 6.00. This suggests the existence of an optimal balance between hydrophilicity and hydrophobicity for mitochondrial accumulation, which may vary depending on the compound family. In our case, this optimal c Log *P* appears to be in the range of 6.2–6.5. Compounds with higher hydrophobicity tend to show poorer results, while more hydrophilic compounds remain to be explored. This aligns with studies by N. L. Oleinick’s group on acridine orange derivatives, which demonstrated that a hexyl chain attached to the quaternized nitrogen atom enhances mitochondrial localization compared to nonyl or hexadecyl chains.^62^ Additionally, Horobin et al. compiled numerous compounds targeting mitochondria and noted that while the majority (69%) have a log *P* between 0 and 5, a notable portion falls outside this range, with 4% having a log *P* > 5 and 27% exhibiting clear hydrophilic characteristics (log *P* < 0).^9^

**Figure 6:**
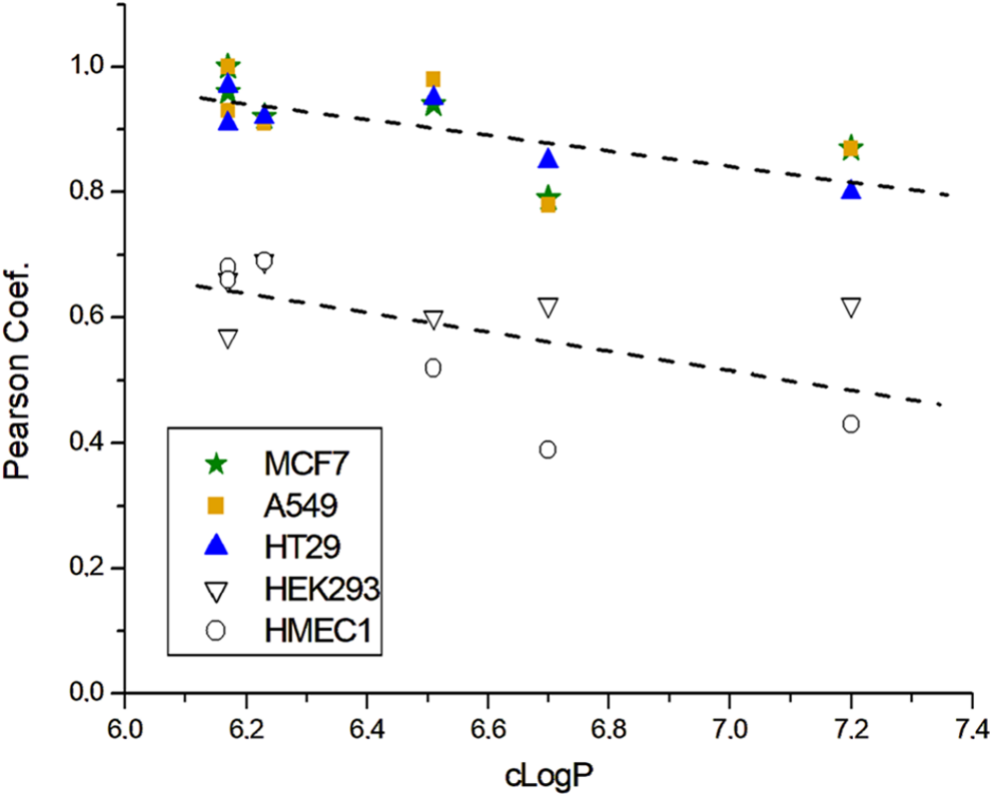
Pearson coefficients vs cLogP.

**Table 1:** c Log *P* Values and Pearson Coefficients

probe	c Log *P*	MCF7	A549	HT29	HMEC-1	HEK293
TAPY(H)-BDP	6.23	0.92	0.91	0.92	0.64	0.69
TAPY(Me)-BDP	6.51	0.94	0.98	0.95	0.52	0.60
TAPY(OMe)-BDP	6.17	1.00	0.93	0.97	0.68	0.57
TAPY(NMe_2_)-BDP	6.17	0.96	1.00	0.91	0.66	0.66
TAPY(CF_3_)-BDP	7.20	0.87	0.87	0.80	0.43	0.62
TAPY(Cl)-BDP	6.70	0.79	0.78	0.85	0.39	0.62

### 2.5 Comparison to a Reference Compound (TPP-Bodipy Derivative)

Up to this point, it has been demonstrated that a standard bodipy fluorophore can be directed to the mitochondria of cancer cells using various **TAPY** carriers. However, it remains to be determined whether this scaffold offers any improvement over the current gold-standard TPP. To address this, the **TPP-BDP** dyad depicted in Figure 7a was synthesized and characterized (see SI for details). A time-lapse live cell imaging experiment with MCF-7 cells was designed to compare this model compound with the **TAPY­(OMe)-BDP** conjugate. Figure 7b shows the evolution of the signal over time, tracking the blue (Hoechst 33258, nuclei) and the green channel (bodipy fluorophore, mitochondria). As shown in Figure 7c, both conjugates follow distinct kinetics for cellular entry: **TPP-BDP** exhibits a faster initial uptake than **TAPY­(OMe)-BDP**. However, around 10 min, the signal from the TAPY derivative surpasses that of the TPP-bearing molecule. By the end of the experiment (30 min), the signal from the TAPY conjugate is approximately twice as strong as that of the TPP compound, as statistically confirmed in Figure 7d.

**Figure 7:**
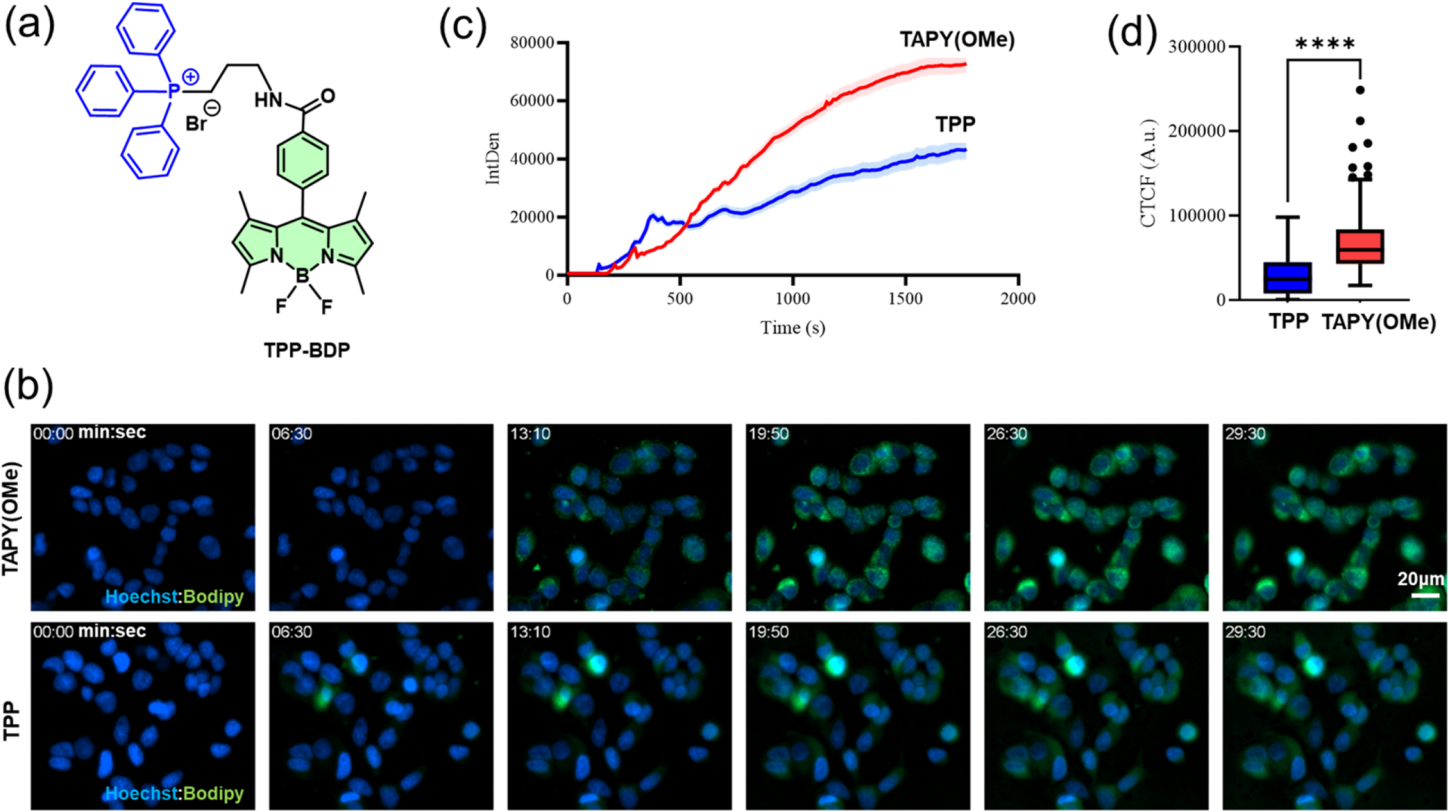
(a) Structure of the reference compound **TPP-BDP**. (b) MCF7 cells were stained with Hoechst 33258 (blue) for 30 min to detect nuclei. Medium was then changed to FluoroBrite DMEM imaging media with no staining. Cells were then placed on top of a Plan-Neofluar 20×/0.50 na objective on a Zeiss Apotome.2 fluorescent microscope. A live cell time-lapse imaging experiment was performed by taking an image every ten seconds; after 2 min of baseline recording, 1 μM of TPP or TAPY dyads were added in the imaging media and signal recorded for up to 30 min. Representative images of one experiment, blue represents nuclei, while green is the signal from bodipy fluorophore; scale bar: 20 μm. (c) Integrated density calculated from images in (b). An area surrounding the mitochondrial signal was draw for several cells in the field of view. Then, ImageJ was used to calculate the integrated density for each time-point. (d) Quantification of the green fluorescence signal (CTCF, corrected total cell fluorescence) of the last time point from experiment in (b). Data are shown as median ± IQR (interquartile range), *n* = 3. Statistical analysis was performed by two-tailed student’s *t* test (**** indicates a *p*-value <0,0001).

With the molecules presented here, we suggest that the TAPY family of carriers could serve as interesting alternatives to the excellent TPP mitochondrial vector. To the best of our knowledge, although other cationic alternatives based on quaternary ammonium salts have been used for mitochondrial targeting,^12,17,21,63–71^ no family of compounds with the synthetic possibilities of TAPYs has been reported.

## 3 Conclusions

Six TAPY-bodipy dyads have been synthesized and characterized, with their intracellular localization definitively shown to be mitochondrial, as demonstrated by colocalization assays using the well-known mitochondrial stain MTDR. A slight dependence on lipophilicity for mitochondrial targeting has been observed, with molecules having a c Log *P* around 6.2 showing the best Pearson colocalization indexes. Importantly, when comparing cancerous cells (MCF-7, A549, and HT-29) with HMEC-1 and HEK-293, clear targeting of the mitochondria only in the former group was observed (colocalization indexes around 0.9–1.0). Notably, a quantitative comparison between one of the TAPY-bodipy conjugates and a model TPP-bodipy dyad revealed that mitochondrial accumulation of the TAPY conjugate in MCF-7 cells was twice that of the TPP derivative. This finding opens the door for the future synthesis of TAPY-drug conjugates, aimed at the preferential accumulation of drugs in the mitochondria of dysfunctional cells. One feature that must be emphasized at this point is that the synthetic possibilities offered by this approach are far broader than those available for TPP and other cationic vectors, given the extensive diversity of TAPYs that can be prepared, including carriers with their own optical capabilities (absorption, fluorescence, photoreactivity). The potential of TAPYs is enormous, and they are expected to be finely tuned to serve as alternatives or complements, in certain applications, to the widely used TPP.

## 4 Materials and Methods

See Supporting Information file.

## Supplementary Material



## Notes

1H/13C NMR, HRMS, absorption and emission spectra, MTT assays, and complementary CLSM images and analysis (PDF)
