# Supplementary material for: Introducing TAPY as a Versatile Alternative to TPP for Selective Mitochondrial Targeting in Cancer Cells
Source: Bioconjug Chem. 2025 Mar 31;36(4):697–706. doi: 10.1021/acs.bioconjchem.4c00554 (PMC12129258; doi:10.1021/acs.bioconjchem.4c00554)
Supplement: Supplementary file 1 [file bc4c00554_si_001.pdf]

Introducing TAPY as a Versatile Alternative to TPP for Selective Mitochondrial Targeting in Cancer Cells

Jean C. Neto,[a] Federico Lucantoni,[b] Leydy V. González, [a] Eva Falomir,[a]
Juan F. Miravet,[a] Francisco Galindo*[a]

[a] *Universitat Jaume I de Castellón, Departamento de Química Inorgánica y Orgánica, Avda. Vicente Sos Baynat s/n, 12071 Castellón de la Plana, Spain.*
[b] *Laboratory of Cellular Stress and Cell Death Pathways, Centro de Investigación Príncipe Felipe (CIPF), Valencia, Spain.*

* *E-mail: francisco.galindo@uji.es*

Table of Contents

Reagents and Instruments......4
Scheme S 1. 4
Synthesis of the pyrylium salts (1a-f). 4
Synthesis of the nucleophilic TAPY intermediates (2a-f). 6
Synthesis of the electrophilic fluorophore, borodipyrromethene (BDP-COOH). 7
Synthesis of TAPY-BDP dyads. 9
Synthesis of the model compound, prop-BDP.......11
Synthesis of the control compound, TPP-BDP. 12
Optical measurements. 12
Toxicity assays. 13
Confocal Microscopy.......13
Live cell time-lapse imaging......14
Figure S 1. ^{1}H NMR, ^{13}C NMR (CD_3CN) spectra of compound **1a**. 15
Figure S 2. ^{1}H NMR, ^{13}C NMR (DMSO-d_6) spectra of compound **1b**. 16
Figure S 3. ^{1}H NMR, ^{13}C NMR (DMSO-d_6) spectra of compound **1c**......17
Figure S 4. ^{1}H NMR, ^{13}C NMR (DMSO-d_6) spectra of compound **1d**. 18
Figure S 5. ^{1}H NMR, ^{13}C NMR (DMSO-d_6) spectra of compound **1e**......19
Figure S 6. ^{1}H NMR, ^{13}C NMR (CD_3CN) spectra of compound **1f**......20
Figure S 7. ^{1}H NMR, ^{13}C NMR (CD_3CN) and Mass spectra of compound **2a.** 21
Figure S 8. ^{1}H NMR, ^{13}C NMR (DMSO-d_6) and Mass spectra of compound **2b.** 22
Figure S 9. ^{1}H NMR, ^{13}C NMR (DMSO-d_6) and mass spectra of compound **2c.** 23
Figure S 10. ^{1}H NMR, ^{13}C NMR (DMSO-d_6) and mass spectra of compound **2d**......24
Figure S 11. ^{1}H NMR, ^{13}C NMR (DMSO-d_6) and mass spectra of compound **2e**. 25
Figure S 12. ^{1}H NMR, ^{13}C NMR (DMSO-d_6) and mass spectra of compound **2f.** 26
Figure S 13. ^{1}H NMR, ^{13}C NMR (DMSO-d_6) and mass spectra of compound **BDP-COOH.** 27
Figure S 14. ^{1}H NMR, ^{13}C NMR (DMSO-d_6) and mass spectra of compound **TAPY(H)-BDP.**......28
Figure S 15. ^{1}H NMR, ^{13}C NMR (DMSO-d_6) and mass spectra of compound **TAPY(Me)-BDP.** 29
Figure S 16. ^{1}H NMR, ^{13}C NMR (DMSO-d_6) and mass spectra of compound **TAPY(OMe)-BDP.** 30
Figure S 17. ^{1}H NMR, ^{13}C NMR (DMSO-d_6) and mass spectra of compound **TAPY(NMe_2)-BDP.** 31
Figure S 18. ^{1}H NMR, ^{13}C NMR (DMSO-d_6) and mass spectra of compound **TAPY(CF_3)-BDP.** 32
Figure S 19. ^{1}H NMR, ^{13}C NMR (DMSO-d_6) and mass spectra of compound **TAPY(Cl)-BDP.**......33
Figure S 20 ^{1}H NMR, ^{13}C NMR (DMSO-d_6) and mass spectra of compound **prop-BDP.**......34
Figure S 21. ^{1}H NMR, ^{13}C NMR (DMSO-d_6) and mass spectra of compound **TPP-BDP**. 35
Figure S 22 Absorption and fluorescence emission spectra of the TAPY-BDP, prop-BDP and TPP-BDP......36
Figure S 23 MTT cell viability assays......37
Figure S 24 CLSM images of A549 cells......38

Figure S 25 CLSM images of HT-29 cells 39
Figure S 26 Selected CLSM images of MCF7, A549 and HT-29 cells 40
Figure S 27. CLSM images. HT-29 cells incubated with TAPY(H)-BDP, prop-BDP, Nile Red 41
Figure S 28. CLSM images of HEK293 cells 42
Figure S 29 Colocalization analysis of specific probes in MCF7 cells 43
Figure S 30 Colocalization analysis of specific probes in A549 cells 44
Figure S 31 Colocalization analysis of specific probes in HT-29 cells 45
Figure S 32 Colocalization analysis of specific probes in MCF7 cells with Nile Red. 46
Supplemental references 48

Reagents and Instruments. All commercially available reagents and solvents were used without further purification. All solvents were spectroscopic grade. ^{1}H and ^{13}C NMR spectra were recorded on a Bruker Avance III HD spectrometer, operating at 400 MHz for ^{1}H and 101 MHz for ^{13}C. In some cases, ^{1}H spectra were recorded at 300 MHz and ^{13}C spectra at 75 MHz on a Bruker Avance III HD spectrometer. High-resolution mass spectra (HRMS) were acquired using a Waters Q-Tof Premier mass spectrometer equipped with an electrospray ionization (ESI) source. UV-vis absorption spectra were measured using a Cary 60 UV-vis spectrophotometer, with 1 cm path length quartz cuvettes and a 3 mL sample volume. Cellular images were captured on a Leica TCS SP8 inverted confocal laser-scanning microscope, and image analysis was performed using ImageJ software.

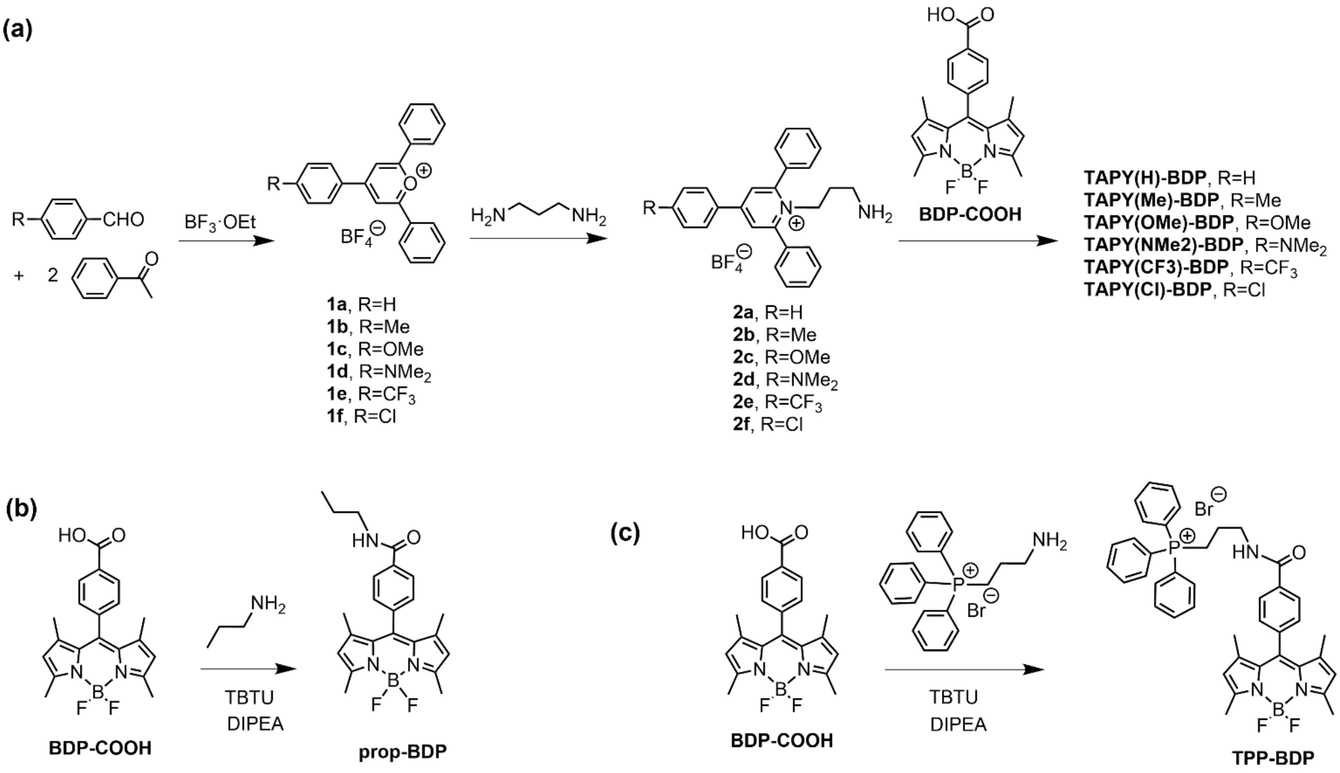

Scheme S 1. Synthetic routes

Synthesis of the pyrylium salts (1a-f). The synthesis of the pyrylium salts (Py$^+$) precursors was carried out according to the following procedure: [1] For the synthesis of the TAPY precursors **1a-f**, one equivalent of *p*-substituted benzaldehyde and two equivalents of acetophenone were used, along with 2.4 equivalents of boron trifluoride etherate (BF_3.OEt_2). The reactions were performed under reflux in toluene for 24 hours under an inert atmosphere. After this time, the reaction mixture was cooled to room temperature

and poured into approximately 200 mL of diethyl ether. The resulting precipitate was recovered by filtration, washed with diethyl ether, and dried under vacuum. This process led to the formation of the corresponding triarylpyrylium salts.

1a: Starting materials: 1.42 g of benzaldehyde, 3.22 g of acetophenone. After precipitation: yellow solid, 2.50 g, yield of 47%. ^{1}H NMR (400 MHz, CD_3CN) δ 8.72 (s, 2H), 8.43 (d, *J* = 7.2 Hz, 4H), 8.31 (d, *J* = 7.2 Hz, 2H), 7.92 – 7.82 (m, 3H), 7.86 – 7.72 (m, 6H) ppm. ^{13}C NMR (101 MHz, CD_3CN) δ 171.94, 167.46, 136.39, 136.26, 133.88, 131.05, 131.04, 130.59, 129.99, 129.72, 116.78 ppm.

1b: Starting materials: 1.07 g of 4-methylbenzaldehyde, 2.14 g of acetophenone. After precipitation: yellow solid, 1.90 g, yield of 52%. ^{1}H NMR (400 MHz, DMSO-d_6) δ 9.12 (s, 2H), 8.57 (t, *J* = 8.2 Hz, 3H), 7.87 (t, *J* = 7.3 Hz, 1H), 7.79 (t, *J* = 7.5 Hz, 2H), 7.61 (d, *J* = 7.8 Hz, 1H), 2.52 (s, 3H) ppm. ^{13}C NMR (101 MHz, DMSO-d_6) δ 169.70, 164.65, 146.95, 134.85, 130.54, 130.16, 129.80, 129.55, 129.16, 128.68, 114.36, 21.39 ppm.

1c: Starting materials: 1.00 g of 4-methoxybenzaldehyde, 1.75 g of acetophenone. After precipitation: yellow solid, 1.80 g, yield of 58%. ^{1}H NMR (400 MHz, DMSO-d_6) δ 9.04 (s, 2H), 8.70 (d, *J* = 9.0 Hz, 2H), 8.55 (d, *J* = 7.1 Hz, 4H), 7.85 (t, *J* = 7.3 Hz, 2H), 7.78 (t, *J* = 7.4 Hz, 4H), 7.32 (d, *J* = 9.0 Hz, 2H), 4.00 (s, 3H) ppm. ^{13}C NMR (101 MHz, DMSO-d_6) δ 168.87, 165.90, 163.62, 134.60, 133.04, 129.74, 129.28, 128.48, 124.37, 115.60, 113.25, 56.24 ppm.

1d: Starting materials: 1.40 g of 4-(dimethylamino)benzaldehyde, 2.30 g of acetophenone. After precipitation: yellow solid, 2.30 g, yield of 55%. ^{1}H NMR (300 MHz, DMSO-d_6) δ 8.66 (s, 2H), 8.57 (d, *J* = 9.5 Hz, 2H), 8.42 (d, *J* = 6.5 Hz, 4H), 7.83 – 7.64 (m, 6H), 6.99 (d, *J* = 9.4 Hz, 2H), 3.26 (s, 6H) ppm. ^{13}C NMR (75 MHz, DMSO-d_6) δ 165.09, 159.03, 155.95, 133.60, 133.49, 129.85, 129.54, 127.63, 118.24, 113.09, 109.51, 30.69 ppm.

1e: Starting materials: 1.50 g of 4-(trifluoromethyl)benzaldehyde, 2.10 g of acetophenone. After precipitation: yellow solid, 1.70 g, yield of 42%. ^{1}H NMR (400 MHz, DMSO-d_6) δ 9.25 (s, 2H), 8.75 (d, *J* = 8.3 Hz, 2H), 8.62 (d, *J* = 7.2 Hz, 4H), 8.17 (d, *J* = 8.6 Hz, 2H), 7.90 (t, *J* = 7.3 Hz, 2H), 7.82 (t, *J* =

7.6 Hz, 4H) ppm. ^{13}C NMR (101 MHz, DMSO-d_6) δ 170.74, 163.71, 136.53, 135.31, 130.70, 129.93, 128.98, 128.34, 128.07, 127.86, 126.49, 116.33 ppm.

1f: Starting materials: 1.12 g of 4-chlorobenzaldehyde, 1.94 g of acetophenone. After precipitation: yellow solid, 1.70 g, yield of 49%. ^{1}H NMR (400 MHz, CD_3CN) δ 8.69 (s, 2H), 8.42 (d, J = 7.3 Hz, 4H), 8.29 (d, J = 8.9 Hz, 2H), 7.91 – 7.84 (m, 4H), 7.81 – 7.75 (m, 4H) ppm. ^{13}C NMR (101 MHz, CD_3CN) δ 172.09, 166.04, 142.39, 136.50, 132.52, 132.17, 131.23, 131.07, 129.94, 129.76, 116.72 ppm.

Synthesis of the nucleophilic TAPY intermediates (2a-f). The synthesis of nucleophilic TAPYs (2a-f) was carried out according to the following synthetic procedure: [2] The synthesis involved the reaction of 1,3-diaminopropane (1.1 equivalents) with the previously synthesized compounds **1a-f** (1 equivalent) in absolute ethanol under an inert nitrogen atmosphere, followed by reflux for 24 hours. After the reaction, the mixture was removed from reflux, and the excess ethanol was evaporated using a rotary evaporator. The product was then precipitated in diethyl ether (200 mL). The resulting solid was filtered, repeatedly washed with ether, and dried under vacuum. This process resulted in the formation of the corresponding triarylpyridinium salts **2a-f**.

2a: Starting materials: 2.50 g of 1a, 0.51 g of 1,3-diaminopropane. Result after precipitation: Pink solid, 1.80 g, yield 63%. ^{1}H NMR (400 MHz, MeOD) δ 8.29 (s, 2H), 8.04 (dd, J = 8.1, 1.7 Hz, 2H), 7.83 – 7.77 (m, 4H), 7.73 – 7.68 (m, 6H), 7.65 – 7.57 (m, 3H), 4.52 – 4.43 (m, 2H), 2.12 (t, J = 7.2 Hz, 2H), 1.74 – 1.62 (m, 2H) ppm. ^{13}C NMR (101 MHz, MeOD) δ 158.07, 157.26, 135.09, 134.36, 133.47, 132.34, 130.90, 130.54, 130.27, 129.45, 127.83, 54.11, 39.23, 33.37 ppm. (ESI-TOF)+ calculated for $C_{26}H_{25}N_2^+$ (M^+) (m/z): 365.2012; experimental (M^+) (m/z): 365.2024.

2b: Starting materials: 1.36 g of 1b, 0.27 g of 1,3-diaminopropane. Result after precipitation: Pink solid, 0.90 g, yield 58%. ^{1}H NMR (300 MHz, DMSO-d_6) δ 8.41 (s, 2H), 8.17 (d, J = 8.4 Hz, 2H), 7.89 – 7.78 (m, 4H), 7.74 – 7.66 (m, 6H), 7.42 (t, J = 8.3 Hz, 2H), 4.37 (t, J = 7.8 Hz, 2H), 2.41 (s, 3H), 2.00 (t, J = 6.5 Hz, 2H), 1.49 – 1.34 (m, 2H) ppm. ^{13}C NMR (75 MHz, DMSO-d_6) δ 155.97, 153.78, 142.92, 133.15, 130.79, 130.21, 129.22, 128.99, 128.57, 125.44, 53.11, 38.38, 32.43, 20.99 ppm. (ESI-TOF)+ calculated for $C_{27}H_{27}N_2^+$ (M^+) (m/z): 379.2169; experimental (M^+) (m/z): 379.2173.

2c: Starting materials: 0.86 g of 1c, 0.16 g of 1,3-diaminopropane. Result after precipitation: Pink solid, 0.63 g, yield 65%. ^{1}H NMR (300 MHz, DMSO-*d6*) δ 8.38 (s, 2H), 8.28 (d, *J* = 8.8 Hz, 2H), 7.88 – 7.77 (m, 4H), 7.76 – 7.65 (m, 6H), 7.14 (d, *J* = 8.4 Hz, 2H), 4.34 (t, 2H), 3.88 (s, 3H), 2.00 (t, *J* = 6.5 Hz, 2H), 1.42 (t, *J* = 7.7 Hz, 2H) ppm. ^{13}C NMR (75 MHz, DMSO-*d6*) δ 162.93, 155.74, 153.34, 133.22, 130.75, 130.66, 129.22, 128.99, 125.00, 124.67, 115.07, 55.69, 52.78, 38.22, 32.12 ppm. (ESI-TOF)+ calculated for $C_{27}H_{27}N_2O^+$ (M^+) (m/z): 395.2118; experimental (M^+) (m/z): 395.2128.

2d: Starting materials: 1.95 g of 1d, 0.36 g of 1,3-diaminopropane. Result after precipitation: Pink solid, 1.35 g, yield 62%. ^{1}H NMR (400 MHz, DMSO-*d6*) δ 8.20 (s, 2H), 8.15 (d, *J* = 9.3 Hz, 2H), 7.83 – 7.78 (m, 4H), 7.67 (t, 6H), 6.81 (d, *J* = 9.3 Hz, 2H), 4.27 – 4.19 (m, 2H), 3.07 (s, 6H), 1.99 (t, *J* = 6.7 Hz, 2H), 1.44 – 1.33 (m, 2H) ppm. ^{13}C NMR (101 MHz, DMSO-*d6*) δ 154.97, 153.15, 133.48, 130.54, 130.23, 129.18, 128.92, 122.38, 118.36, 112.04, 52.03, 39.60, 38.20, 35.75, 32.26 ppm. (ESI-TOF)+ calculated for $C_{28}H_{30}N_3^+$ (M^+) (m/z): 408.2434; experimental (M^+) (m/z): 408.2446.

2e: Starting materials: 1.40 g of 1e, 0.24 g of 1,3-diaminopropane. Result after precipitation: Grey solid, 0.85 g, yield 55%. ^{1}H NMR (300 MHz, DMSO-*d6*) δ 8.55 (s, 2H), 8.46 (d, *J* = 8.3 Hz, 2H), 7.98 (d, *J* = 8.3 Hz, 2H), 7.90 – 7.81 (m, 4H), 7.76 – 7.68 (m, 6H), 4.42 (t, *J* = 7.8 Hz, 2H), 2.07 (t, *J* = 7.0 Hz, 2H), 1.54 (t, *J* = 7.7 Hz, 2H) ppm. ^{13}C NMR (75 MHz, DMSO-*d6*) δ 156.33, 152.51, 137.29, 132.86, 130.98, 129.64, 129.19, 129.09, 127.55, 126.94, 126.28, 126.23, 52.98, 37.48, 30.33 ppm. (ESI-TOF)+ calculated for $C_{27}H_{24}F_3N_2^+$ (M^+) (m/z): 433.1886; experimental (M^+) (m/z): 433.1893.

2f: Starting materials: 2.85 g of 1f, 0.54 g of 1,3-diaminopropane. Result after precipitation: Grey solid, 1.65 g, yield 51%. ^{1}H NMR (300 MHz, DMSO-*d6*) δ 8.48 (s, 2H), 8.31 (d, *J* = 8.4 Hz, 2H), 7.91 – 7.80 (m, 4H), 7.76 – 7.65 (m, 9H), 4.42 (t, *J* = 7.7 Hz, 2H), 2.02 (t, *J* = 6.6 Hz, 2H), 1.49 – 1.39 (m, 2H) ppm. ^{13}C NMR (75 MHz, DMSO DMSO-*d6*) δ 156.36, 152.54, 137.31, 132.89, 131.00, 129.66, 129.22, 129.11, 127.57, 126.97, 126.30, 53.01, 37.50, 30.35 ppm. (ESI-TOF)$^+$ calculado para $C_{26}H_{24}ClN_2^+$ (M+) (m/z): 399,1623; experimental (M+) (m/z): 399,1625.

Synthesis of the electrophilic fluorophore, borodipyrromethene (BDP-COOH). The synthesis of an electrophilic borodipyrromethene (BODIPY) derivative was carried out according to the following

synthetic procedure:[3] First, the methyl esther of **BDP-COOH** was prepared (**BDP-COOMe**). For the synthesis of **BDP-COOMe**, methyl-4-formylbenzoate (1.0 equivalent, 1030 mg) and 2,4-dimethylpyrrole (2.2 equivalents, 1311 mg) were added to anhydrous THF (80 mL) under an inert N_2 atmosphere. After stirring for 20 minutes, 10 drops of trifluoroacetic acid (TFA) were slowly added. The reaction mixture was left to stir at room temperature for 24 hours. Subsequently, 2,3-dichloro-5,6-dicyano-p-benzoquinone (DDQ, 1.0 equivalent, 1420 mg) dissolved in THF (5 mL) was added and stirred for an additional 4 hours. The reaction mixture was cooled in an ice bath before adding triethylamine (20 equivalents, 20 mL) and $BF_3 \cdot OEt_2$ (24 equivalents, 40 mL). The solution was stirred at room temperature under a nitrogen atmosphere overnight. Once the reaction period was complete, the mixture was transferred to a separatory funnel, 50 mL of 5% bicarbonate solution and 50 mL of ethyl acetate were added, and the organic phase was collected. This process was repeated 4 times. The combined organic phases were dried with anhydrous sodium sulfate and evaporated using a rotary evaporator. The crude material was dissolved in dichloromethane (DCM, 20 mL), filtered through Celite, and finally purified using a silica gel chromatographic column with DCM as the eluent to obtain the **BDP-COOMe** compound. Following the isolation of **BDP-COOMe**, the methoxy ($-OCH_3$) group was removed to yield the acid fluorophore, boron-dipyrromethene (**BDP-COOH**). THF (20 mL) was used as the solvent, and anhydrous lithium hydroxide (60 equivalents) was added in distilled water (20 mL) with constant stirring at room temperature under an inert N_2 atmosphere. The reaction was monitored by TLC using an EtOAc 80:20 eluent mixture. When the reaction was complete, the mixture was concentrated on a rotary evaporator to remove the excess solvent. The pH of the solution was adjusted to pH 3, and 3 extractions with EtOAc were performed. The combined organic phases were dried with anhydrous sodium sulfate (Na_2SO_4) and evaporated using a rotary evaporator to obtain the boron-dipyrromethene acid compound (**BDP-COOH**).

BDP-COOH: Starting materials: 1.03 g of methyl-4-formylbenzoate, 1.31 g of 2,4-dimethylpyrrole. Result after purification: Red solid, 0.78 g, 65% yield. ^{1}H NMR (400 MHz, DMSO-d_6) δ 8.10 (d, J = 8.3 Hz, 2H), 7.53 (d, J = 8.3 Hz, 2H), 6.19 (s, 2H), 2.46 (s, 6H), 1.33 (s, 6H) ppm. ^{13}C NMR (101 MHz, DMSO-d_6) δ 166.78, 155.24, 142.60, 140.75, 138.38, 131.49, 130.25, 130.10, 128.38, 121.56, 14.19,

14.01 ppm. (ESI-TOF)- calculated for $C_{20}H_{19}BF_2N_2O_2$ (M^-) (m/z): 367.1435; experimental (M^-) (m/z): 367.1466.

Synthesis of TAPY-BDP dyads. The synthesis of TAPY-BDP dyads was carried out according to the following synthetic procedure:[3] For the synthesis of the dyads TAPY-BDP's compounds, the process began with the coupling of 1 equivalent of the acidic fluorophore **BDP-COOH**, dissolved in DMF at room temperature under a nitrogen atmosphere. N,N-diisopropylethylamine (DIPEA, 1.1 equivalents) and the coupling agent TBTU (1.1 equivalents) were added. The mixture was stirred for 30 minutes, and then 1 equivalent of the respective nucleophiles 2a-f previously synthesized was added under constant stirring, at room temperature, and under an inert nitrogen atmosphere. After 24 hours of reaction, the solution was concentrated using a rotary evaporator to remove the DMF solvent. The residue was then dissolved in DCM, quenched with water, and washed twice with slightly acidic water (pH 2-3). The organic phases were dried over anhydrous Na_2SO_4, filtered, and concentrated using a rotary evaporator. Finally, the solid obtained was dried in a vacuum oven at 45°C for 24 hours.

TAPY(H)-BDP: Starting materials: 0.30 g of TAPY(H), 0.25 g of BDP-COOH. The result after purification was a red solid, yielding 0.22 g with a 42% yield. ^{1}H NMR (300 MHz, DMSO-*d₆*) δ 8.46 (s, 2H), 8.33 (t, *J* = 5.3 Hz, 1H), 8.25 (d, *J* = 6.5 Hz, 2H), 7.85 (dd, *J* = 7.9, 1.7 Hz, 4H), 7.75 (d, *J* = 8.4 Hz, 2H), 7.68 – 7.56 (m, 9H), 7.49 (d, *J* = 8.3 Hz, 2H), 6.23 (s, 2H), 4.48 – 4.37 (m, 2H), 2.82 (d, *J* = 6.1 Hz, 2H), 2.48 (s, 6H), 1.77 – 1.60 (m, 2H), 1.39 (s, 6H) ppm. ^{13}C NMR (75 MHz, DMSO-d_6) δ 165.32, 155.98, 155.22, 154.12, 142.64, 141.08, 136.85, 134.42, 133.14, 132.89, 132.38, 130.82, 130.44, 129.60, 129.10, 128.95, 128.67, 128.05, 127.89, 126.09, 53.07, 35.89, 29.76, 14.26, 14.10 ppm. (ESI-TOF)+ calculated for $C_{46}H_{42}BF_2N_4O^+$ (M^+) (m/z): 715.3414; experimental (M^+) (m/z): 715.3430.

TAPY(Me)-BDP: Starting materials: 0.38 g of TAPY(Me), 0.31 g of BDP-COOH. The result after purification was a red solid, yielding 0.32 g with a 48% yield. ^{1}H NMR (300 MHz, DMSO-d_6) δ 8.43 (s, 2H), 8.32 (t, *J* = 5.4 Hz, 1H), 8.18 (d, *J* = 8.2 Hz, 2H), 7.89 – 7.79 (m, 4H), 7.74 (d, *J* = 8.2 Hz, 2H), 7.68 – 7.54 (m, 6H), 7.49 (d, *J* = 8.0 Hz, 2H), 7.42 (d, *J* = 8.1 Hz, 2H), 6.23 (s, 2H), 4.47 – 4.31 (m, 2H), 2.88 – 2.75 (m, 2H), 2.48 (s, 6H), 2.41 (s, 3H), 1.68 (s, 2H), 1.39 (s, 6H) ppm. ^{13}C NMR (75 MHz, DMSO-d_6)

δ 165.32, 155.86, 155.21, 153.93, 143.02, 142.64, 141.08, 136.84, 134.42, 132.94, 130.77, 130.44, 130.26, 130.18, 129.08, 128.93, 128.60, 128.04, 127.88, 125.50, 121.56, 52.92, 35.89, 29.77, 21.02, 14.10 ppm. (ESI-TOF)+ calculated for $C_{47}H_{44}BF_2N_4O^+$ (M^+) (m/z): 729.3571; experimental (M^+) (m/z): 729.3593.

TAPY(OMe)-BDP: Starting materials: 0.31 g of TAPY(OMe), 0.25 g of BDP-COOH. The result after purification was a red solid, yielding 0.28 g with a 52% yield. ^{1}H NMR (300 MHz, DMSO-*d6*) δ 8.39 (s, 2H), 8.37 – 8.23 (m, 4H), 7.88 – 7.80 (m, 4H), 7.74 (d, *J* = 8.3 Hz, 2H), 7.64 – 7.46 (m, 10H), 7.14 (d, *J* = 9.1 Hz, 2H), 6.23 (s, 2H), 4.35 (t, *J* = 8.3 Hz, 2H), 3.88 (s, 3H), 2.89 – 2.74 (m, 2H), 2.48 (s, 6H), 1.75 – 1.59 (m, 2H), 1.39 (s, 6H) ppm. ^{13}C NMR (75 MHz, DMSO-*d6*) δ 165.31, 162.98, 155.63, 155.22, 153.46, 142.63, 141.08, 136.84, 134.42, 133.03, 130.70, 130.43, 129.10, 128.92, 128.04, 127.88, 124.98, 124.72, 121.56, 115.10, 55.71, 52.67, 35.88, 29.75, 14.26, 14.10 ppm. (ESI-TOF)+ calculated for $C_{47}H_{44}BF_2N_4O_2^+$ (M^+) (m/z): 745.3520; experimental (M^+) (m/z): 745.3536.

TAPY(NMe2)-BDP: Starting materials: 0.24 g of TAPY(NMe_2), 0.18 g of BDP-COOH. The result after purification was a red solid, yielding 0.200 g with a 48% yield. ^{1}H NMR (300 MHz, DMSO-*d6*) δ 8.44 (s, 2H), 8.31 (t, 1H), 8.18 (d, *J* = 8.0 Hz, 2H), 7.90 – 7.80 (m, 4H), 7.74 (d, *J* = 8.0 Hz, 2H), 7.67 – 7.55 (m, 6H), 7.50 (d, *J* = 7.9 Hz, 2H), 7.43 (d, *J* = 8.0 Hz, 2H), 6.23 (s, 2H), 4.37 (t, 2H), 2.88 – 2.75 (m, 2H), 2.48 (s, 6H), 2.42 (s, 6H), 1.68 (s, 2H), 1.39 (s, 6H) ppm. ^{13}C NMR (75 MHz, DMSO-*d6*) δ 165.28, 155.84, 155.19, 153.91, 143.00, 142.60, 141.06, 136.82, 134.40, 132.93, 130.75, 130.42, 130.23, 130.16, 129.06, 128.91, 128.59, 128.01, 127.87, 125.48, 121.53, 52.92, 29.75, 21.00, 14.23, 14.08 ppm.(ESI-TOF)+ calculated for $C_{48}H_{47}BF_2N_5O^+$ (M^+) (m/z): 758.3836; experimental (M^+) (m/z): 758.3854.

TAPY(CF3)-BDP: Starting materials: 0.25 g of TAPY(CF_3), 0.19 g of BDP-COOH. The result after purification was a red solid, yielding 0.24 g with a 56% yield. ^{1}H NMR (300 MHz, DMSO-*d6*) δ 8.56 (s, 2H), 8.46 (d, *J* = 8.4 Hz, 3H), 8.32 (t, *J* = 5.4 Hz, 1H), 7.98 (d, *J* = 8.1 Hz, 2H), 7.85 (d, *J* = 5.8 Hz, 4H), 7.75 (d, *J* = 8.0 Hz, 3H), 7.70 – 7.56 (m, 6H), 7.51 (t, *J* = 7.1 Hz, 3H), 6.23 (s, 2H), 4.45 (t, *J* = 8.2 Hz, 2H), 2.83 (q, *J* = 5.9 Hz, 2H), 2.48 (s, 6H), 1.71 (s, 2H), 1.39 (s, 6H) ppm. ^{13}C NMR (75 MHz, DMSO-*d6*) δ 165.33, 156.26, 155.21, 152.56, 142.63, 141.07, 136.86, 134.40, 132.75, 130.92, 130.44, 129.65,

129.09, 128.98, 128.03, 127.90, 126.96, 126.30, 121.56, 53.37, 35.89, 29.75, 14.25, 14.10 ppm. (ESI-TOF)+ calculated for $C_{47}H_{41}BF_5N_4O^+$ (M^+) (m/z): 783.3288; experimental (M^+) (m/z): 783.3301.

TAPY(Cl)-BDP: Starting materials: 0.34 g of TAPY(Cl), 0.27 g of BDP-COOH. The result after purification was a red solid, yielding 0.30 g with a 50% yield. ^{1}H NMR (300 MHz, DMSO-d_6) δ 8.49 (s, 2H), 8.30 (d, J = 8.7 Hz, 3H), 7.84 (d, J = 5.8 Hz, 4H), 7.78 – 7.66 (m, 5H), 7.65 – 7.54 (m, 6H), 7.49 (d, J = 8.3 Hz, 2H), 6.23 (s, 2H), 4.40 (d, J = 8.0 Hz, 2H), 2.82 (d, J = 6.0 Hz, 2H), 2.48 (s, 6H), 1.68 (s, 2H), 1.39 (s, 6H) ppm. ^{13}C NMR (75 MHz, DMSO-d_6) δ 169.54, 156.06, 155.22, 142.63, 137.52, 132.82, 130.85, 130.55, 129.61, 129.09, 128.95, 128.04, 127.89, 126.14, 121.56, 53.15, 35.82, 29.76, 14.25, 14.10 ppm. (ESI-TOF)+ calculated for $C_{46}H_{41}BClF_2N_4O^+$ (M^+) (m/z): 749.3025; experimental (M^+) (m/z): 749.3032.

Synthesis of the model compound, prop-BDP. The synthesis was performed using the previously synthesized **BDP-COOH** acid fluorophore (1 equivalent) in 20 mL of chloroform ($CHCl_3$) as the solvent. DIPEA (1.01 equivalents) and TBTU (1.01 equivalents) were added to the mixture, which was stirred for 30 minutes. Subsequently, 1.01 equivalents of *n*-propylamine to obtain prop-BDP, was added under constant stirring at room temperature in an inert nitrogen (N_2) atmosphere. After 24 hours of reaction, 30 mL of $CHCl_3$ were added, and the reaction mixture was washed twice with 1.0 M HCl (30 mL), followed by two washes with saturated sodium bicarbonate solution ($NaHCO_3$, 30 mL) and a wash with distilled water (20 mL). The organic phases were then dried over anhydrous Na_2SO_4, filtered, and concentrated using a rotary evaporator. The purification was carried out by column chromatography using a hexane:acetate (EtOAc) elution system in ratios of 7:3 and 1:1. Finally, the resulting purple solid was dried in a vacuum oven at 45°C for 24 hours.

prop-BDP: Starting materials: 0.06 mL of *n*-propilamine, 0.25 g of **BDP-COOH**. Result after precipitation: Purple solid, 0.14 g, 51% yield. ^{1}H NMR (400 MHz, MeOD) δ 8.61 (s, 1H), 8.00 (d, J = 8.6 Hz, 2H), 7.44 (d, J = 8.4 Hz, 2H), 6.07 (s, 2H), 3.43 – 3.34 (m, 2H), 2.49 (s, 6H), 1.74 – 1.60 (m, 2H), 1.40 (s, 6H), 1.00 (t, J = 7.4 Hz, 3H) ppm. ^{13}C NMR (101 MHz, MeOD) δ 169.37, 157.08, 144.48, 142.37,

139.41, 136.82, 132.28, 129.70, 129.32, 122.45, 42.91, 23.71, 14.72, 14.56, 11.78 ppm. (ESI-TOF)- calculated for $C_{23}H_{26}BF_2N_3O$ (M^-) (m/z): 408.2064; experimental (M^-) (m/z): 408.2063.

Synthesis of the control compound, TPP-BDP. For the preparation of the TPP-BDP compound, 1 equivalent of the acidic fluorophore **BDP-COOH** (200 mg) was dissolved in 20 mL of $CHCl_3$ at room temperature under a nitrogen atmosphere. Subsequently, DIPEA (1.01 equivalents, 116 µL) and the coupling agent TBTU (1.01 equivalents, 210 mg) were added, and the solution was stirred for 30 minutes to activate the acid functionality. Then, the bromide precursor of (3-aminopropyl)-triphenylphosphonium (1.01 equivalents, 220 mg), previously synthesized as described by Madak *et al.,*[4] was added to the reaction mixture. The reaction was allowed to proceed at room temperature under continuous stirring for 24 hours. After completion, 30 mL of $CHCl_3$ were added, and the mixture was sequentially washed with 30 mL of 1.0 M HCl (twice), 30 mL of saturated $NaHCO_3$ solution (twice) and finally with 20 mL of distilled water. The organic layer was then dried over anhydrous Na_2SO_4, filtered, and concentrated using a rotary evaporator. Purification was accomplished by column chromatography employing a hexane:ethyl acetate elution system in ratios of 7:3 and 1:1, affording a purple solid that was subsequently dried in a vacuum oven at 45 °C for 24 hours.

TPP-BDP: Starting materials: 220 mg of (3-aminopropyl)-triphenylphosphonium, 200 mg of BDP-COOH. Result after precipitation: Purple solid, 0.268 g, 43% yield. ^{1}H NMR (400 MHz, DMSO-d_6) δ 8.81 (s, 1H), 8.03 (d, J = 8.0 Hz, 2H), 7.95 – 7.87 (m, 3H), 7.87 – 7.73 (m, 13H), 7.50 (d, J = 8.0 Hz, 2H), 6.19 (s, 2H), 3.77 – 3.64 (m, 2H), 3.50 – 3.41 (m, 2H), 2.46 (s, 6H), 1.90 – 1.80 (m, 2H), 1.33 (s, 6H) ppm. ^{13}C NMR (101 MHz, DMSO-d_6) δ 165.58, 155.15, 142.56, 141.00, 136.86, 134.92, 134.73, 133.62, 133.58, 133.52, 130.30, 130.18, 128.13, 127.99, 121.48, 118.79, 117.94, 14.19, 14.06 ppm. (ESI-TOF)+ calculated for $C_{41}H_{40}BF_2N_3OP^+$ (M^+) (m/z): 670.2965; experimental (M^+) (m/z): 670.2973.

Optical measurements. Stock solutions of compounds were prepared at a concentration of 20 mM in dimethyl sulfoxide. For UV-vis absorption measurements, the stock solutions were diluted to a final concentration of 15 µM in acetonitrile. The excitation wavelength (λ_{ex}) was selected just below the absorption maximum (470 nm) to ensure full capture of the emission spectrum.

Toxicity assays. The cell viability assays using the MTT method were conducted based on procedures described in previous studies,[5] with some modifications. Initially, cells were cultured in 96-well plates at a density of approximately 5x10^4 cells per well. The cells were seeded in appropriate culture medium and incubated at 37°C with 5% CO_2 for 24 to 48 hours to allow for cell adhesion and growth. After this period, the test compounds were added to the wells at varying concentrations, and the cells were incubated for an additional 4 hours under the same conditions. At the end of the treatment, 10 µL of an MTT solution (5 mg/mL in PBS 1x) was added to each well, and the plate was incubated for another 3 hours at 37°C, allowing metabolically active cells to convert the MTT into formazan crystals. After the MTT incubation, the medium was carefully removed, and the formazan crystals formed were dissolved by adding 100 µL of dimethyl sulfoxide (DMSO) to each well. The plate was gently shaken for 10 minutes to ensure complete dissolution of the crystals. The absorbance was measured using a microplate reader (Multiskan FC) at a wavelength of 570 nm, with a reference filter at 630 nm. The absorbance intensity was proportional to the number of viable cells, allowing for the assessment of cell viability in response to the tested compounds.

Confocal Microscopy. The preparation of IBIDI plates for confocal microscopy was conducted as follows, following the procedure described in previous.[5] MCF7 (breast cancer), A549 (lung carcinoma), HT-29 (colon adenocarcinoma), HEK-293 (human embryonic kidney), and HMEC-1 (human microvascular endothelial) cells were cultured in IBIDI 4-well plates and allowed to grow for 24 hours at 37°C in a 5% CO_2 atmosphere to ensure adhesion to the well bottom. After the growth period, the cells were washed three times with phosphate-buffered saline (PBS) to remove any residual culture medium. The appropriate treatment for each well was then added, including the mitochondrial probe (0.5 µM for 30 minutes), Mitotracker Deep Red FM (100 nM for 30 minutes), and Hoechst 33342 (0.1 mg/mL for 5 minutes). The cells were incubated with this solution for the specified time at 37°C. After incubation, the solution was removed, and the cells were washed three times with PBS to remove any unbound probes. The wells were then filled with culture medium without Fluobrite to minimize interference with the fluorescence emitted during confocal imaging. Images were acquired using a Leica TCS SP8 confocal microscope equipped with a 60x oil immersion objective. The excitation and emission settings for each

channel were as follows: for nuclear staining (Hoechst 33342), a 405 nm laser was used for excitation, with emission collected between 410-465 nm; for the mitochondrial probes, a 488 nm laser was used for excitation, with emission collected between 500-550 nm; and for Mitotracker Deep Red, excitation was performed with a 633 nm laser, with emission collected between 650-750 nm. Separate images were captured for each channel (hoechst 33342, synthetic probes, and Mitotracker Deep Red) in the five cell lines: MCF7, A549, HT-29, HEK293, and HMEC-1.

Live cell time-lapse imaging. MCF-7 cells were seeded at a density of 200.000 cells per dish in WillCo-dish® glass bottom dishes and allowed to adhere overnight at 37 ˚C in humidified atmosphere with 5% of CO_2. The next day, the cells were incubated with Hoechst 33258 (1µg/µL) for 30 minutes; then the medium was replaced with DMEM FluoroBrite. Dishes were then transferred to a heated stage (37 °C) in a 5% CO_2 environment above a Plan-Neofluar 20x/0.50 NA oil-immersion objective lens on a Zeiss Apotome.2 fluorescent microscope equipped with a Colibri 7 LED light source. A live cell time-lapse imaging experiment was performed by taking an image of 3 random fields of view (FOV) every ten seconds; after 2 minutes of baseline recording, 1µM of TPP or TAPY were added in the imaging media and signal recorded for up to 30 minutes. Time-lapse sequences were imported into ImageJ 2 (National Institutes of Health, Bethesda, MD, USA) and a subtract background function applied to each image with a sliding paraboloid function of 50 pixels. Single cells were selected with the ROI manager using the polygon selection and the area of selected cells, the mean grey value and the integrated density measured. Corrected total cell fluorescence (CTCF) was calculated by subtracting the integrated density to the product of the area and mean fluorescence of the background.

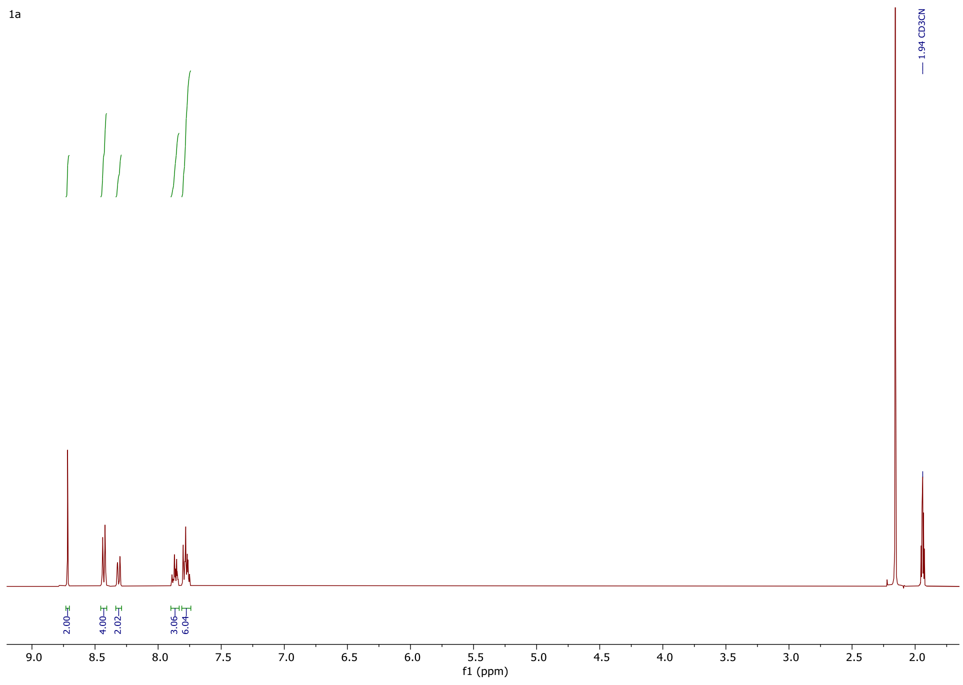

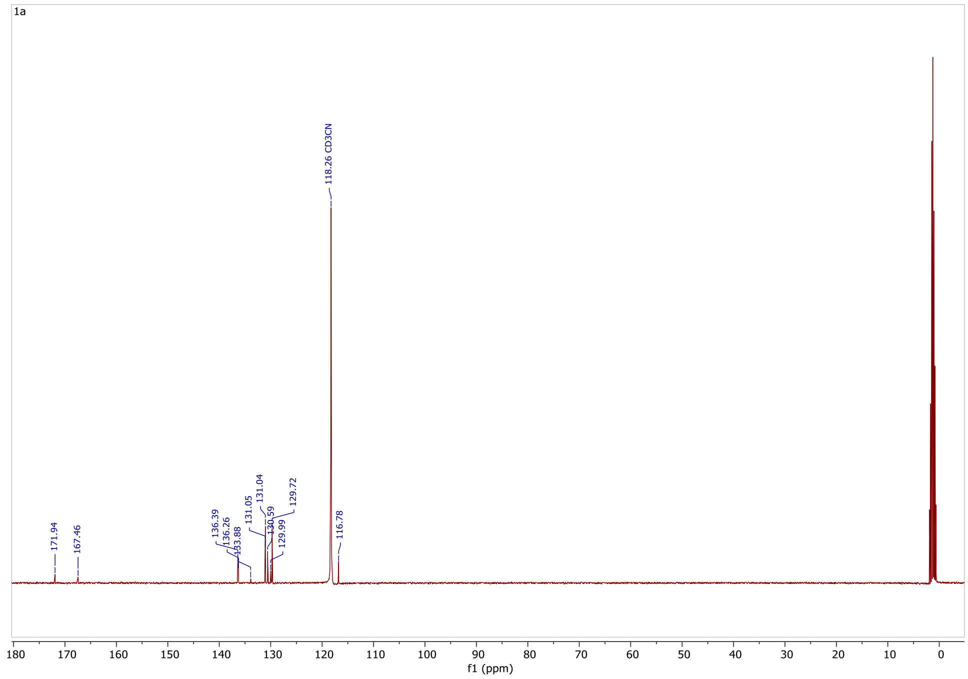

Figure S 1. ^{1}H NMR, ^{13}C NMR (CD_3CN) spectra of compound **1a**.

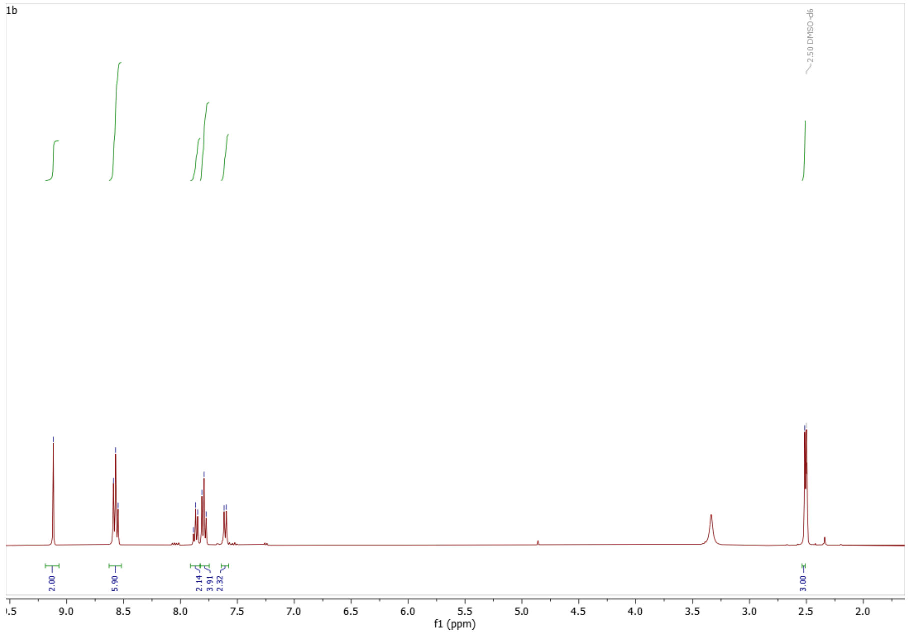

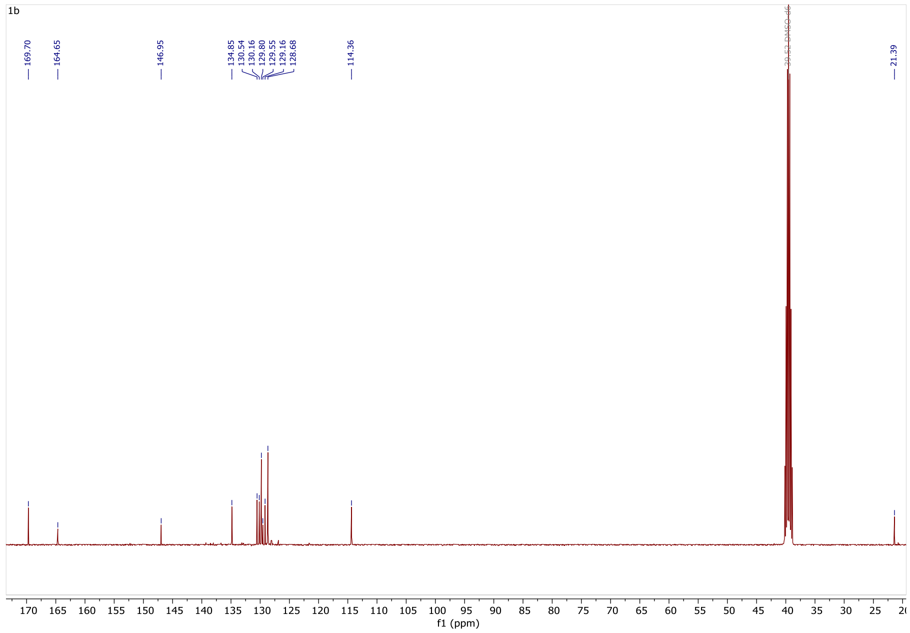

Figure S 2. ^{1}H NMR, ^{13}C NMR (DMSO-d_6) spectra of compound **1b**.

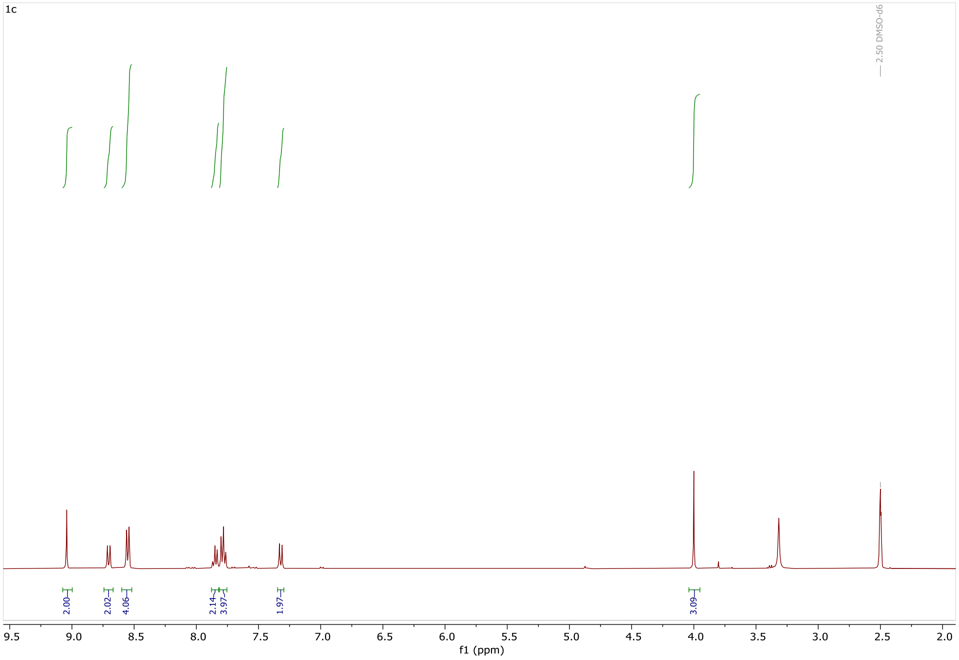

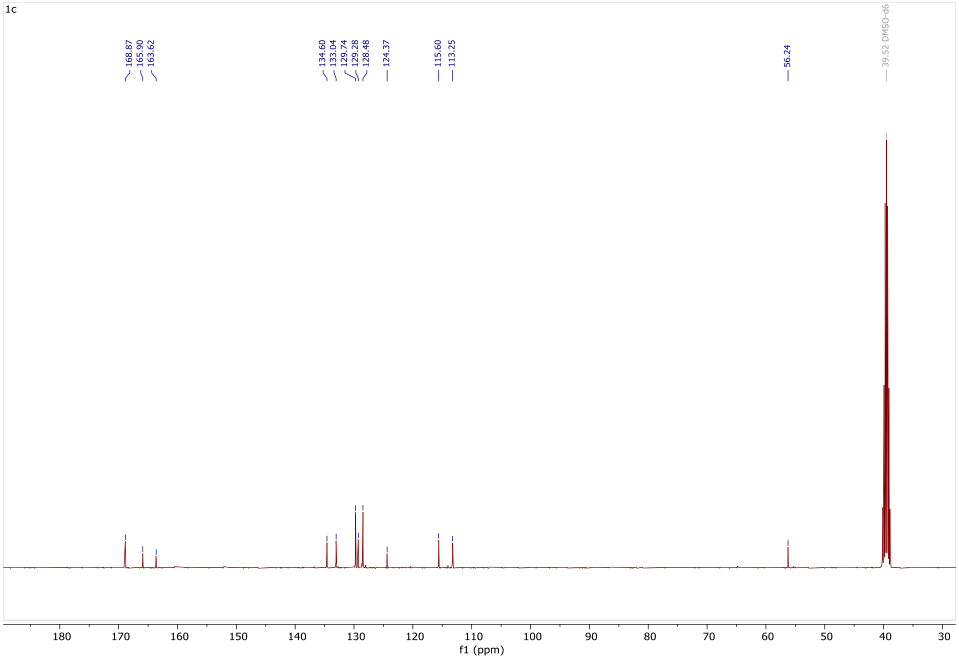

Figure S 3. ^{1}H NMR, ^{13}C NMR (DMSO-d$_6$) spectra of compound **1c**.

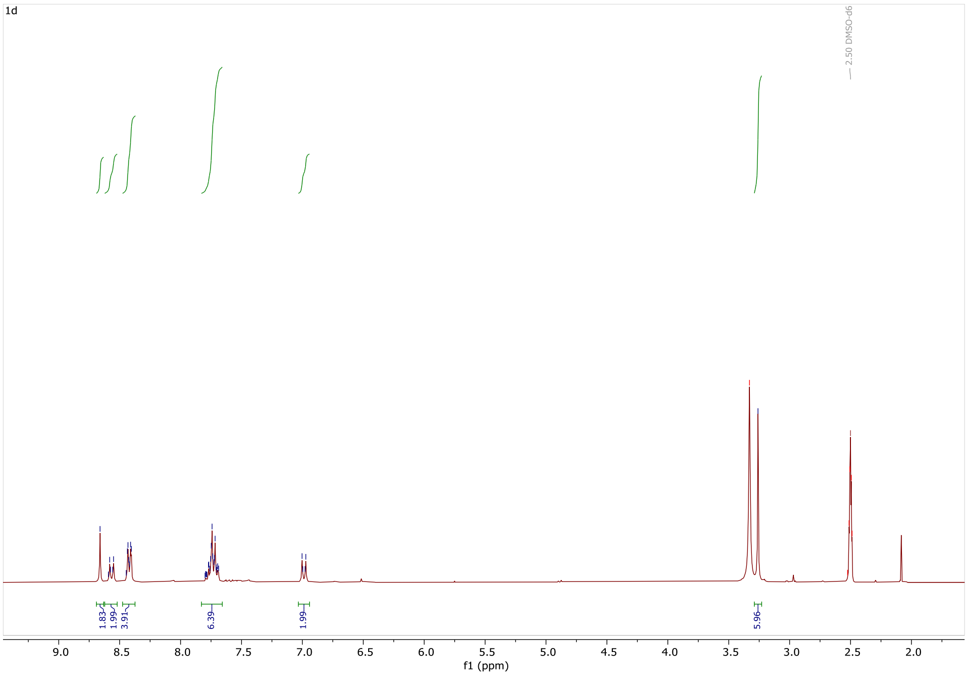

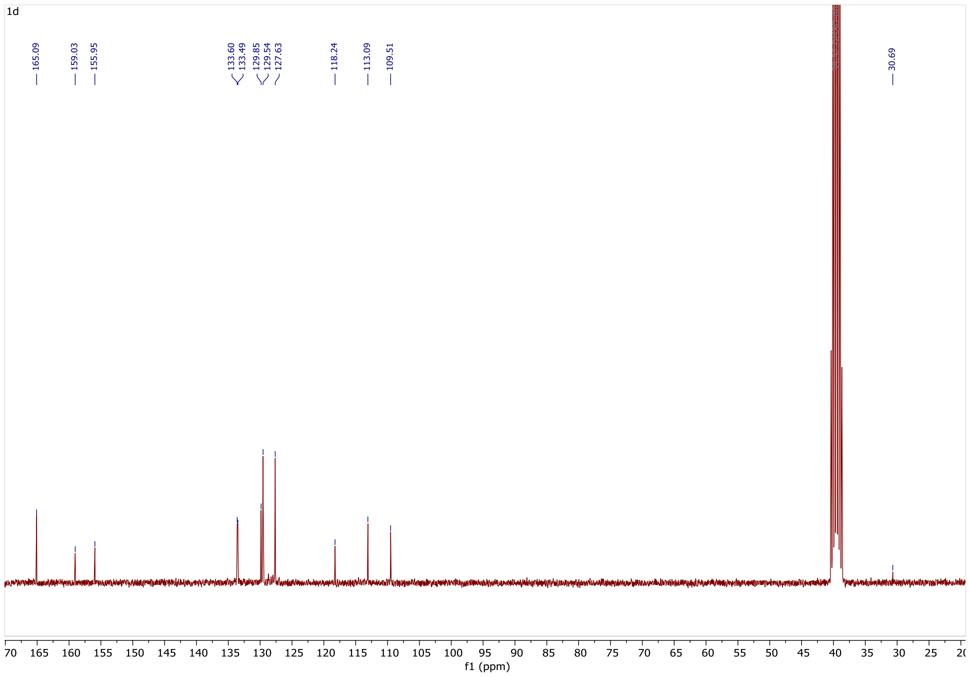

Figure S 4. ^{1}H NMR, ^{13}C NMR (DMSO-d$_6$) spectra of compound **1d**.

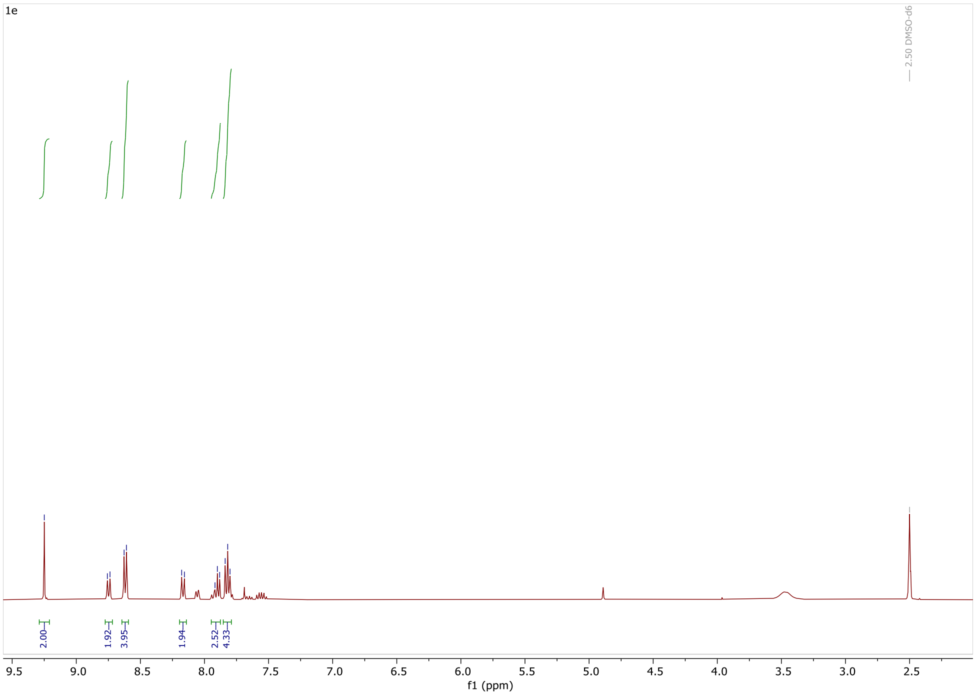

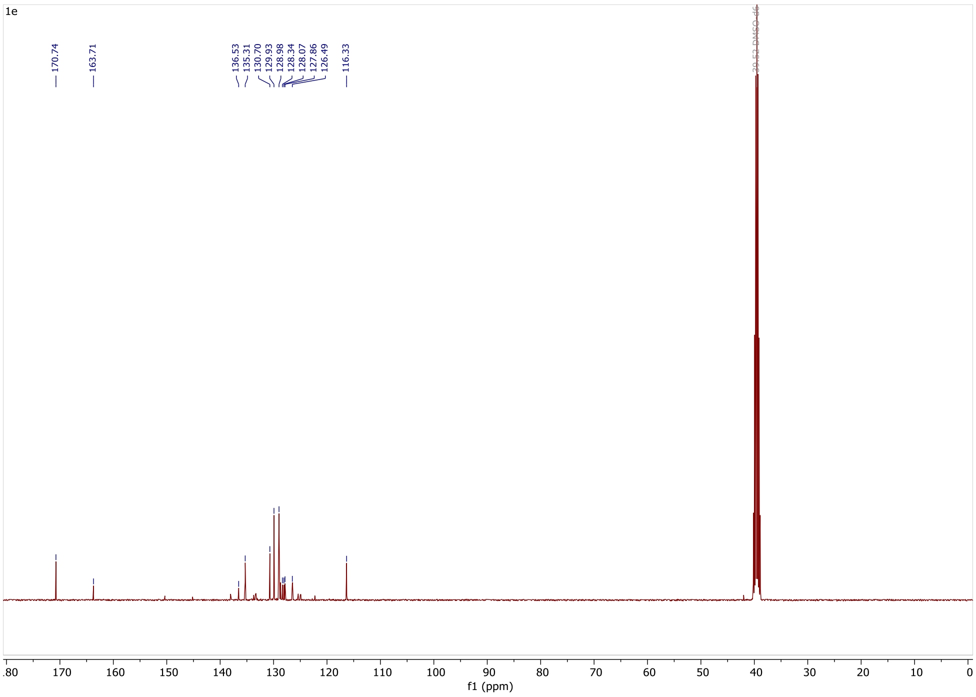

Figure S 5. ^{1}H NMR, ^{13}C NMR (DMSO-d_6) spectra of compound **1e**.

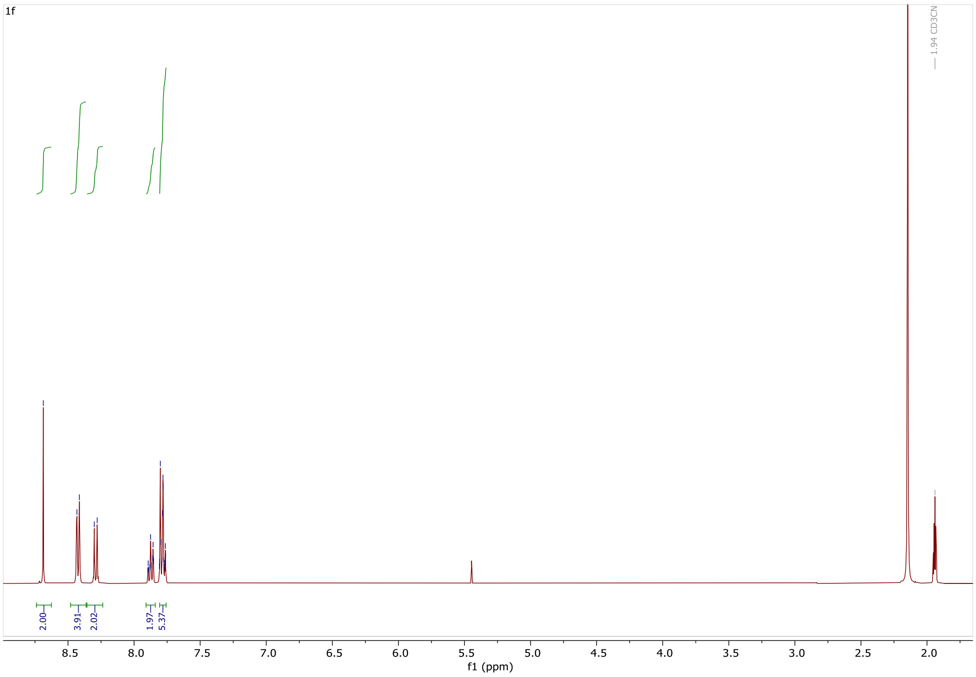

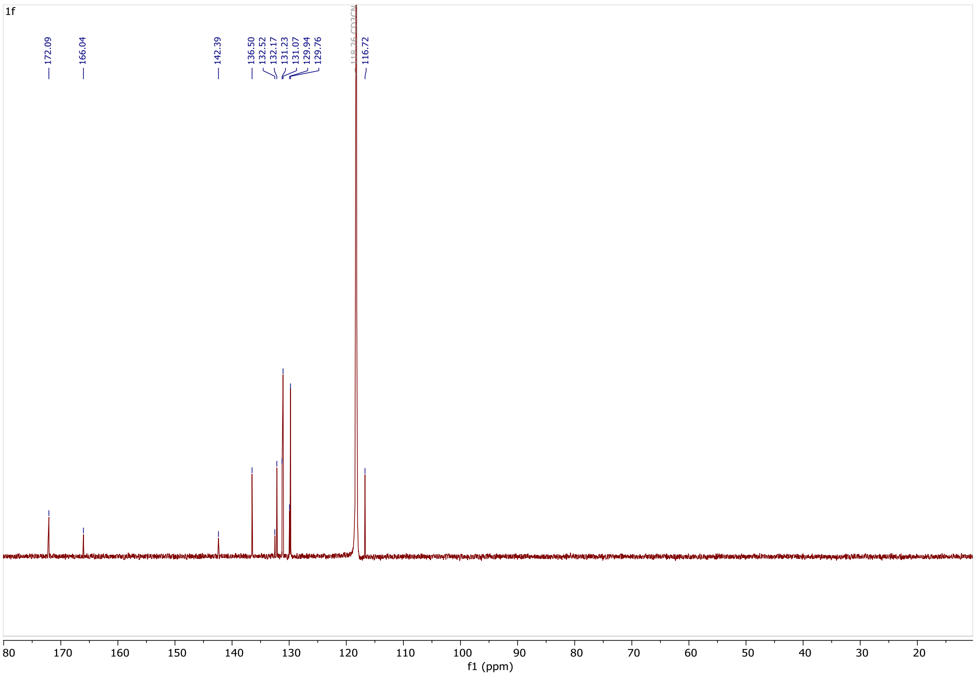

Figure S 6. ^{1}H NMR, ^{13}C NMR (CD_3CN) spectra of compound **1f**.

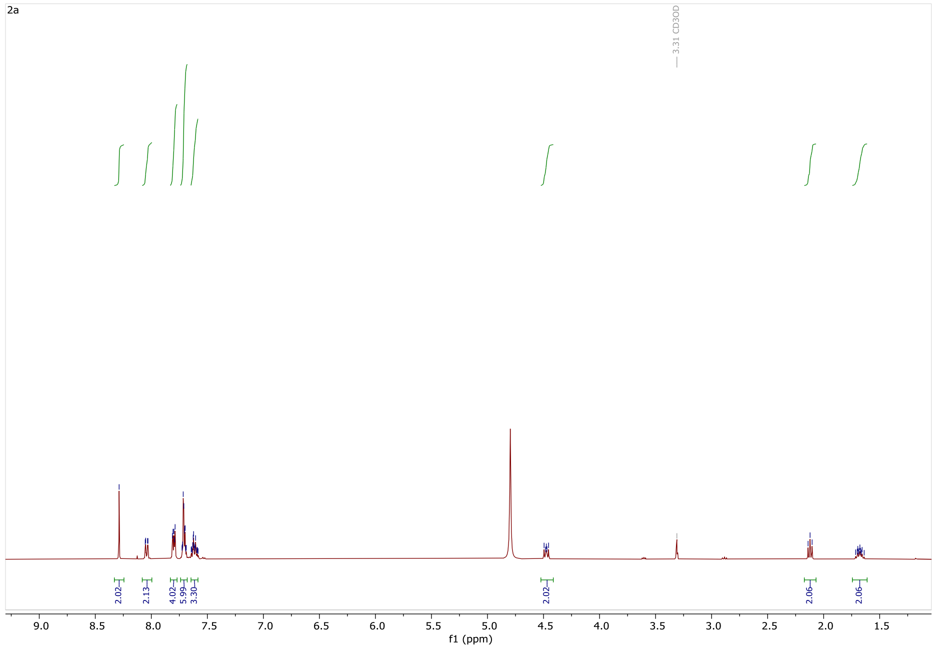

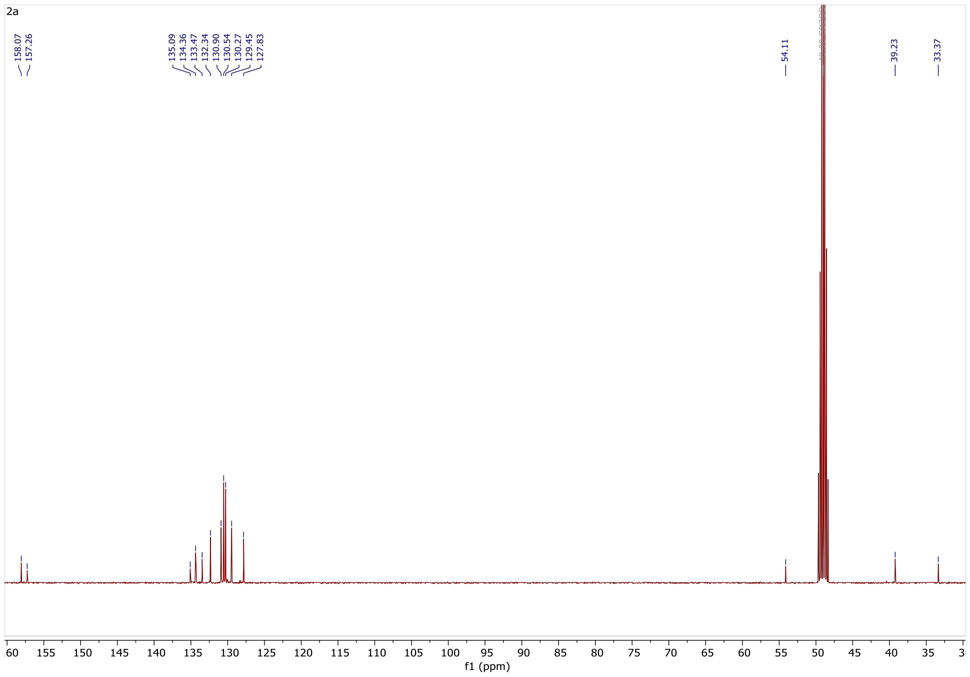

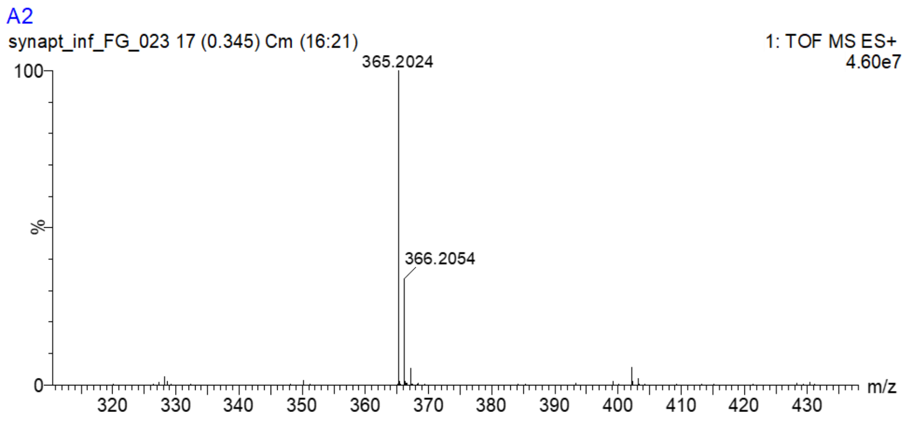

Figure S 7. ^{1}H NMR, ^{13}C NMR (CD_3CN) and Mass spectra of compound **2a.**

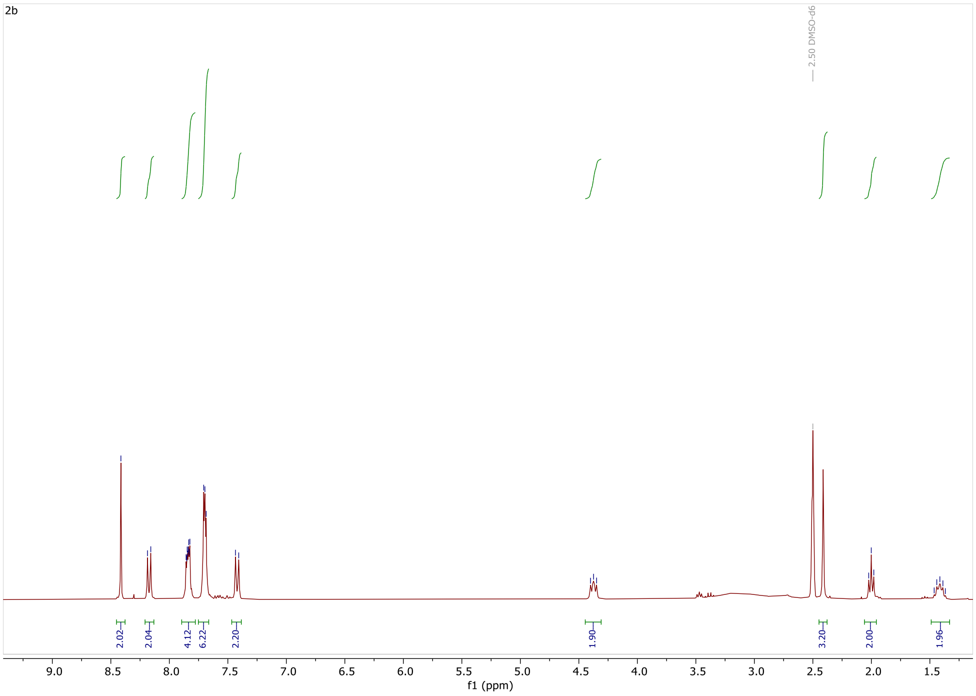

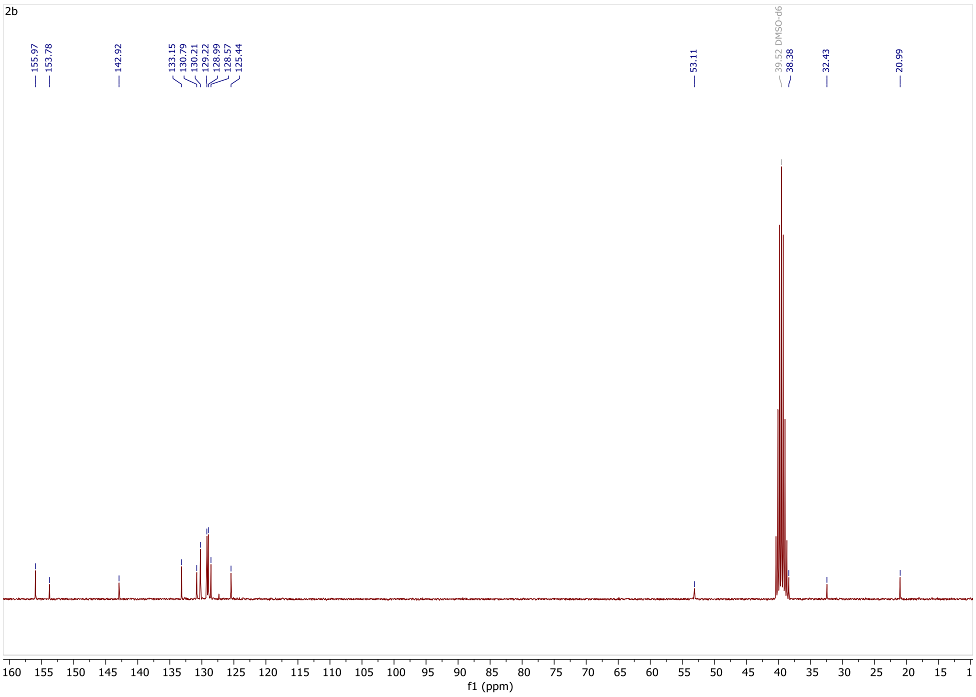

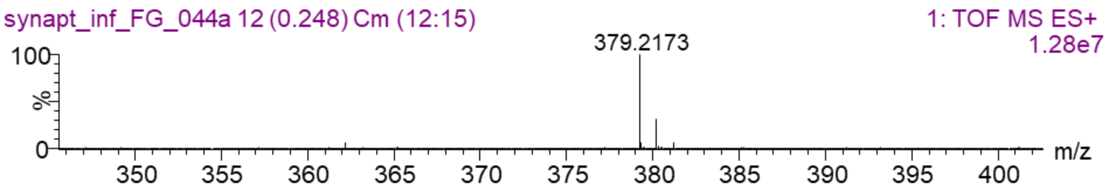

Figure S 8. ^{1}H NMR, ^{13}C NMR (DMSO-d_6) and Mass spectra of compound **2b.**

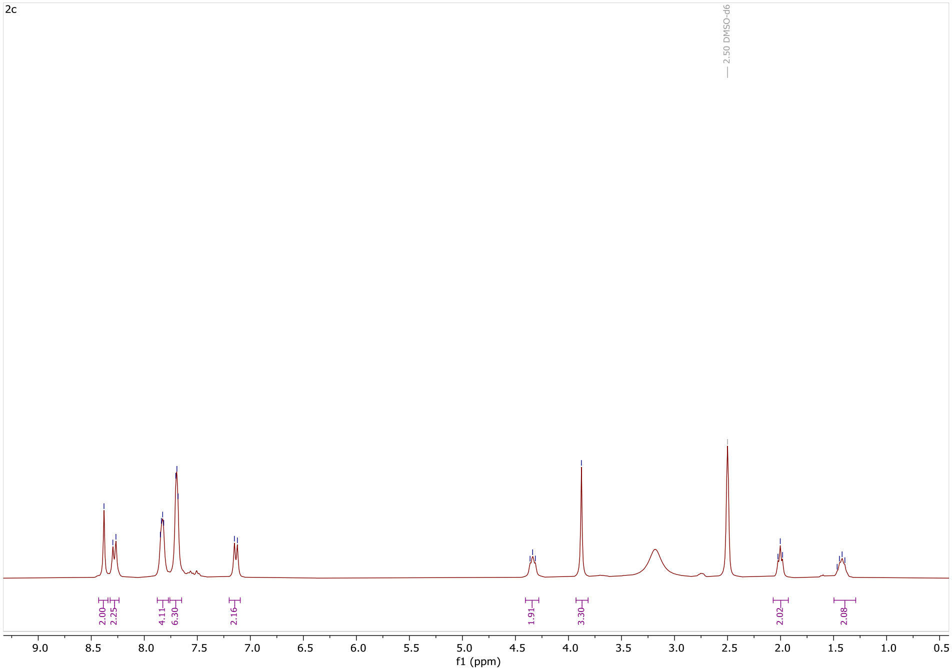

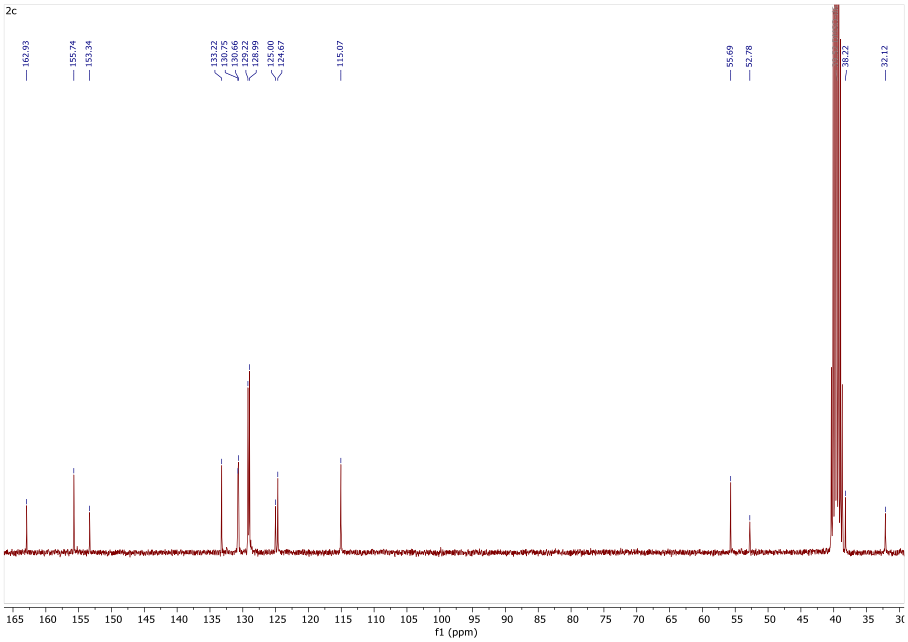

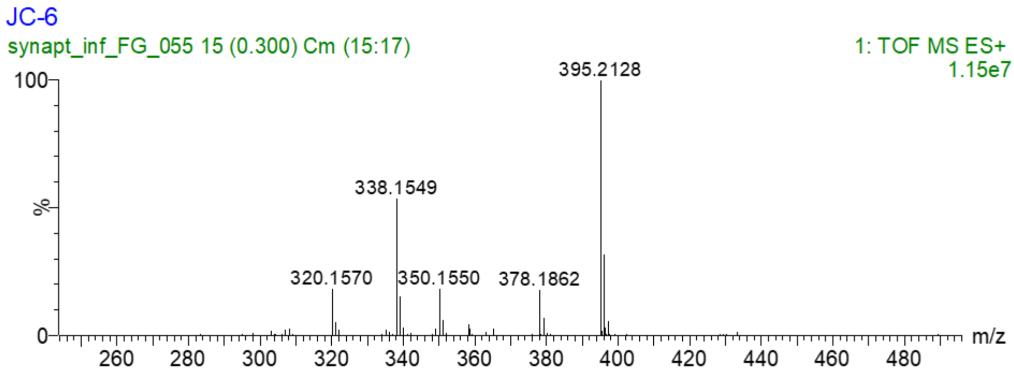

Figure S 9. ^{1}H NMR, ^{13}C NMR (DMSO-d$_6$) and mass spectra of compound **2c.**

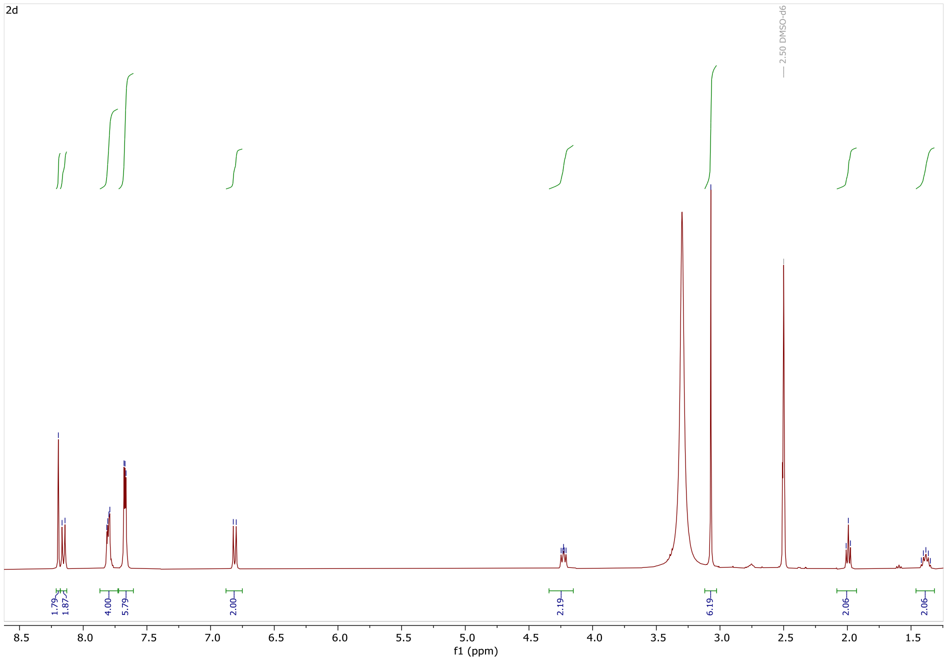

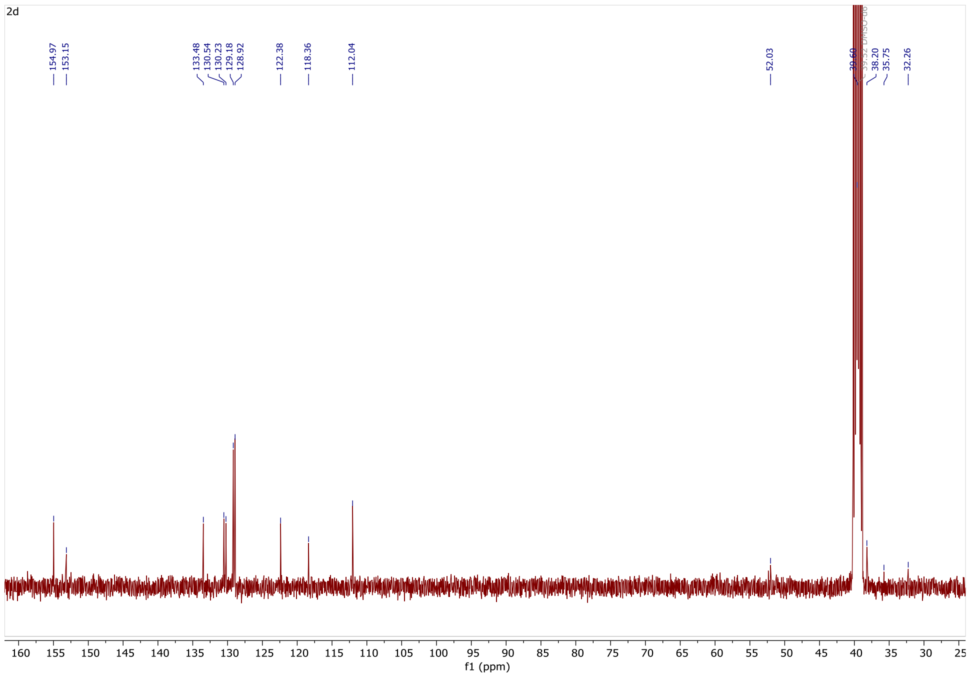

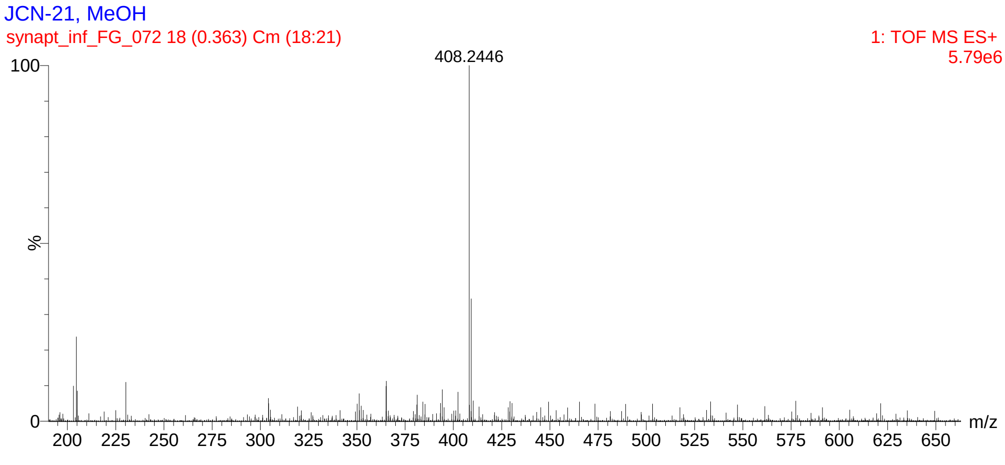

Figure S 10. ^{1}H NMR, ^{13}C NMR (DMSO-d_6) and mass spectra of compound **2d**.

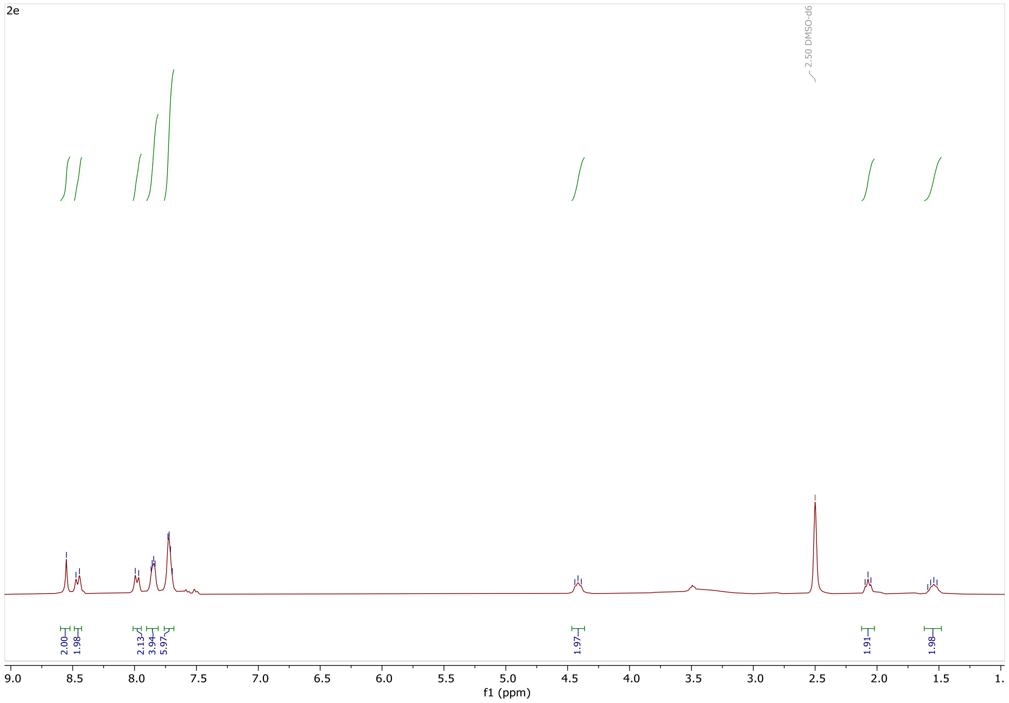

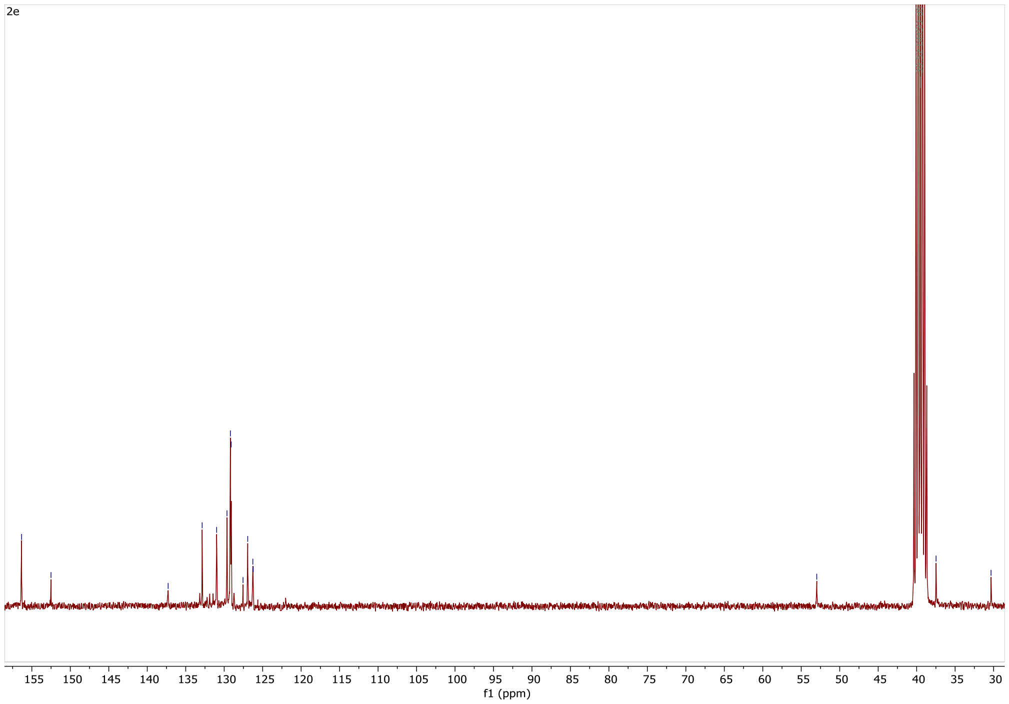

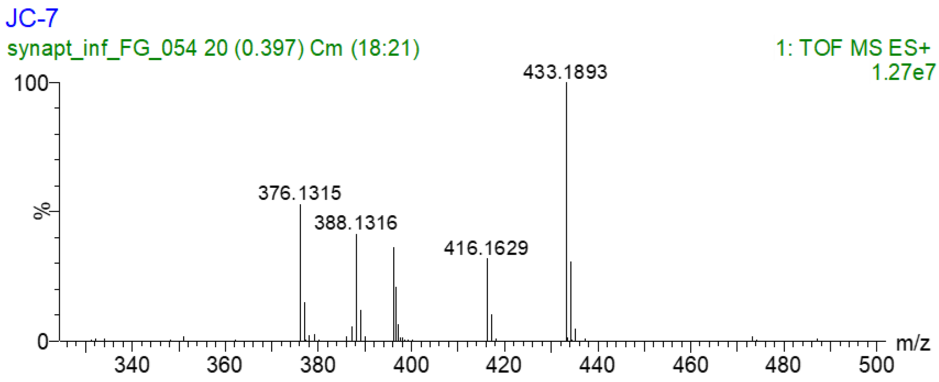

Figure S 11. ^{1}H NMR, ^{13}C NMR (DMSO-d$_6$) and mass spectra of compound **2e**.

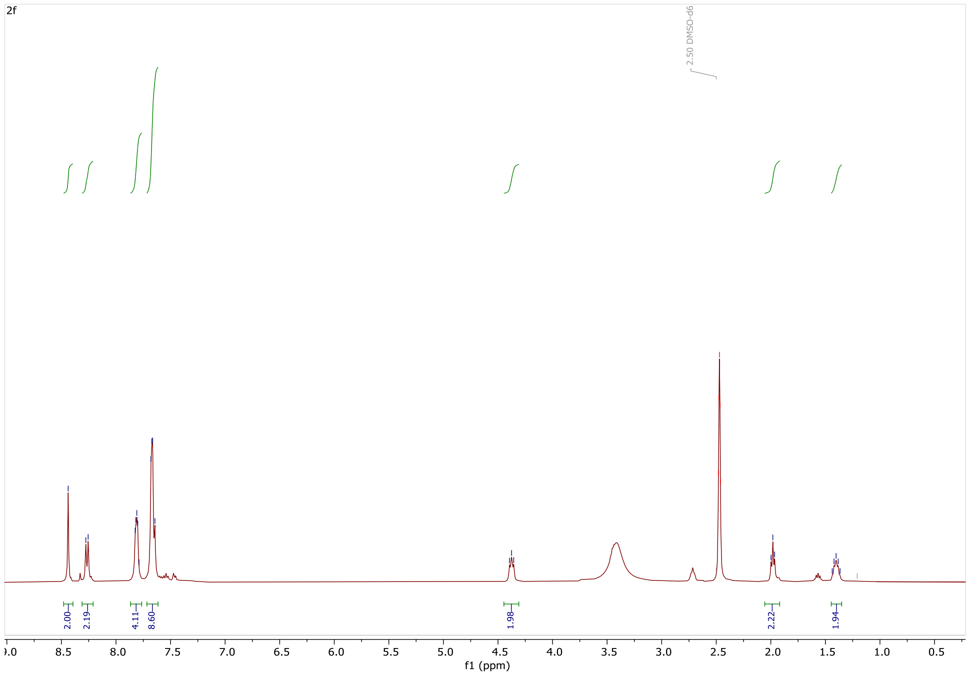

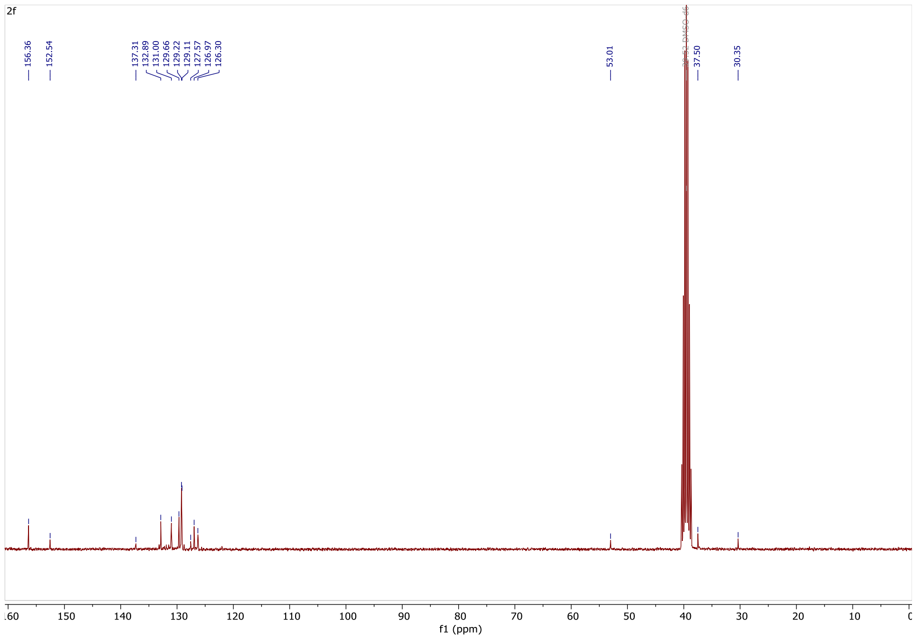

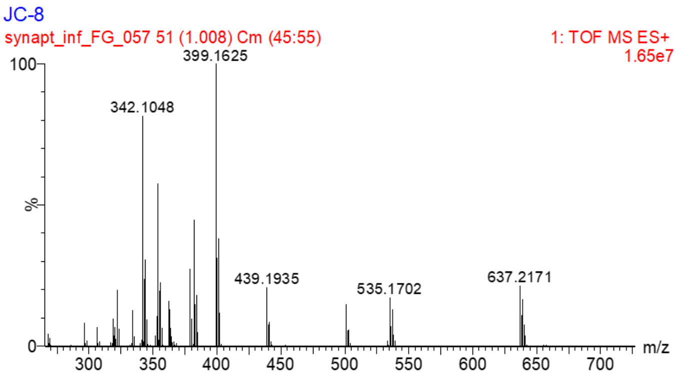

Figure S 12. ^{1}H NMR, ^{13}C NMR (DMSO-d_6) and mass spectra of compound **2f.**

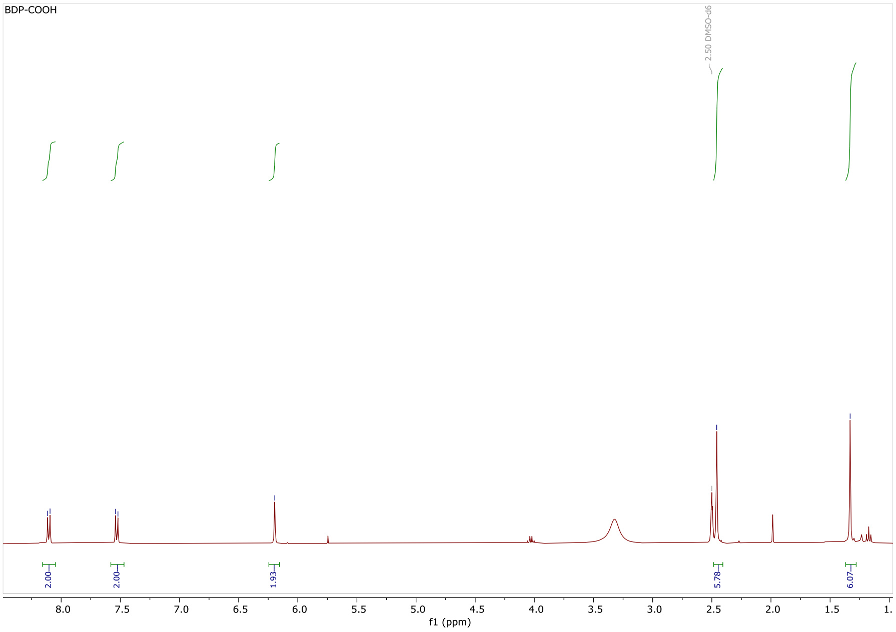

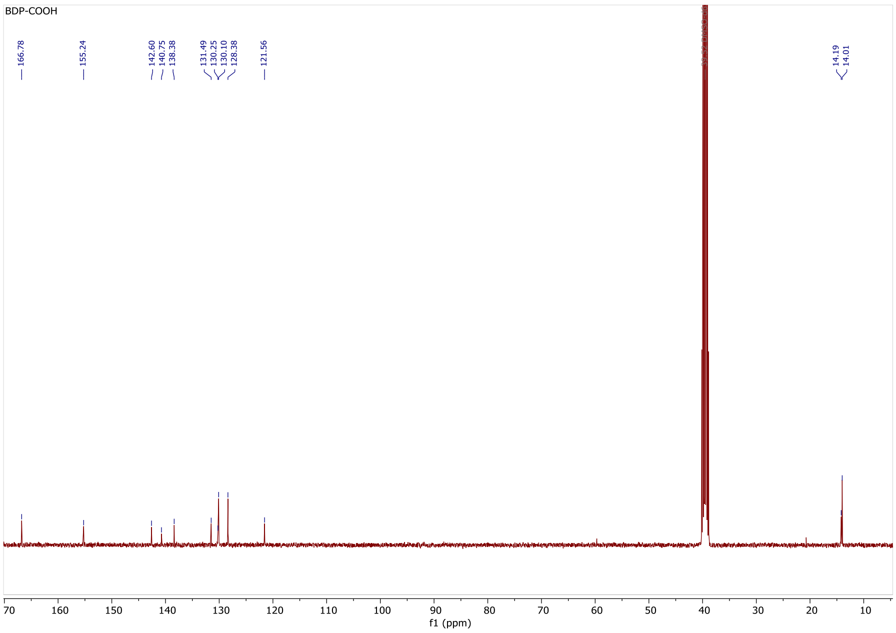

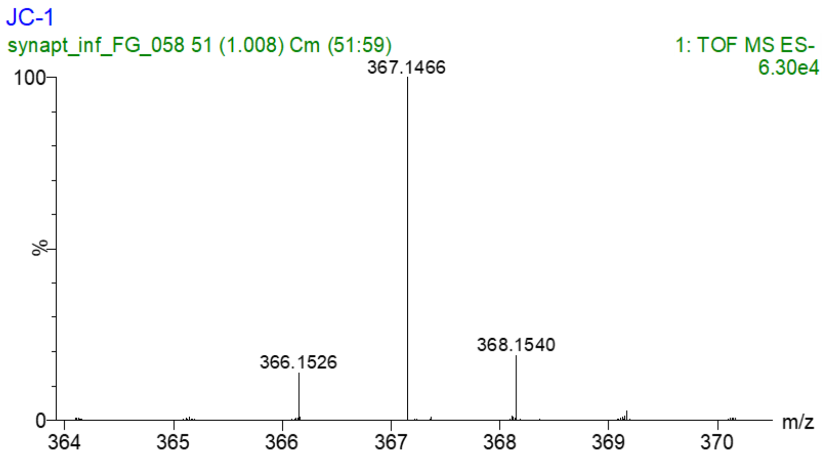

Figure S 13. ^{1}H NMR, ^{13}C NMR (DMSO-d_6) and mass spectra of compound **BDP-COOH.**

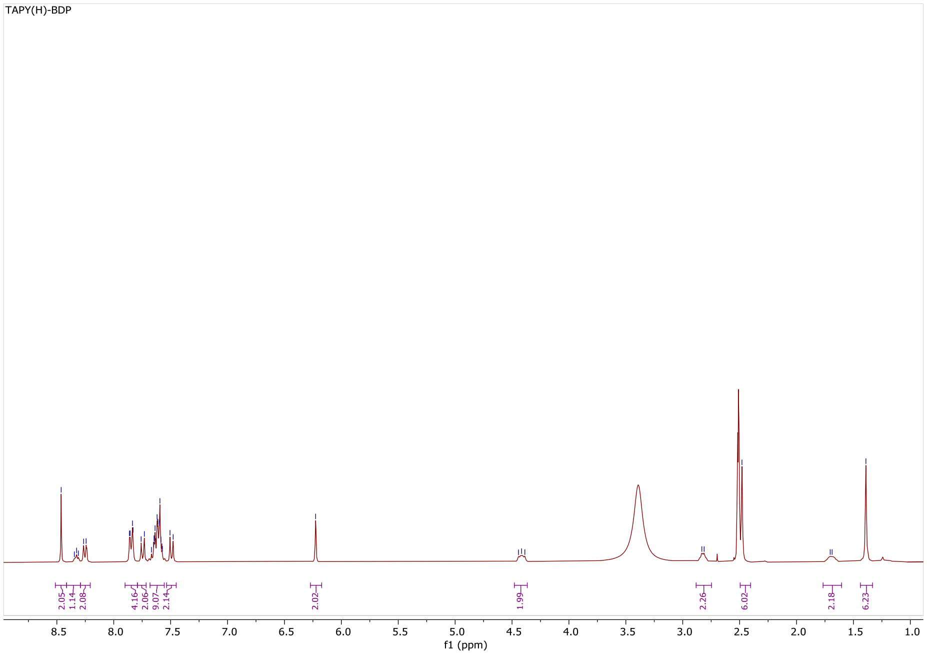

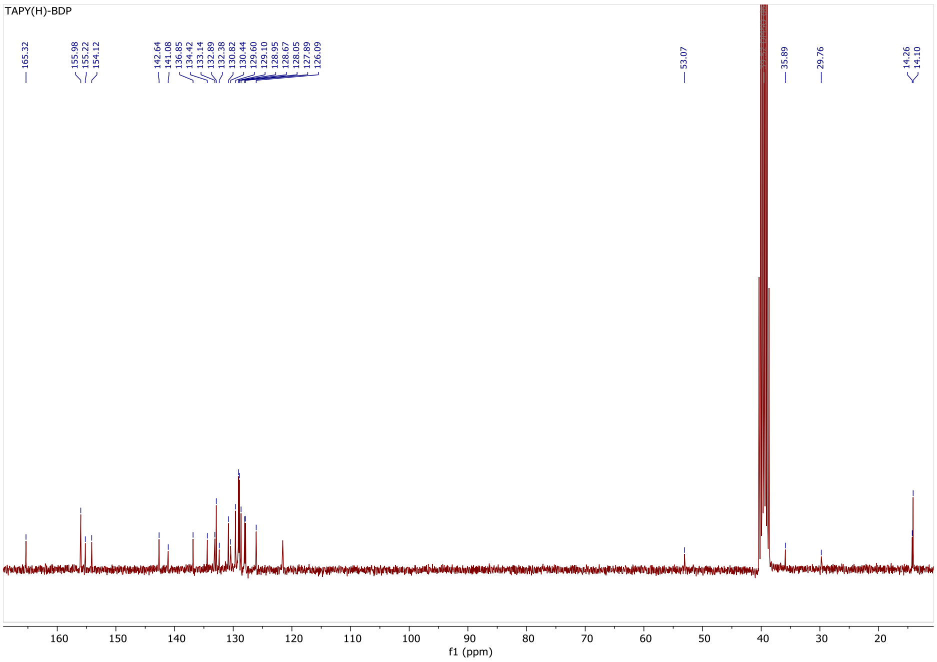

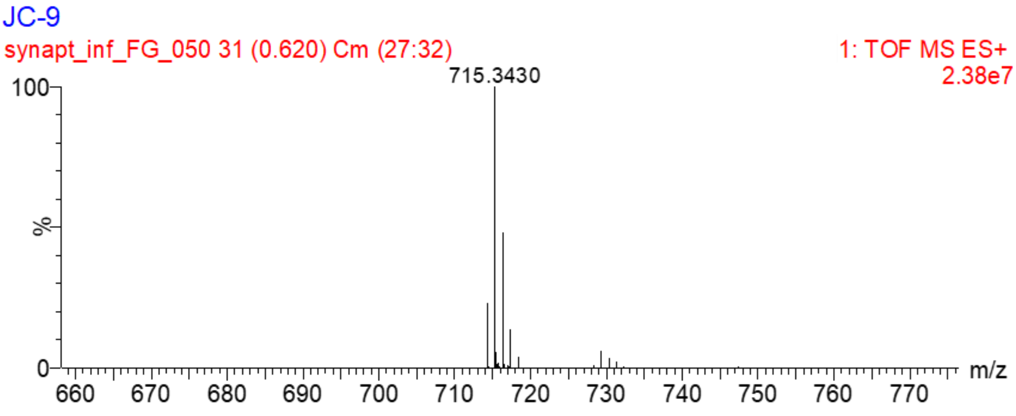

Figure S 14. ^{1}H NMR, ^{13}C NMR (DMSO-d$_6$) and mass spectra of compound **TAPY(H)-BDP.**

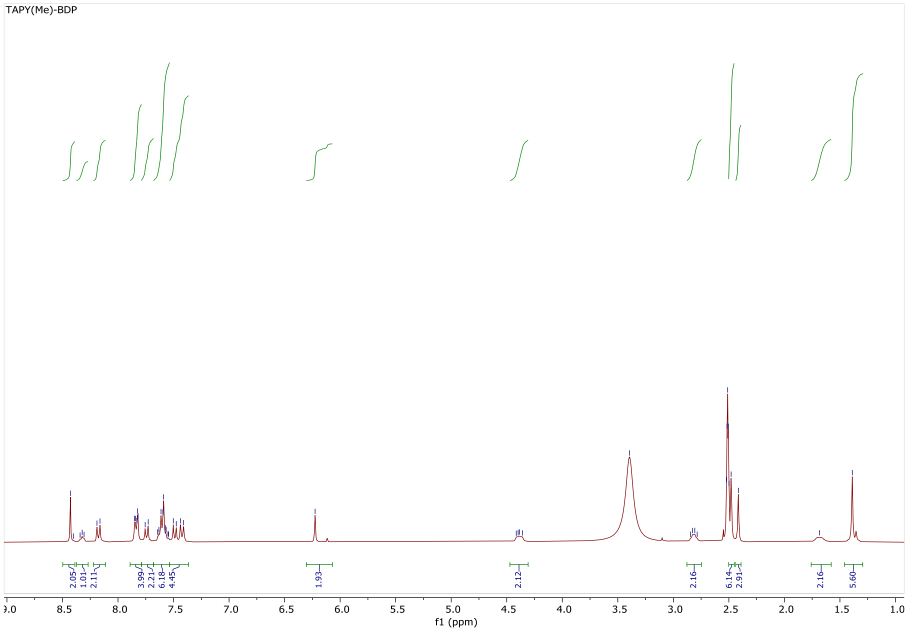

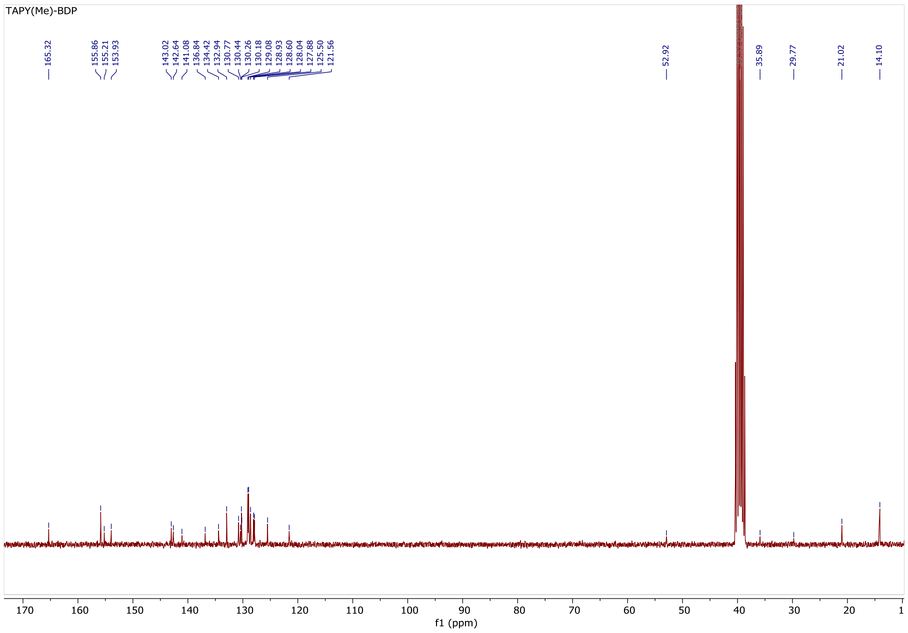

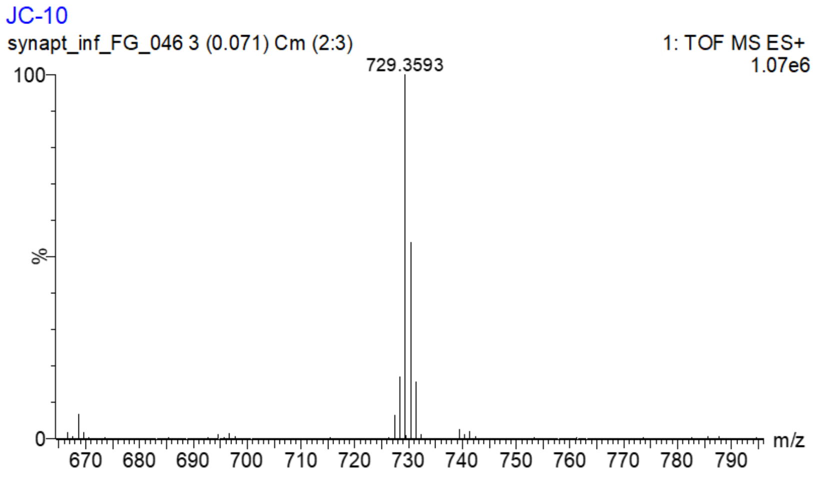

Figure S 15. ^{1}H NMR, ^{13}C NMR (DMSO-d$_6$) and mass spectra of compound **TAPY(Me)-BDP.**

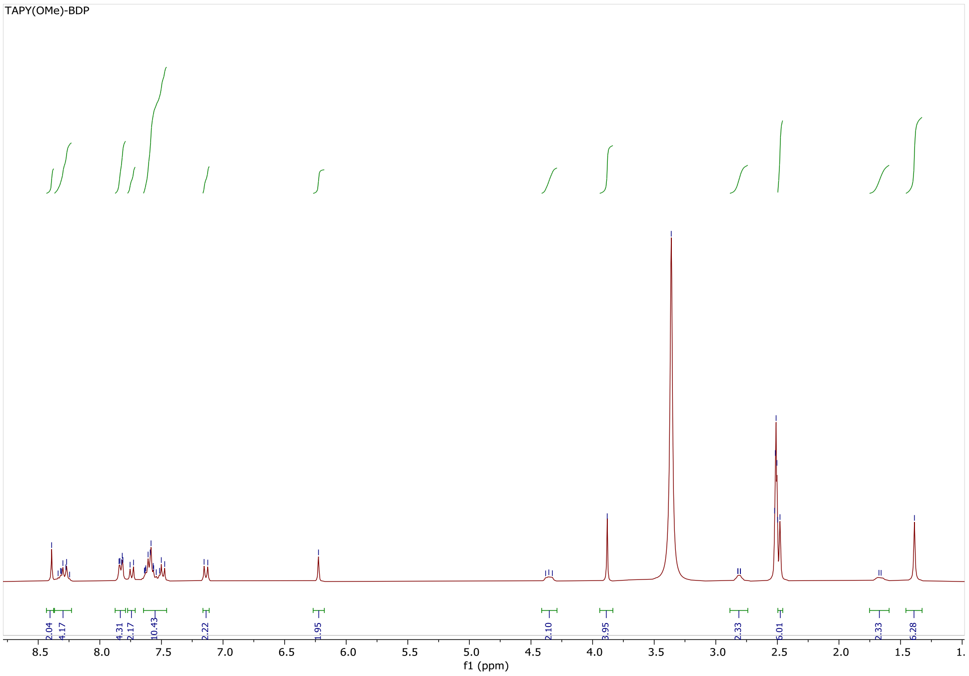

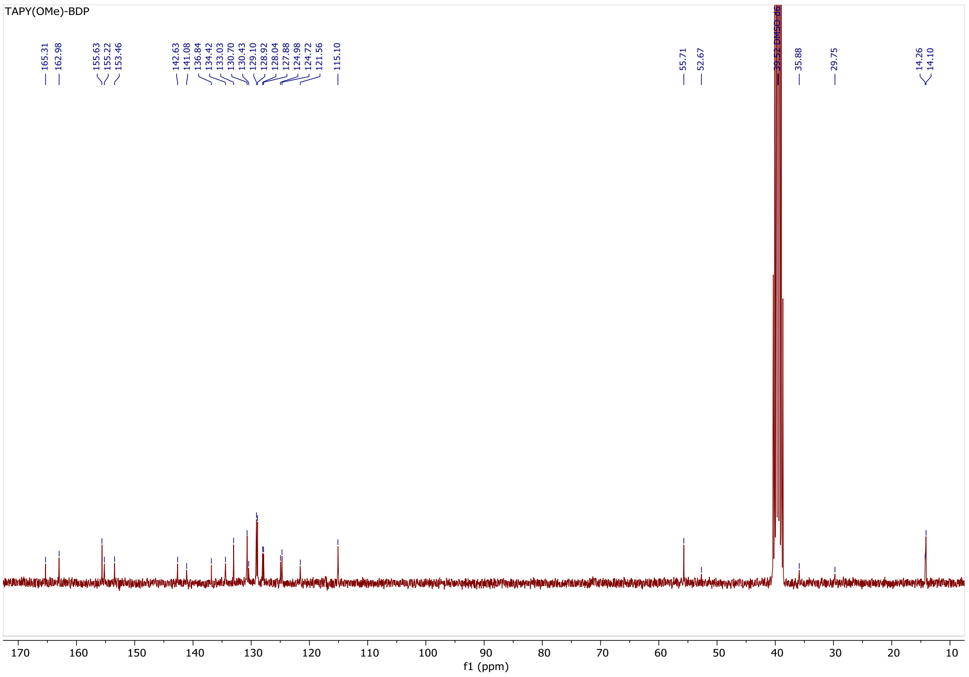

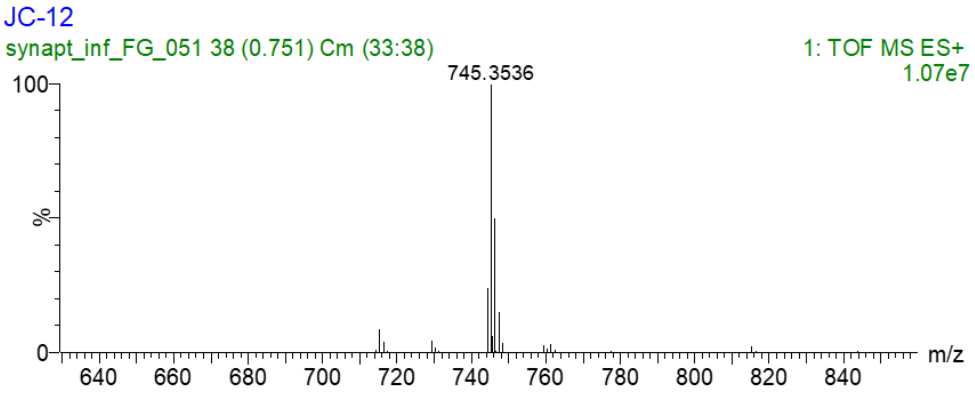

Figure S 16. ^{1}H NMR, ^{13}C NMR (DMSO-d_6) and mass spectra of compound **TAPY(OMe)-BDP.**

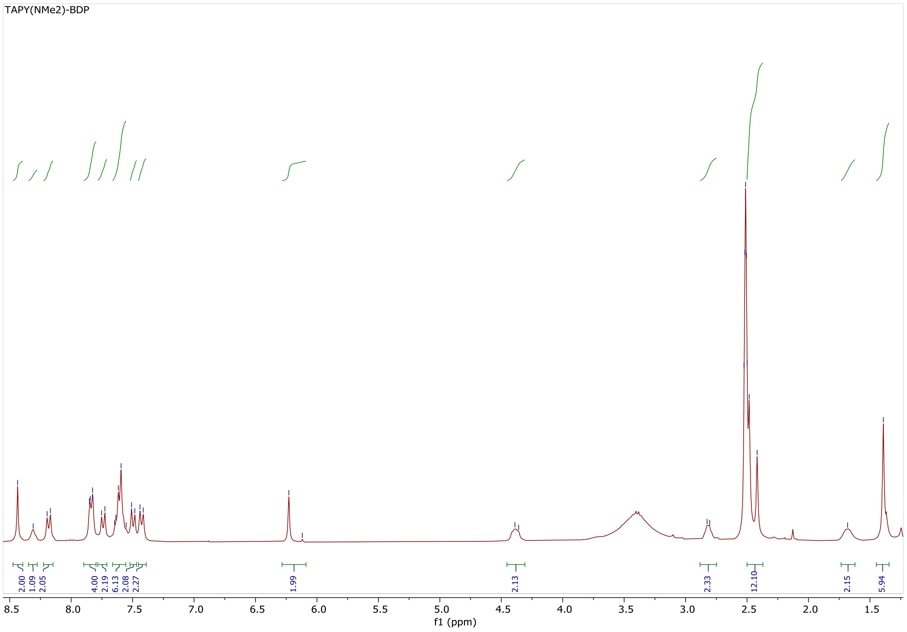

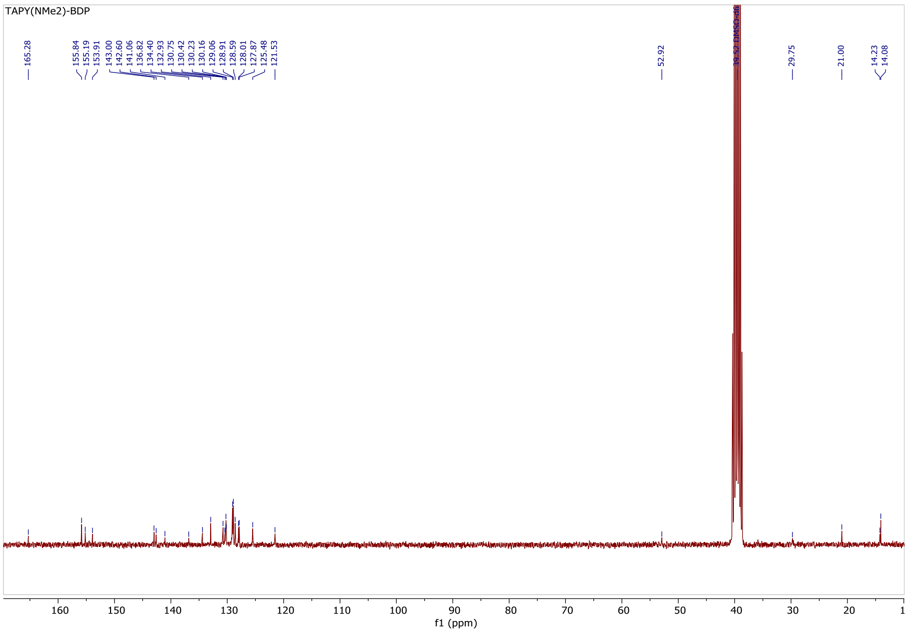

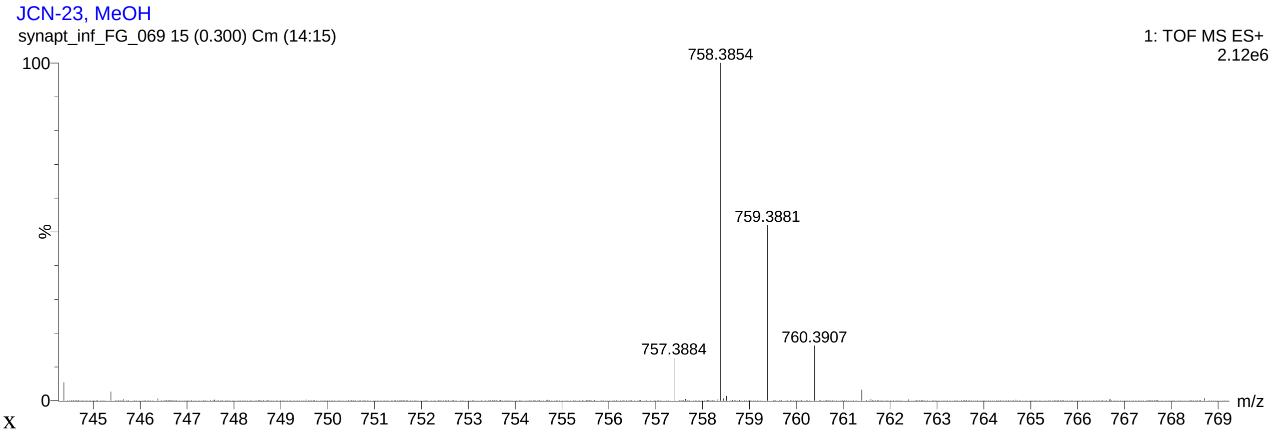

Figure S 17. ^{1}H NMR, ^{13}C NMR (DMSO-d$_6$) and mass spectra of compound **TAPY(NMe$_2$)-BDP.**

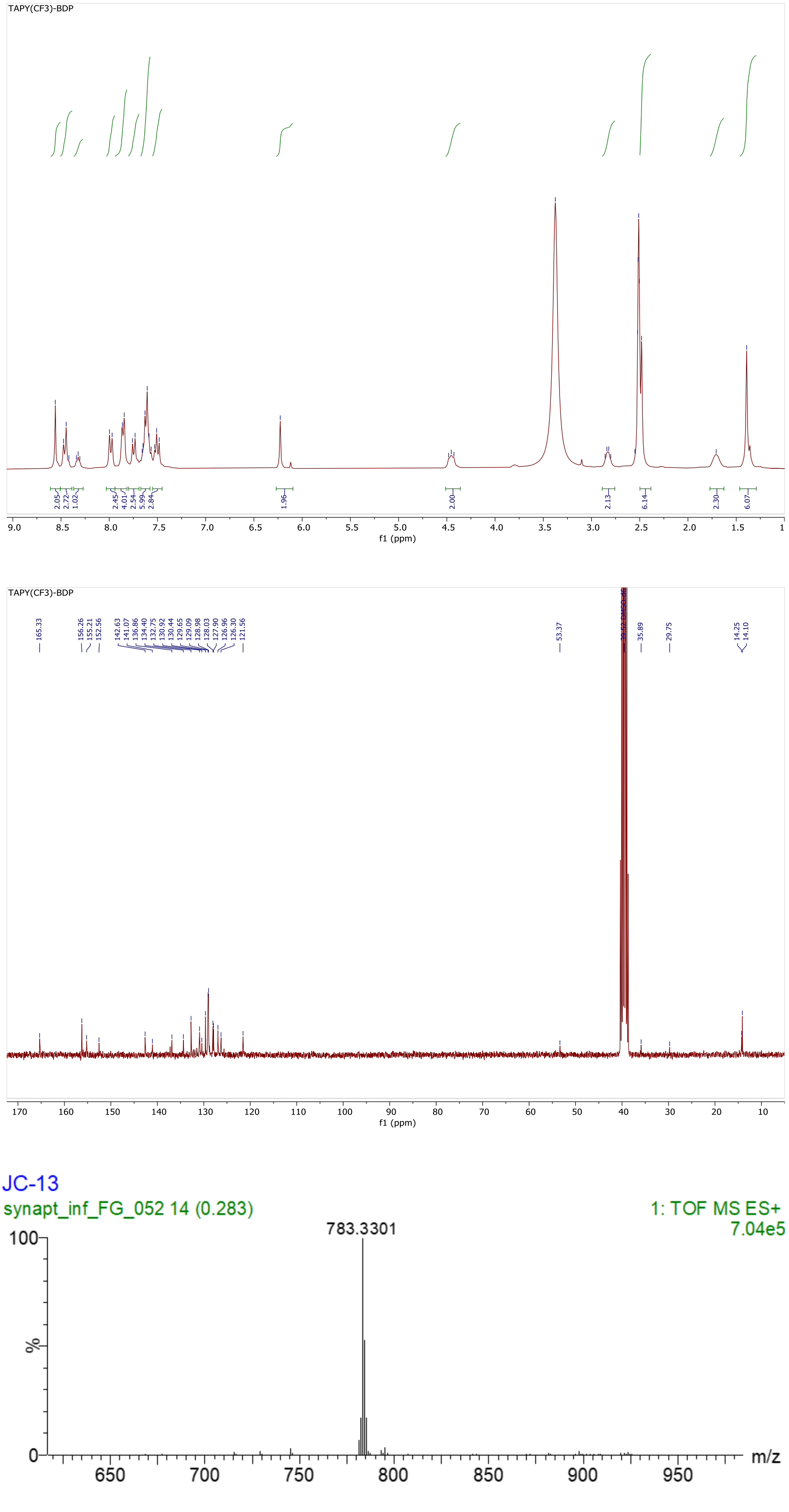

Figure S 18. ^{1}H NMR, ^{13}C NMR (DMSO-d₆) and mass spectra of compound **TAPY(CF₃)-BDP.**

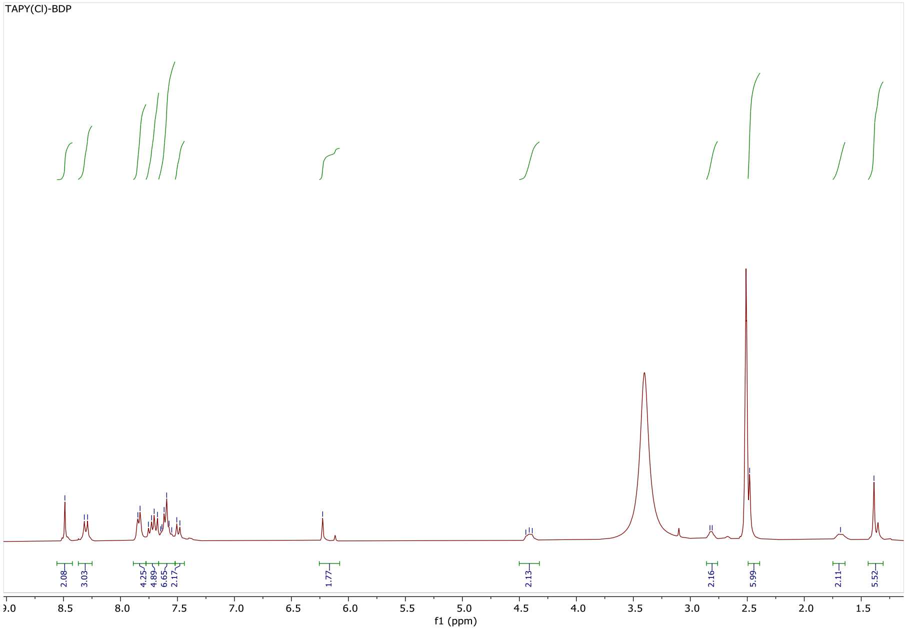

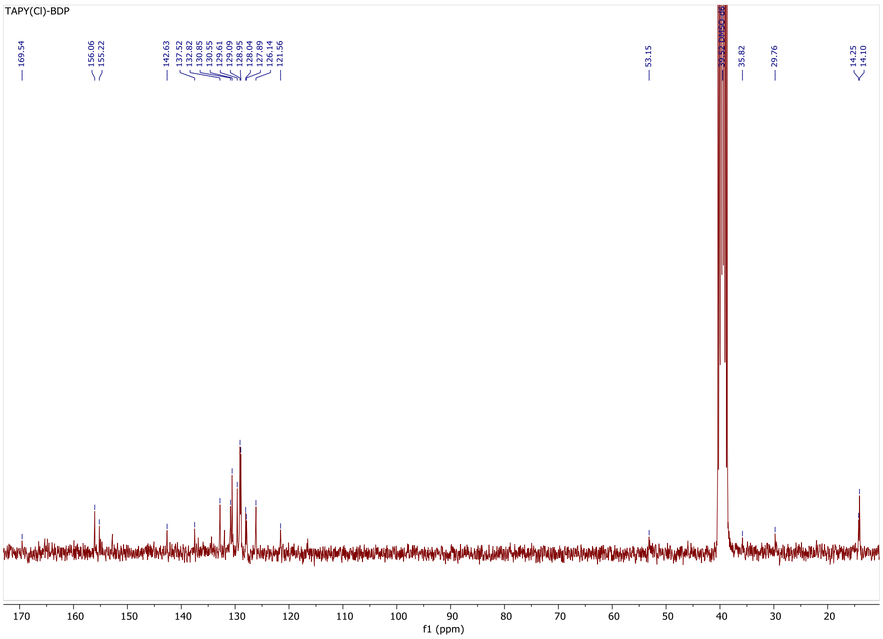

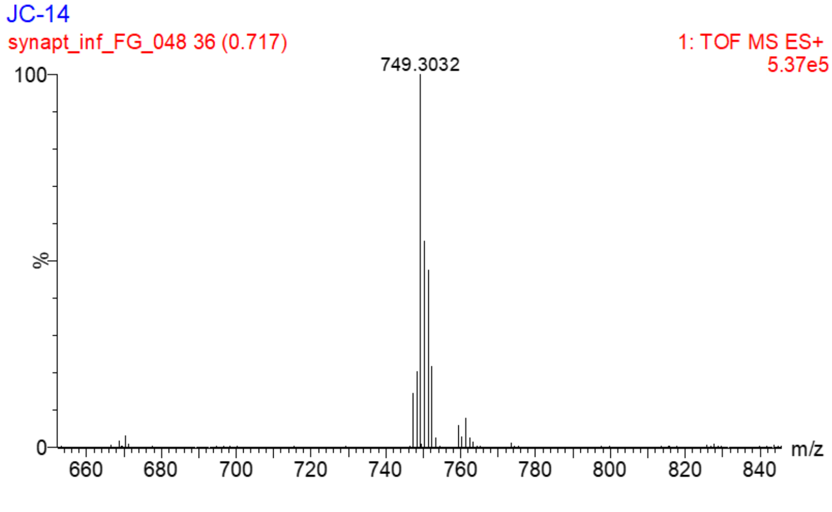

Figure S 19. ^{1}H NMR, ^{13}C NMR (DMSO-d_6) and mass spectra of compound **TAPY(Cl)-BDP.**

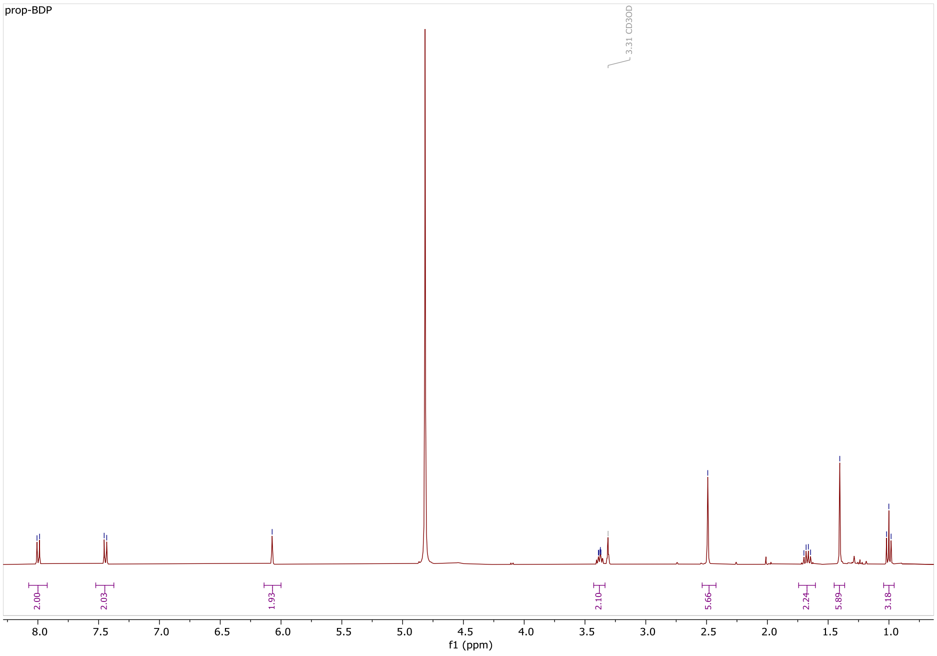

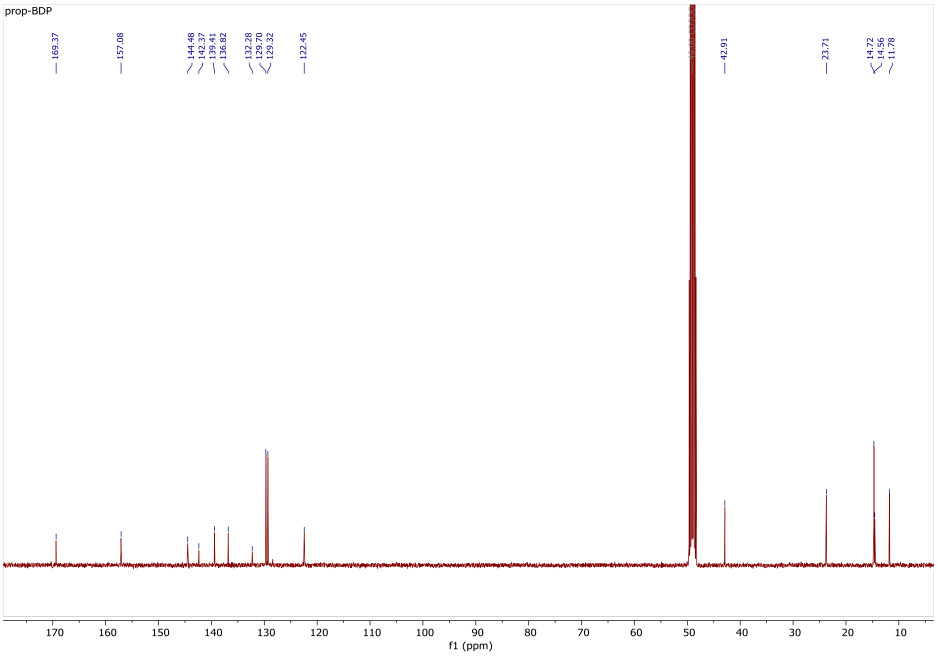

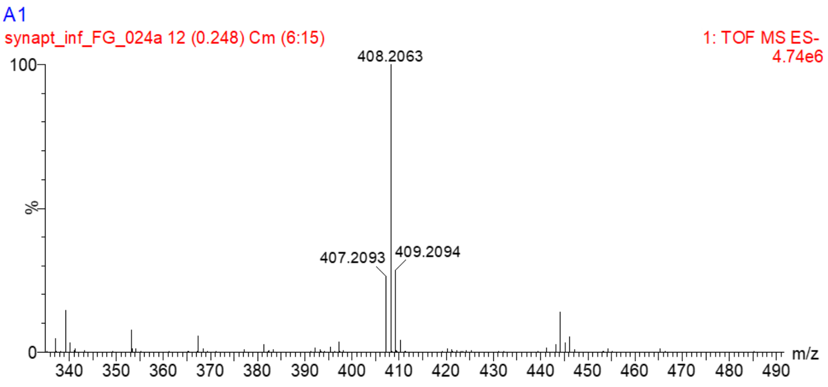

Figure S 20 ^{1}H NMR, ^{13}C NMR (DMSO-d$_6$) and mass spectra of compound **prop-BDP.**

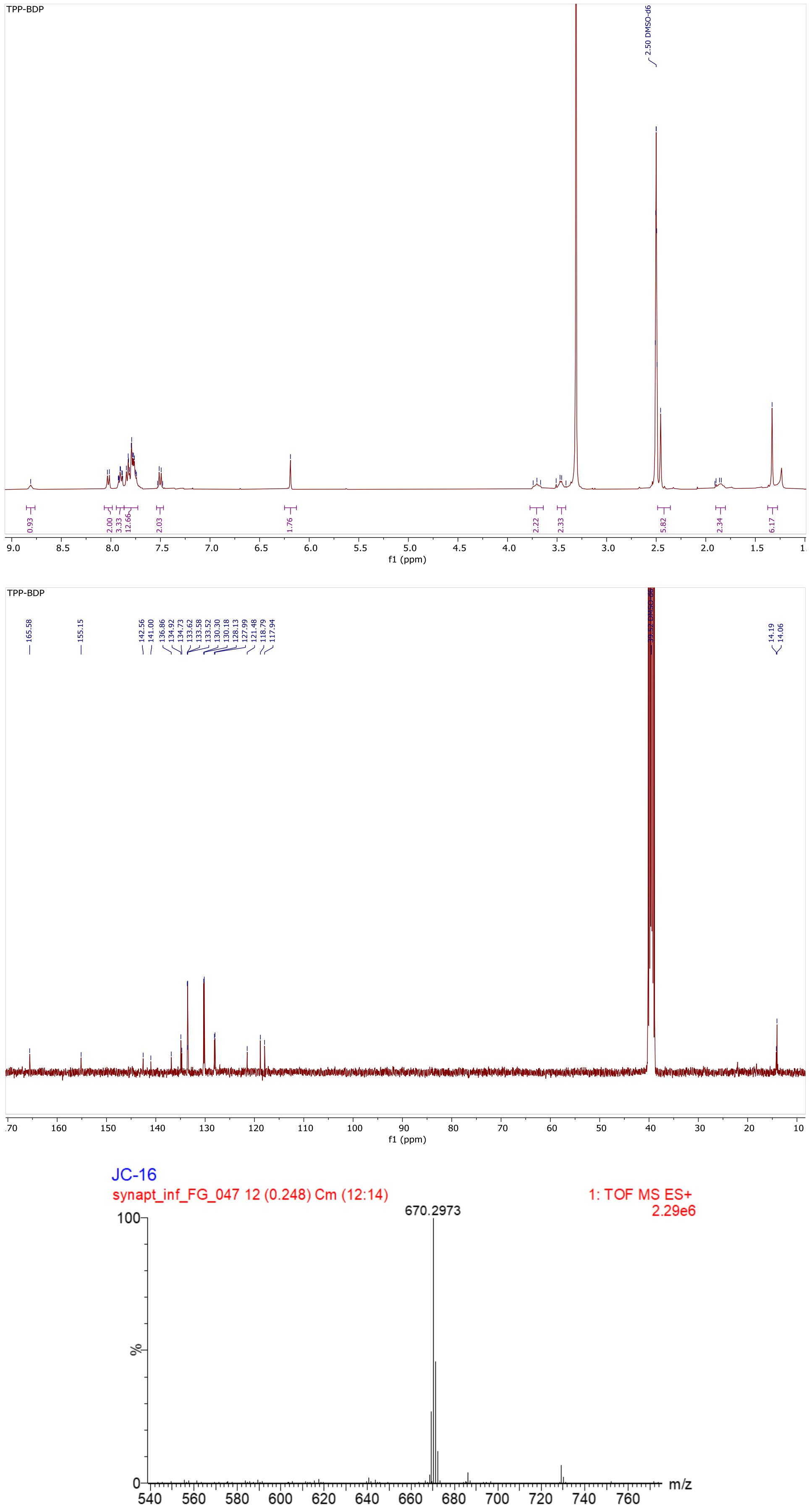

Figure S 21. ^{1}H NMR, ^{13}C NMR (DMSO-d$_6$) and mass spectra of compound **TPP-BDP**.

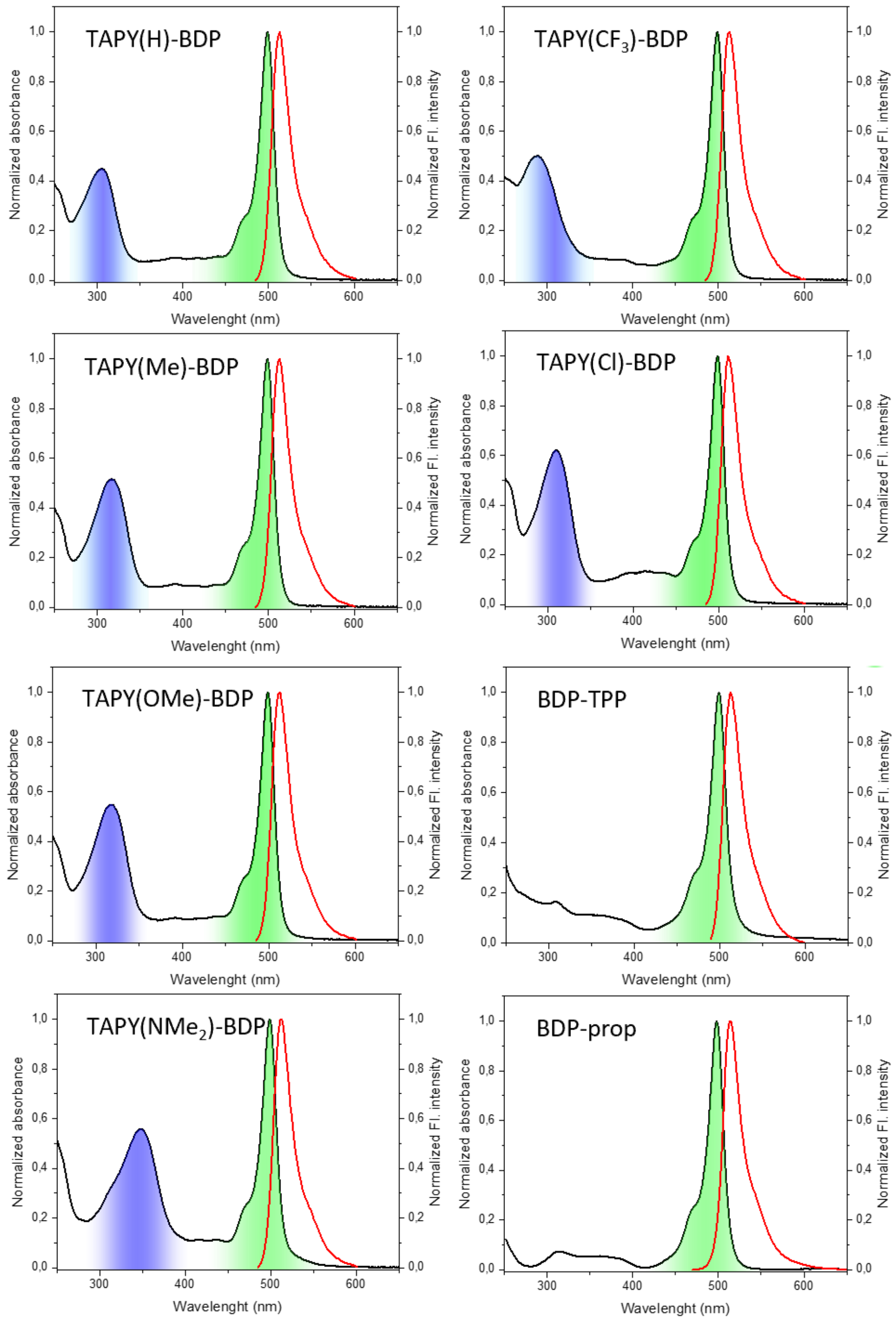

Figure S 22 Normalized absorption (black) and fluorescence emission (red) spectra of the TAPY-BDP, prop-BDP and TPP-BDP in acetonitrile at a concentration of 15 µM. The samples were excited at a wavelength of 470 nm. Note that two absorption bands have been highlighted in blue (TAPY) and green (BODIPY).

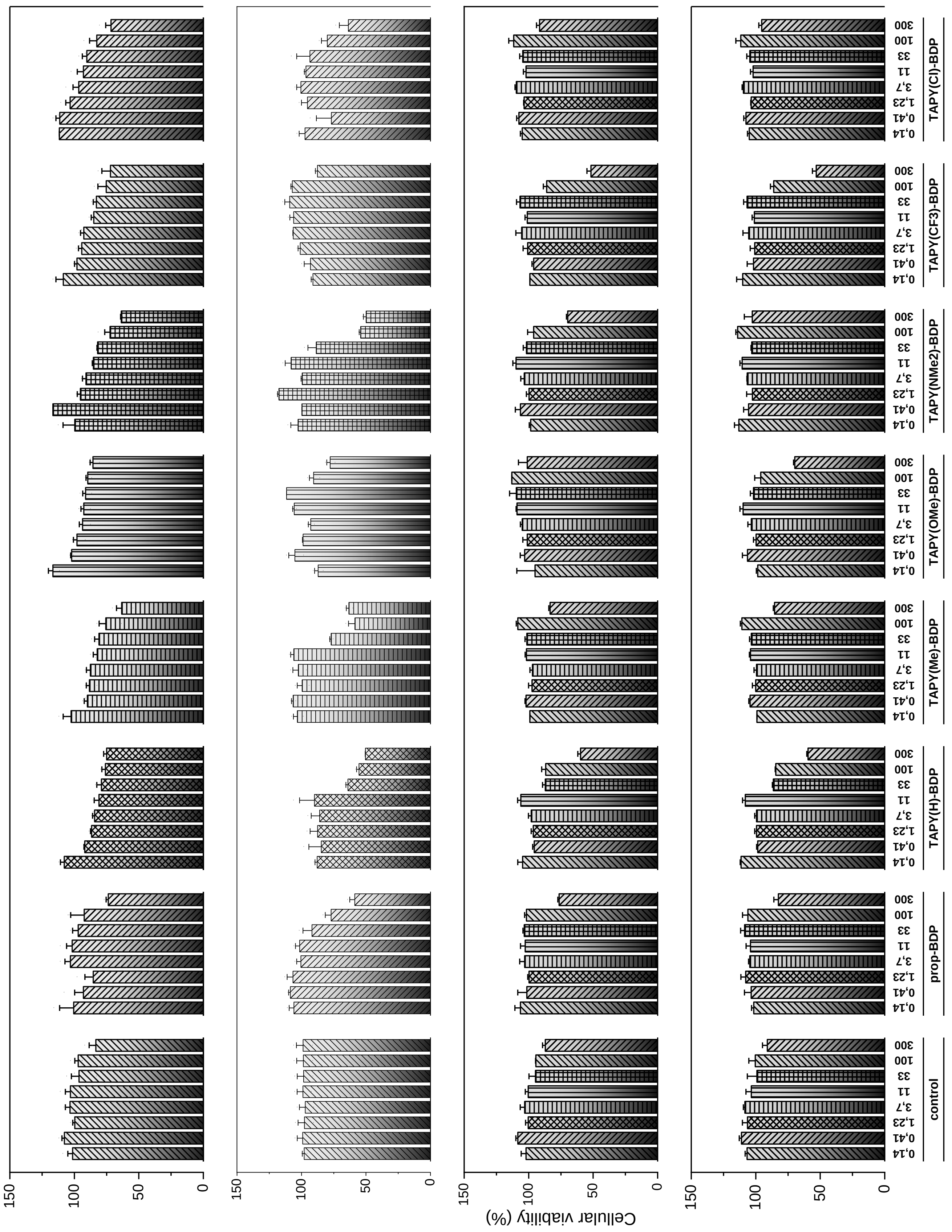

Figure S 23 MTT cell viability assays performed with four cell lines (MCF7, HMEC-1, A549, and HT29), exposed to different concentrations of TAPY-BDP and prop-BDP for 5 hours. The X-axis represents the compound concentrations in μM, ranging from 0.15 μM to 300 μM. Cell viability data were collected in triplicate and are presented as mean ± standard deviation.

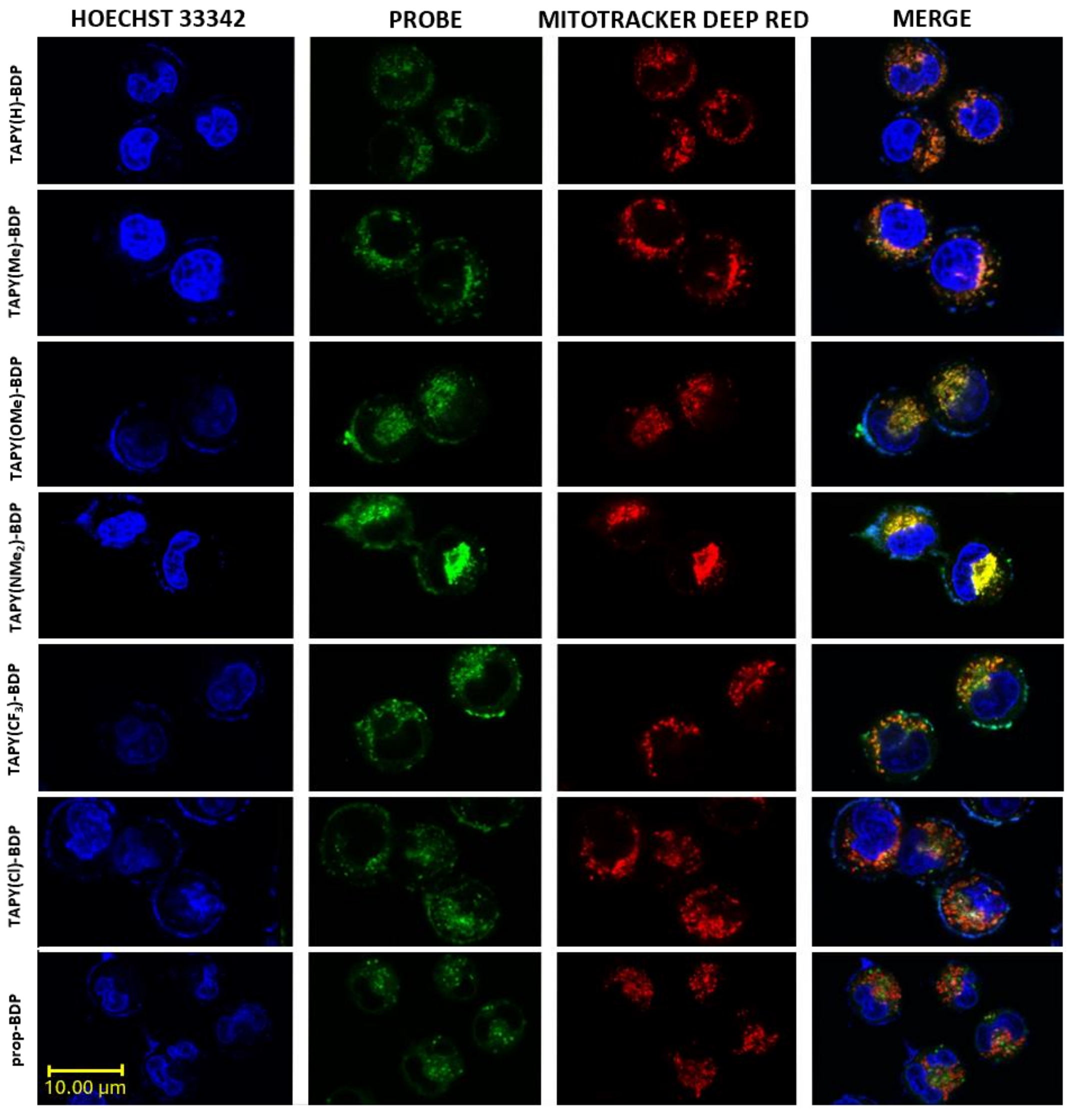

Figure S 24 (a) CLSM images of A549 cells incubated with 0.5 µM of TAPY-BDP, and prop-BDP probes, 100 nM of Mitotracker Deep Red, and Hoechst 33342 for 30 minutes at 37°C. Blue channel: excitation with 405 nm laser (Hoechst 33342); green channel: excitation with 488 nm laser (**TAPY-BDP** dyads and model compound); red channel: excitation with 633 nm laser (Mitotracker Deep Red FM). Also shown the overlay of green and red channels (merge).

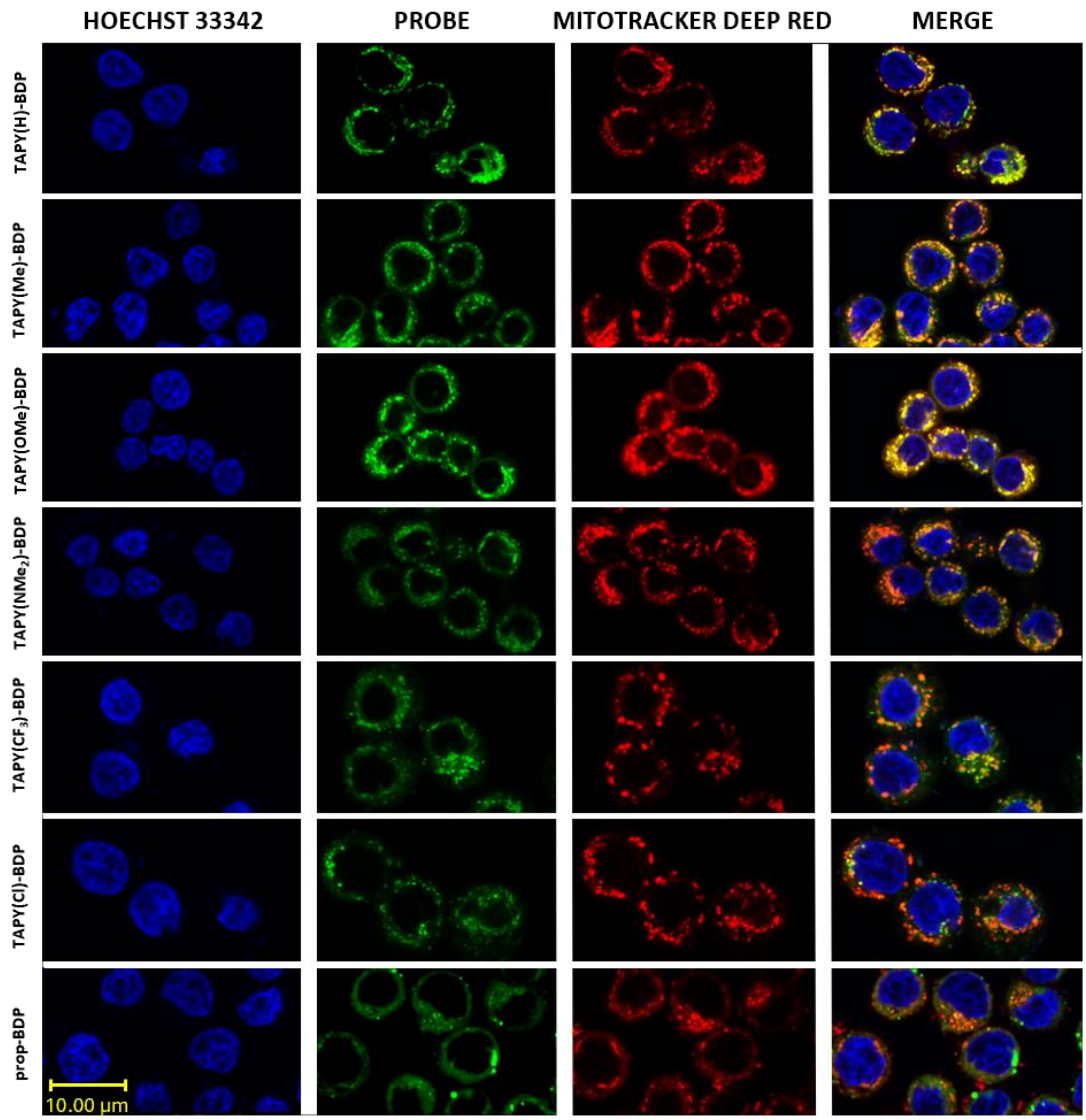

Figure S 25 (a) CLSM images of HT-29 cells incubated with 0.5 µM of TAPY-BDP, and prop-BDP probes, 100 nM of Mitotracker Deep Red, and Hoechst 33342 for 30 minutes at 37°C. Blue channel: excitation with 405 nm laser (Hoechst 33342); green channel: excitation with 488 nm laser (**TAPY-BDP** dyads and model compound); red channel: excitation with 633 nm laser (Mitotracker Deep Red FM). Also shown the overlay of green and red channels (merge).

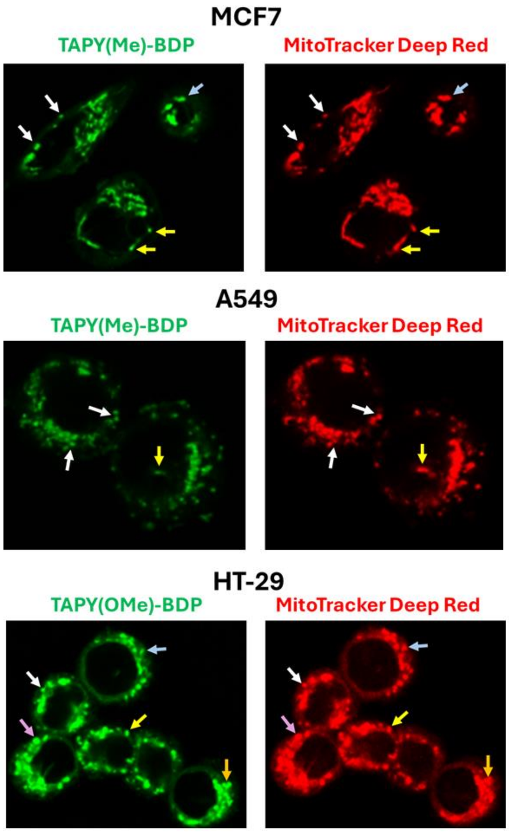

Figure S 26 Selected CLSM images of MCF7, A549 and HT-29 cells co-incubated with TAPY-BDP dyads (0.5 μM) and MTDR (100 nM) for 30 minutes at 37°C; green channel: excitation with 488 nm laser; red channel: excitation with 633 nm laser. In these pictures the resolution of certain cellular structures imaged with both types of probes can be compared. Details (arrows) recorded in the green channel are sometimes not appreciated in the red one.

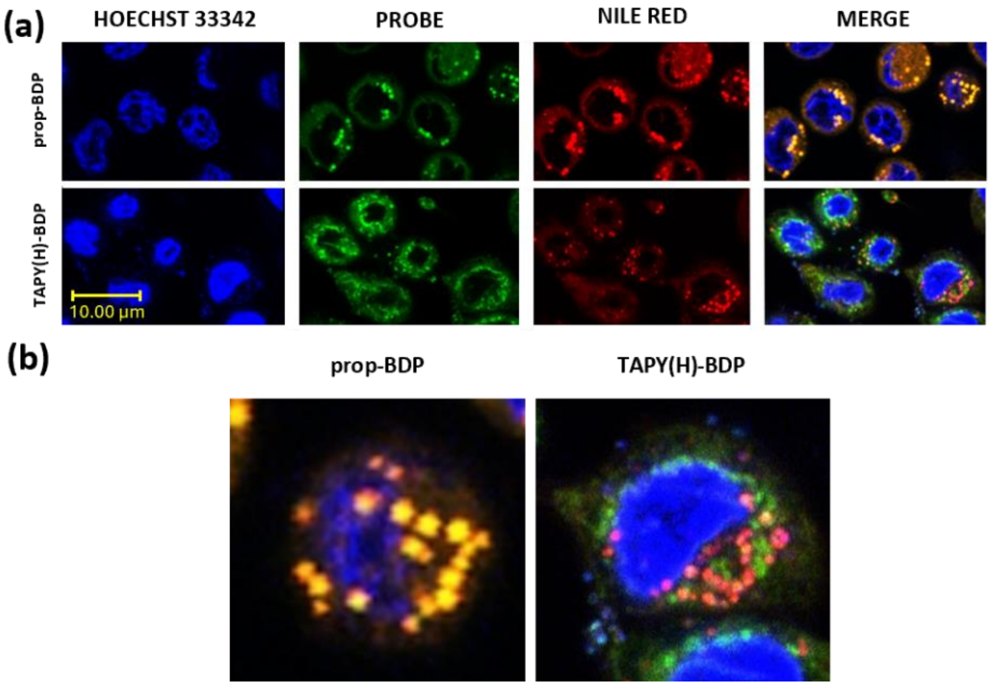

Figure S 27. (a) CLSM images. HT29 cells were incubated with 0.5 µM of TAPY(H)-BDP and prop-BDP, 2 µM of Nile Red, and Hoechst 33342 for 30 minutes at 37°C. Images were acquired using a Leica TCS SP8 confocal microscope with a 60x oil immersion objective. Blue channel: excitation with 405 nm laser (Hoechst 33342); green channel: excitation with 488 nm laser (probes); red channel: excitation with 561 nm laser (Mitotracker Deep Red). Also shown the overlay of green and red channels (merge). (b) Zoom of selected images (merge of green and red channels).

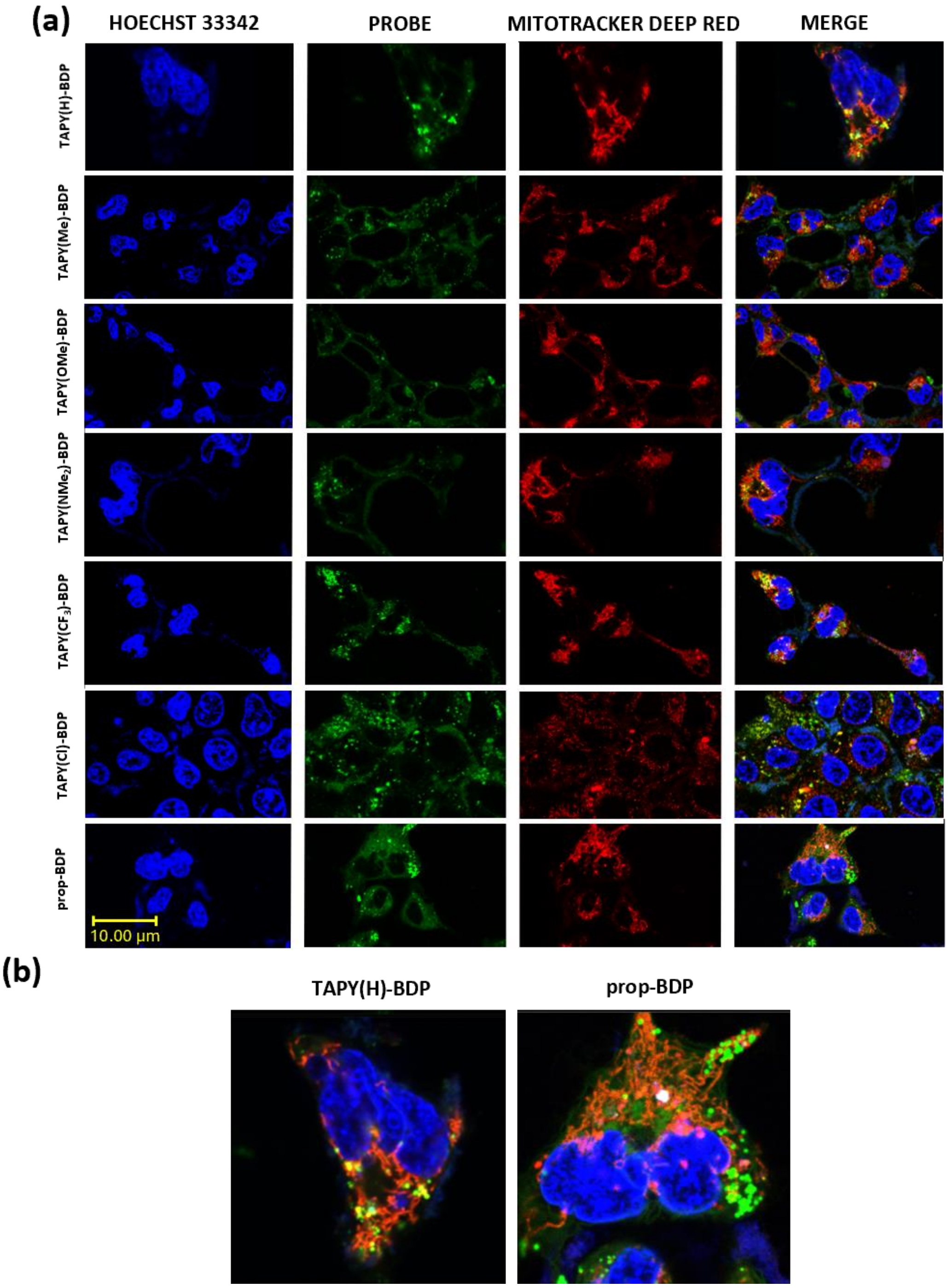

Figure S 28. (a) CLSM images of HEK293 cells incubated with 0.5 µM of **TAPY-BDP**, and **prop-BDP** probes, 100 nM of Mitotracker Deep Red, and Hoechst 33342 for 30 minutes at 37°C. Blue channel: excitation with 405 nm laser (Hoechst 33342); green channel: excitation with 488 nm laser (**TAPY-BDP** dyads and model compound); red channel: excitation with 633 nm laser (Mitotracker Deep Red). Also shown the overlay of green and red channels (merge). (b) Zoom of selected images (merge of green and red channels).

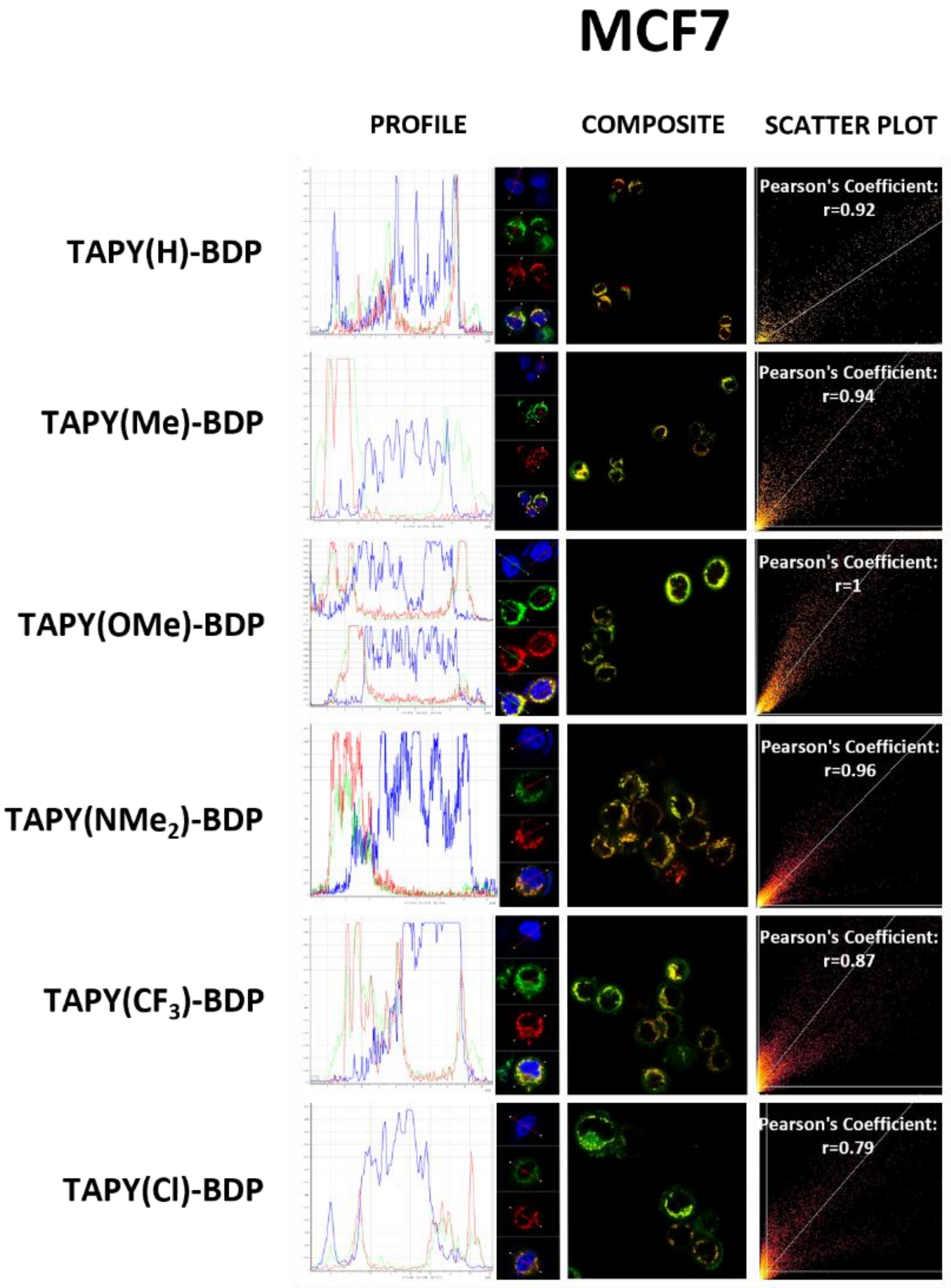

Figure S 29 Colocalization analysis of specific probes in MCF7 cells. Fluorescence intensity profiles for the blue (Hoechst 33342), green (TAPY's-BDP), and red (Mitotracker Deep Red) channels are shown in the first column. The composite colocalization image between the green probes and Mitotracker Deep Red is displayed in the third column. The fourth column presents scatter plots and Pearson's correlation coefficient (r), indicating the degree of colocalization between the probes and MTDR.

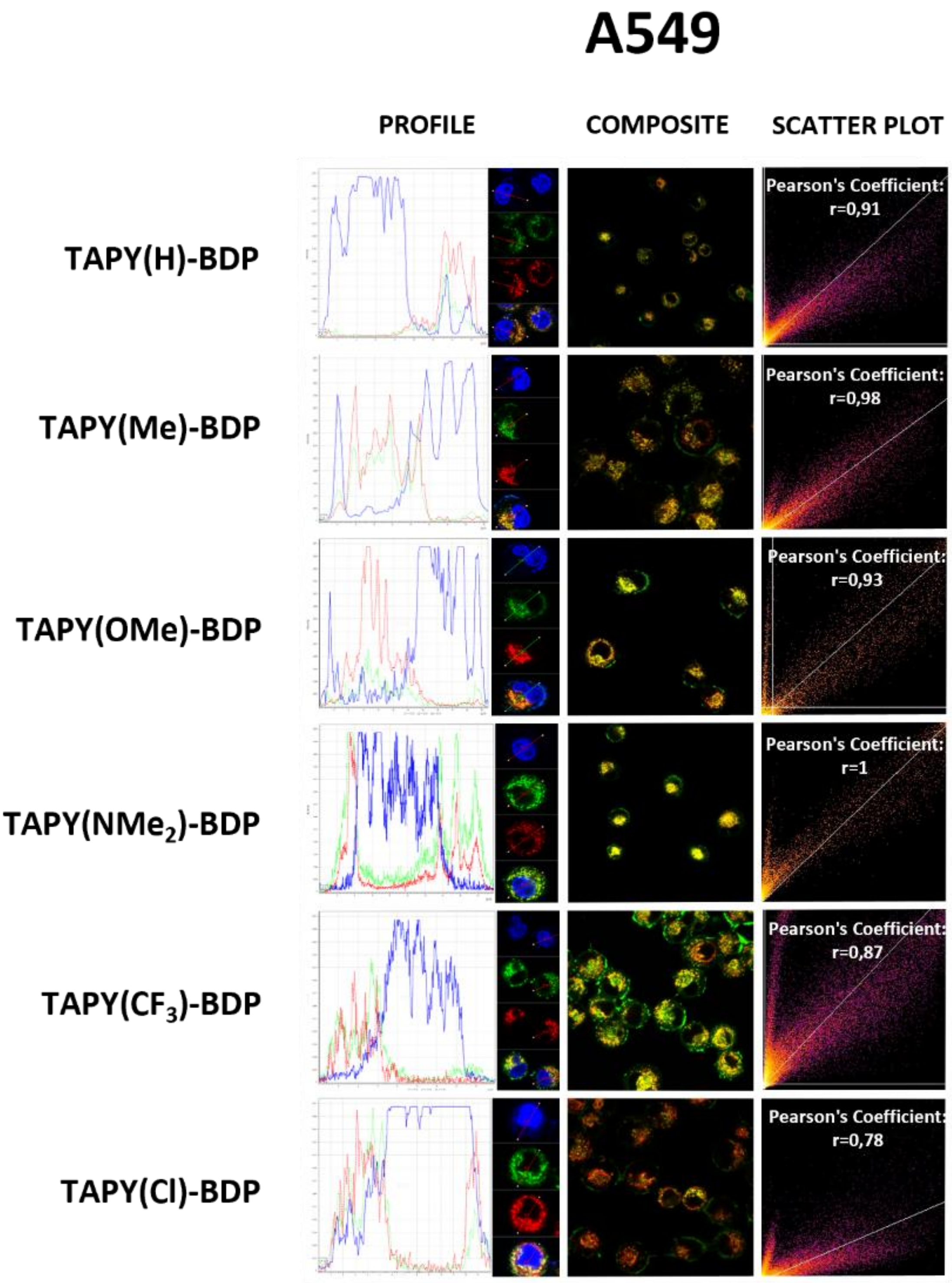

Figure S 30 Colocalization analysis of specific probes in A549 cells. Fluorescence intensity profiles for the blue (Hoechst 33342), green (TAPY's-BDP), and red (Mitotracker Deep Red) channels are shown in the first column. The composite colocalization image between the green probes and Mitotracker Deep Red is displayed in the third column. The fourth column presents scatter plots and Pearson's correlation coefficient (r), indicating the degree of colocalization between the probes and MTDR.

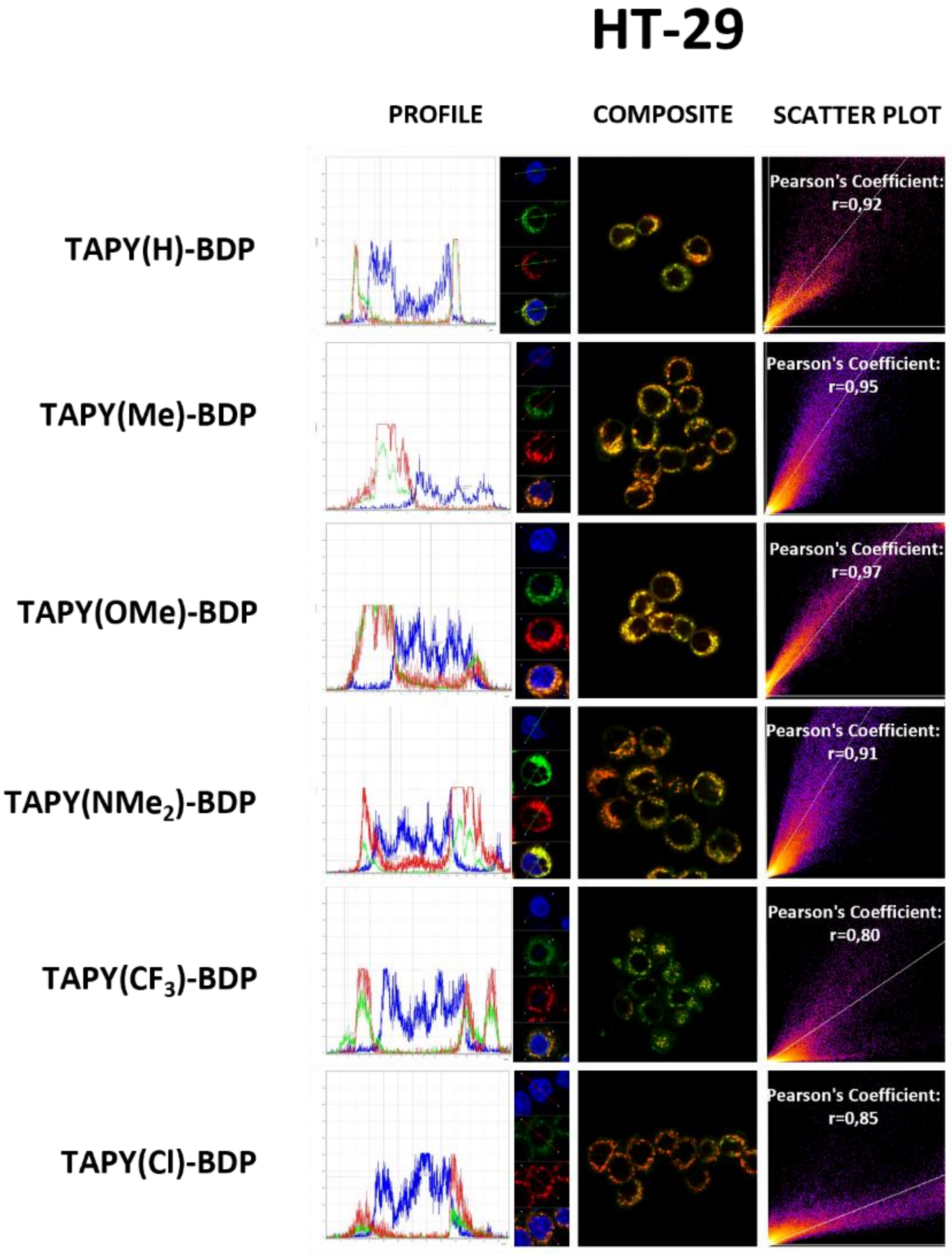

Figure S 31 Colocalization analysis of specific probes in HT-29 cells. Fluorescence intensity profiles for the blue (Hoechst 33342), green (TAPY's-BDP), and red (Mitotracker Deep Red) channels are shown in the first column. The composite colocalization image between the green probes and Mitotracker Deep Red is displayed in the third column. The fourth column presents scatter plots and Pearson's correlation coefficient (r), indicating the degree of colocalization between the probes and MTDR.

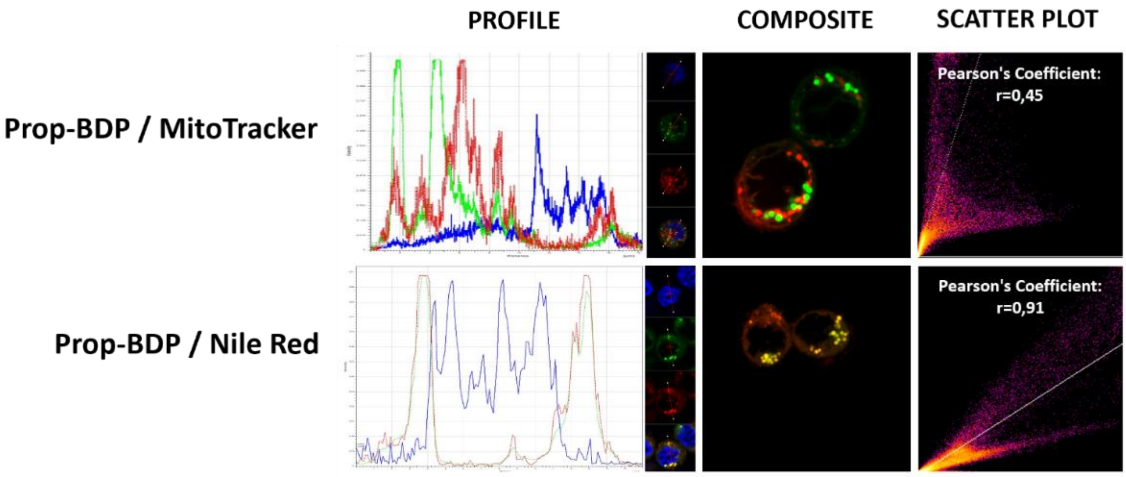

Figure S 32 Colocalization analysis of specific probes in MCF7 cells with Nile Red. Fluorescence intensity profiles for the blue (Hoechst 33342), green (prop-BDP), and red (Nile Red) channels are shown in the first column. The composite colocalization image between the green probes and Nile Red is displayed in the second column. The third column presents scatter plots and Pearson's correlation coefficient (r), indicating the degree of colocalization between the probes and Nile Red.

Supplemental references

[1] Miranda, M. A.; Izquierdo, M. A.; Galindo, F. Steady-State and Time-Resolved Studies on Oxetane Cycloreversion Using (Thia)pyrylium Salts as Electron-Transfer Photosensitizers. *Organic Letters* **2001**, *3*(13), 1965–1967.

[2] Katritzky, A. R.; Langthorne, R. T.; Patel, R. C.; Lhommet, G. Transformations of pyridiniums derived from amino-alcohols and from diamines. *Tetrahedron* **1981**, *37*(13), 2383–2390.

[3] Navarro-Barreda, D.; Bedrina, B.; Angulo-Pachón, C. A.; Miravet, J. F.; Pérez-Sala, D.; Galindo, F. Structure-performance relationships of four lysosomal markers used for the imaging of HT-29 cancer cells and a cellular model of lysosomal storage disease (Niemann-Pick C). *Dyes and Pigments* **2022**, *201*, 110236.

[4] Madak, J. T.; Cuthbertson, C. R.; Chen, W.; Showalter, H. D.; Neamati, N. Design, Synthesis, and Characterization of Brequinar Conjugates as Probes to Study DHODH Inhibition. *Chemistry – A European Journal* **2017**, *23*(56), 13875–13878.

[5] Muñoz Resta, I.; Bedrina, B.; Martínez-Planes, E.; Minguela, A.; Galindo, F. Detection of subcellular nitric oxide in mitochondria using a pyrylium probe: assays in cell cultures and peripheral blood. *Journal of Materials Chemistry B* **2021**, *9*(48), 9885–9892.
